# Comprehensive lipidomics analysis reveals the effects in goat milk from different ω-3 PUFA dietary supplementation by UPLC Q-TOF-MS/MS

**DOI:** 10.1016/j.fochx.2025.102581

**Published:** 2025-05-23

**Authors:** Jie Wang, Shiqian Ran, Xin Lv, Dan Wang, Hong Chen, Fang Wei

**Affiliations:** a Key Laboratory of Oilseeds Processing of Ministry of Agriculture, Hubei Key Laboratory of Lipid Chemistry and Nutrition, Oil Crops Research Institute of the Chinese Academy of Agricultural Sciences, Wuhan, Hubei 430062, PR China; b Hubei Hongshan Laboratory, Wuhan, Hubei 430070, PR China; c Zunyi Rural Development Service Center, Zunyi, Guizhou 563000, PR China; d Hubei Key Laboratory of Animal Nutrition and Feed Science, Wuhan Polytechnic University, Wuhan 430023, PR China

**Keywords:** Lipidomics, Goat milk, Dietary supplemented, Ω-3 polyunsaturated fatty acid

## Abstract

Goat milk has gained significant attention due to its unique nutritional properties. In this study, we conducted a comprehensive lipidomics analysis of goat milk fed by a normal diet (C), dietary supplement with *Schizochytrium* sp. (S), flax seed (F), and their mixture (M) using UPLC Q-TOF-MS/MS. A total of 638 lipid molecules covering 16 subclasses were identified, with TG constituting over 97 % of the total lipids. Dietary supplementation significantly reduced glycerolipids (GL) content while markedly increasing ω-3 polyunsaturated fatty acid (PUFA) levels, with group M showing the most pronounced effects on enhancing ω-3 PUFA content. The percentages of DHA-TG and DHA-GPL increased by more than 10-fold and 4-fold, respectively. Flax seed specifically increased ALA containing lipids, whereas *Schizochytrium* sp. predominantly enriched DHA and DPA containing lipids. Multivariate statistical analysis revealed distinct lipid profiles among the groups, highlighting the significant impact of dietary supplementation on lipid composition. In conclusion, this study provides a robust theoretical foundation for the production, nutritional evaluation, identification, and application of ω-3 PUFA-enriched goat milk.

## Highlights

• Goat milk obtained by different ω-3 PUFA dietary supplementation.
• Lipids of goat milk were comprehensive analyzed by UPLC Q-TOF-MS/MS.
• A total of 638 lipid molecular species of 16 subclasses were characterized.
• Dietary supplementation led to a significant decrease in GL of goat milk.
• Group M had the best effect on improving ω-3 PUFA lipids in goat milk.

## 1 Introduction

Goat milk has garnered significant attention as a valuable source of dairy products due to its unique nutritional properties. Recognized for its high lipid content and rich protein profile, which many consumers find appealing. In recent years, global production of goat milk has steadily increased, this expansion has resulted in broader utilization of goat milk in both fresh and processed products, including cheese and yogurt (Miller & Lu, 2019; Wu et al., 2023). Comparative studies with cow milk, reveal that goat milk shares similar calcium and phosphate levels, but exhibits smaller milk fat globules membrane (MFGM). It also contains higher concentrations of medium-chain fatty acids (MCFA) and medium-chain triglycerides (MCT) (Gallier et al., 2020). These distinctive characteristics contribute to easier digestion and absorption, potential relief from gastric ulcers, and improved glucose homeostasis in diabetic individuals (Han et al., 2022). Furthermore, research indicates goat milk positively influences intestinal flora (Liu & Zhang, 2022). Additionally, goat milk has a lower content of αs1-casein in its protein composition compared to cow milk, which reduces allergy risks associated with conventional dairy consumption while enhancing functional versatility (Hodgkinson et al., 2012).

Lipid composition serves as a critical indicator for evaluating the nutritional and sensory quality of goat milk. Of particular interest are specific fatty acids, especially ω-3 polyunsaturated fatty acids (ω-3 PUFA), which have received considerable attention due to their substantial nutritional benefits and diverse health-promoting properties. These bioactive compounds are known to reduce risks of chronic diseases such as cardiovascular disorders and neurodegenerative conditions, establishing ω-3 PUFA as an essential component of a balanced diet (Shahidi & Ambigaipalan, 2018). Key ω-3 PUFA include α-linolenic acid (ALA, C18:3), stearidonic acid (SDA, C18:4), eicosapentaenoic acid (EPA, C20:5), docosapentaenoic acid (DPA, C22:5), and docosahexaenoic acid (DHA, C22:6). According to the United Nations Food and Agricultural Organization (FAO), the recommend daily intake ranges of 0.5–0.6 % ALA to prevent deficiency symptoms, with total ω-3 PUFA intake advised at 0.5–2 %. Clinical evidence demonstrates ω-3 PUFA efficacy in managing cardiovascular diseases and diabetes. These fatty acids are also crucial for the development of the human brain and nervous system, influencing cognitive and optic nerve development in infants and positively impacting the health of pregnant women and the elderly (Aldoori et al., 2022; Costantini et al., 2017).

Breast milk remains universally recognized as the optimal source for human infants, though practical limitations prevent universal breastfeeding. In these circumstances, infant formula serves as the best alternative to meet their nutritional needs (Yao et al., 2016). The European Food Safety Authority (EFSA) concluded in 2012 that goat milk qualifies for use in infant and follow-up formulas (EFSA Panel on Dietetic Products, N. and Allergies, 2012). However, it is important to note that breast milk contains higher levels of oleic acid (OA), DHA, arachidonic acid (AA), linoleic acid (LA), and ALA compared to goat milk (Wang et al., 2020). Consequently, supplementing goat milk with these fatty acids, particularly ω-3 PUFA, is essential to make it more suitable for infant formula and enhance its nutritional value. The lipid profile and overall nutritional composition of ruminant milk are closely regulated by dietary and genetic factors (Wu et al., 2023). Conventional PUFA enrichment strategies typically utilize ALA-rich oilseeds (rape seed and flax seed), fish oil, and microalgae (*Crypthecodinium cohnii and Schizochytrium* sp.). Crucially, the choice of dietary PUFA source directly impacts the resultant fatty acid profile in milk. Due to the low desaturase and elongase activities required for the conversion of ALA to DHA, supplementation with ALA alone results in lower DHA levels (Ehr et al., 2017). While fish oil serves as a viable PUFA source, its use often incurs high production costs due to its high price. Microalgae emerge as a cost-effective alternative with superior yield potential. It has been demonstrated that supplementing the diets of cows with ω-3 PUFA-rich microalgae significantly increases the DHA content of the resulting milk (Bionaz et al., 2020; Liu, Guo, et al., 2020). Other sources of ω-3 PUFA dietary supplementation have also been studied. For example, Wu et al. obtained DHA-rich eggs by feeding them with flax seed and *Schizochytrium* sp. (Wu et al., 2020). However, the effects of different dietary PUFA supplementation sources on goat milk have not been thoroughly studied.

Previous studies on ω-3 PUFA supplementation effects on milk composition have predominantly employed gas chromatography (GC) to monitor fatty acid changes. For example, Zhu et al. fed goats with DHA microalgae and found that the DHA content in goat milk increased significantly by GC detection (Zhu et al., 2022). However, the bioavailability and health benefits of ω-3 PUFA are influenced by their chemical form. Phospholipids form ω-3 PUFA are reported to be more efficiently targeted to cross the blood-brain barrier compared to triglycerides (TG) form ω-3 PUFA (Chouinard-Watkins et al., 2019). Thus, a comprehensive analysis of milk lipids, including their fingerprints and molecular characterization, is essential. Over recent decades, the rapid advancement of mass spectrometry (MS) technology has made lipidomics a widely adopted approach among researchers, enabling the study of lipid interactions and metabolic pathways at the molecular level. For instance, Li et al. assessed the lipid profiles of goat milk, soymilk, and bovine milk using ultraperformance liquid chromatography coupled with Q-Exactive Orbitrap Mass Spectrometry (UPLC Q-E Orbitrap MS), identifying a total of 13 lipid subclasses (Li et al., 2017). Similarly, Wang et al. employed ultraperformance liquid chromatography coupled with quadrupole time-of-flight mass spectrometry (UPLC-Q-TOF-MS) to detect lipids in human, bovine, and caprine milk, identifying hundreds of lipid molecules (Wang et al., 2020). Therefore, it is necessary to thoroughly analyze the changes of lipid molecular species in goat milk caused by ω-3 PUFA supplementation from different sources by these advanced mass spectrometry techniques.

In this study, comprehensive lipidomic analysis of goat milk using UPLC Q-TOF-MS/MS to investigate the effects of different ω-3 PUFA dietary supplementation sources (flax seed, *Schizochytrium* sp., and their mixture) on lipid molecular species. This study reveals the distinct effects of these supplementation sources on the distribution of ω-3 PUFA in goat milk, particularly the synergistic effect of mixed supplementation on enhancing ω-3 PUFA levels. Practical implications for functional dairy development, particularly infant formula, by linking lipid molecular profiles to nutritional optimization strategies. These findings establish a robust foundation for producing ω-3 PUFA-fortified goat milk with enhanced health benefits.

## 2 Materials and methods

### 2.1 Reagents and materials

All the chemicals and reagents in this experiment were LC − MS grade and purchased from Thermo Fisher Scientific (Rockford, IL) and CNW (Düsseldorf, Germany). Fatty acid (FA) internal standard (C17:0) and 37 FA methyl esterification standards were purchased from Sigma-Aldrich (Dorset, U.K.). Lipid internal standards SPLASH Lipidomix was purchased from Avanti Polar Lipids (Alabaster, Alabama, USA). The internal standard solution was prepared and the concentrations were 10 μg/mL.

### 2.2 Ethical statements

The animal experiments in this study were approved by the Animal Welfare and Ethics Committee of Wuhan Polytechnic University (Animal Welfare Number: WPU201919992). All procedures were conducted in accordance with the animal research guidelines established by Wuhan Polytechnic University and fully complied with the relevant national laws and regulations of China, as well as international standards for animal welfare and ethical treatment. The goats used in the study were fed and maintained in strict compliance with the ethical standards set forth by the Animal Welfare and Ethics Committee of Wuhan Polytechnic University.

### 2.3 Animals, diets, and management

16 Yichang White Goats (body weight about 20–25 kg and age between 1.5 and 2.5 years old) were provided by the Enshi Prefecture Academy of Agricultural Sciences of Hubei Province and divided randomly into four groups. The experimental diets include a normal diet (group C), a normal diet incorporated with 10 % flax seed (by weight of diet, group F), a normal diet incorporated with 10 % *Schizochytrium* sp. (group S), and a normal diet incorporated with 10 % *Schizochytrium* sp. and 10 % flax seed (group M). The additional amount was in accordance with our previous research (Ran et al., 2023). The *Schizochytrium* sp. was obtained from Cabio Biotech (Wuhan) Co., Ltd.. Flax seed was sourced from the Hebei Academy of Agriculture and Forestry Science. The overall experimental period lasted for 5 weeks, in which the first week was the adaptation period. The feeding of various diets lasted 4 weeks and each goat was scheduled to be fed with 500 g/d experimental diets. All goats should be examined to make sure that they are in healthy condition. The other aspects of their care and management are carried out by the regulations of the farm. Ingredients of experimental diets and FA composition of flax seed and *Schizochytrium* sp. are given in Table S1.

### 2.4 Sample collection

At last, 16 goat milk samples were collected from group C, group F, group S and group M in the winter of 2021. After the feeding, all the milk samples were shipped with dry ice as a refrigerant and then stored at −80 °C until analysis.

### 2.5 FA composition analysis

FA composition analysis was determined in accordance with the previous reference (Li et al., 2022). The goat milk sample was thawed. 300 μL of goat milk sample was accurately weighed (*n* = 4) with 70 μL C17:0 added. 2 mL concentrated sulfuric acid/methanol (5 %, *v*/v), 300 μL toluene was added, then under 90–95 °C for 1.5 h. Afterward, 2 mL sodium chloride (0.9 %, *w*/w) was added, and the formed FA methyl esters (FAME) were extracted with 1 mL hexane for GC analysis.

Analyses of FAME were carried out on a GC (Agilent 7890 N, Palo Alto, CA, USA) equipped with flame ionization detection (FID) and a capillary column (HP-FFAP, 30 m × 0.25 mm × 0.25 μm) purchased from Agilent (Agilent J & W GC Columns). The oven temperature was first controlled at 150 °C, then heated up to 210 °C at 10 °C/min, and held for 7 min, further raised to 230 °C at 20 °C/min and kept for 6 min. Nitrogen was used as carrier gas at an inlet pressure of 1.7 × 10^5^ Pa. Other chromatographic operating conditions were as follows: injector temperature, 260 °C; split ratio 20:1, linear flow for carrier gas 20 mL/min.

### 2.6 Lipid extraction

Some modifications are based on previous research in our lab (Wu et al., 2020). Milk sample thawing: The goat milk samples of different experimental groups are placed on the ice surface to thaw and shaken evenly. Protein precipitation: Accurately take 80 μL of sample (*n* = 4), and add 2 mL of methanol at −20 °C to precipitate protein for 12 h. Lipid extraction: add 100 μL of 10 mg/mL lipid internal standard to the sample; add 2 mL of chloroform, vortex for 1 h; add 2 mL of chloroform, 1.4 mL of ultrapure water, vortex for 15 min; centrifuge at 4 °C, 5000 r/min for 10 min, collect the bottom phase. Add another 4 mL of chloroform to the supernatant, vortex for 15 min, centrifuge at 4 °C, 5000 r/min for 10 min, and repeat three times. The collected bottom phase was evaporated with nitrogen and redissolved in 1 mL chloroform/methanol (2:1, *v*/v).

Glycerophospholipids (GPL) Extraction: Activate the Si-SPE column with 6 mL of n-hexane. Perform sequential elution using 2 mL of n-hexane/ether (8:2, *v*/v) and 2 mL of n-hexane/ether (1:1, *v*/v) to leach out the neutral lipids. Discard the eluent, rinse the column with 6 mL of methanol and 2 mL of chloroform/methanol/water (3:5:2, v/v/v), and collect the eluate. Evaporate the solvent using nitrogen and reconstitute the samples with 100 μL of chloroform solution. Filter through a 0.22 μL organic membrane for UPLC Q-TOF-MS/MS.

### 2.7 UPLC Q-TOF-MS/MS analysis

UPLC-Q-TOF-MS analyses were carried out with a UPLC 30 A system equipped with a Kinetex C18 column (100 mm × 2.1 mm, 2.6 μm, Phenomenex), coupled with a high-resolution mass spectrometer (Q-TOF 6500, AB Sciex, Concord, ON, Canada). The flow rate was 400 μL/min, and the column temperature was maintained at 55 °*C. mobile* phases A and B were composed of water/MeOH/ACN (1:1:1, v/v/v) and IPA/ACN (5:1, v/v), both containing 5 mM ammonium acetate, respectively.

Lipid separation was achieved by gradient elution: 0–0.5 min, 80 % A; 0.5–1.5 min, 60 % A, 1.5–3 min, 40 % A; 3–13 min, 2 % A; 13–13.1 min, 80 % A, 13.1–17 min, 80 % A. The MS data were collected in both positive electrospray ionization (ESI+) and negative electrospray ionization (ESI−) modes at spray voltages of 5500 and − 4500 V with a mass range from 50 to 1200 *m*/*z*, respectively. In the positive-ion mode and the negative-ion mode, the declustering potential (DP) was set to 80 V and − 80 V, and the collision energy (CE) was set to 30 V and − 30 V, respectively.

### 2.8 Data processing and statistical analysis

Data processing was performed using freely available MS-DIAL (version 4.60, http://prime.psc.riken.jp/Metabolomics_Software/MS-DIAL/index2.html) and commercially available software packages, PeakView, MasterView and MultiQuant (AB Sciex, Concord, ON, Canada). All experiment data were presented as mean ± standard deviation (SD) (*n* = 4). The significance of differences in data was tested through analysis of variance (ANOVA) using the SPSS 20.0 software package (SPSS Inc., Chicago, IL, USA) Principal component analysis (PCA), partial least squares-discriminate analysis (PLS-DA) and lipid metabolism pathway analysis were performed by Web-based MetaboAnalyst 5.0 online (http://www.metaboanalyst.ca).

## 3 Result and discussion

### 3.1 FA composition of goat milk

Lipid composition constitutes a pivotal quality parameter in goat milk evaluation, with FA being its fundamental constituent. Environmental and dietary conditions exert significant influence on the FA profile of goat milk. GC was employed to analyze the fatty acid composition of goat milk derived from different diets (Table 1), covering a range from C6 to C24. A total of 24 FA were detected. We observed that dietary supplementation led to a significant reduction (*p* < 0.05) in total FA content compared to the control diet (group C). This reduction may be attributed to the impact of high unsaturated FA in *Schizochytrium* sp. and flax seed on microbial fermentation in the rumen of goats, which in turn affects the synthesis of acetic acid, a primary carbon source for lipid synthesis (Lévesque et al., 2022). Palmitic acid (C16:0) and oleic acid (C18:1) were the most abundant FA, collectively comprising >40 % of total FA. The inclusion of flax seed in the diet significantly increased (*p* < 0.05) the relative content of ALA in group F and significantly decreased (*p* < 0.05) the content of saturated fatty acids (SFA) in both groups F and M. This is attributed to the high ALA content in flax seed. The addition of *Schizochytrium* sp. to the diet significantly reduced (*p* < 0.05) monounsaturated fatty acids (MUFA) in groups S and M. The most pronounced change due to dietary supplementation was observed in polyunsaturated fatty acids (PUFA), with the relative content of PUFA more than doubling in group M (11.13 %) compared to group C (4.71 %) when *Schizochytrium* sp. and flax seed were combined. Among PUFA, ω-3 PUFA showed the most significant effect, with group M (4.74 %) being over tenfold higher than group C (0.44 %). Moreover, the ω-6/ω-3 PUFA ratio also decreased significantly (*p* < 0.05) after supplementation, to 1.73 % in group S and 1.35 % in group M, aligning with the recommended health-beneficial range of 1–2 (Simopoulos, 2009), and a lower ω-6/ω-3 ratio has a positive anti-inflammatory effect (Wei et al., 2021). Undoubtedly, these findings confirm that strategic dietary supplementation enhances both nutritional value and health benefits of goat milk.

**Table 1:** FA composition of different goat milk experimental groups (%).

FA	C	F	S	M
C6:0	2.15 ± 0.25^a^	2.69 ± 0.05^b^	2.58 ± 0.17^b^	2.63 ± 0.29^b^
C8:0	3.34 ± 0.38^a^	3.82 ± 0.21^ab^	3.99 ± 0.31^b^	3.82 ± 0.27^ab^
C10:0	10.59 ± 1.17^a^	11.95 ± 0.28^ab^	13.13 ± 0.82^b^	10.99 ± 0.61^a^
C11:0	0.11 ± 0.02^a^	0.16 ± 0.02^b^	0.14 ± 0.02^b^	0.15 ± 0.01^b^
C12:0	5.51 ± 0.76^b^	4.99 ± 0.2^ab^	5.47 ± 0.47^b^	4.28 ± 0.35^a^
C14:0	11.23 ± 0.96^a^	8.42 ± 0.51^a^	8.63 ± 1.03^a^	8.12 ± 1.22^b^
C14:1	0.32 ± 0.04^c^	0.14 ± 0^b^	0.09 ± 0.01^a^	0.12 ± 0.02^ab^
C15:0	0.78 ± 0.11^a^	0.91 ± 0.03^a^	0.79 ± 0.09^a^	0.82 ± 0.12^a^
C15:1	0.38 ± 0.04^b^	0.34 ± 0^ab^	0.27 ± 0.04^a^	0.29 ± 0.06^b^
C16:0	29.84 ± 3.19^b^	21.36 ± 1.08^a^	24.68 ± 2.5^b^	23.03 ± 2.43^a^
C16:1	1.19 ± 0.09^b^	0.84 ± 0.05^a^	0.77 ± 0.08^a^	0.77 ± 0.18^a^
C17:0	0.41 ± 0.04^a^	0.47 ± 0.02^a^	0.61 ± 0.06^b^	0.4 ± 0.04^a^
C17:1	0.27 ± 0.03^c^	0.19 ± 0^a^	0.23 ± 0.04^b^	0.15 ± 0.01^a^
C18:0	3.89 ± 0.27^a^	8.71 ± 0.44^b^	9.11 ± 1.42^b^	8.24 ± 1.02^b^
C18:1	23.8 ± 0.81^a^	24.89 ± 1.13^a^	20.5 ± 4.28^b^	19.89 ± 3.05^a^
C18:2	3.68 ± 0.46^b^	4.32 ± 0.57^b^	2.89 ± 0.44^a^	3.79 ± 0.17^b^
C18:3 ω-3	0.31 ± 0.03^a^	0.76 ± 0.04^d^	0.38 ± 0.05^b^	0.49 ± 0.01^c^
C20:1 ω-9	1.49 ± 0.24^a^	3.12 ± 0.24^b^	2.1 ± 0.15^a^	3.98 ± 0.67^c^
C20:2 ω-6	0.39 ± 0.04^a^	0.75 ± 0.06^b^	0.51 ± 0.05^a^	1.52 ± 0.16^c^
C20:4 ω-6	0.2 ± 0.03^a^	0.23 ± 0.01^a^	0.45 ± 0.06^b^	1.07 ± 0.18^c^
C20:5 ω-3	0 ± 0^a^	0 ± 0^a^	0.37 ± 0.05^b^	0.9 ± 0.19^c^
C22:6 ω-3	0.13 ± 0.02^a^	0.33 ± 0.04^a^	1.48 ± 0.12^b^	3.36 ± 0.19^c^
C24:0	0 ± 0^a^	0.51 ± 0.05^b^	0.52 ± 0.1^b^	0.56 ± 0.02^b^
C24:1	0 ± 0^a^	0.12 ± 0.01^b^	0.32 ± 0.03^c^	0.66 ± 0.04^d^
∑MCFA(6–12)	21.7 ± 2.05^a^	23.62 ± 0.34^ab^	25.3 ± 1.58^b^	21.87 ± 1.22^a^
∑LCFA(≥13)	78.3 ± 2.05^b^	76.38 ± 0.34^ab^	74.7 ± 1.58^a^	78.13 ± 1.22^b^
∑SFA	67.85 ± 1.33^ab^	63.98 ± 1.93^a^	69.64 ± 3.57^b^	63.03 ± 3.38^a^
∑MUFA	27.44 ± 0.95^b^	29.63 ± 1.3^c^	24.29 ± 4.12^a^	25.84 ± 3.72^a^
∑PUFA	4.71 ± 0.44^a^	6.39 ± 0.64^b^	6.07 ± 0.63^b^	11.13 ± 0.39^c^
∑ω-6PUFA	4.27 ± 0.44^a^	5.3 ± 0.64^b^	3.85 ± 0.44^a^	6.39 ± 0.2^c^
∑ω-3PUFA	0.44 ± 0.04^a^	1.09 ± 0.01^b^	2.22 ± 0.19^c^	4.74 ± 0.28^d^
ω6/ω3	9.82 ± 1.32^c^	4.86 ± 0.58^b^	1.73 ± 0.08^a^	1.35 ± 0.08^a^
Total g/L	58.10 ± 2.68^a^	42.74 ± 3.01^b^	45.91 ± 2.25^b^	44.64 ± 0.75^b^

a-d Mean values in the same row (corresponding to the same parameter) not followed by a common letter differ significantly (*p* < 0.05). C: control, F: flax seed, S: *Schizochytrium* sp., M: mixture of *Schizochytrium* sp. and flax seed.

### 3.2 Comprehensive lipidomics profile of goat milk

**Fig. 1:**
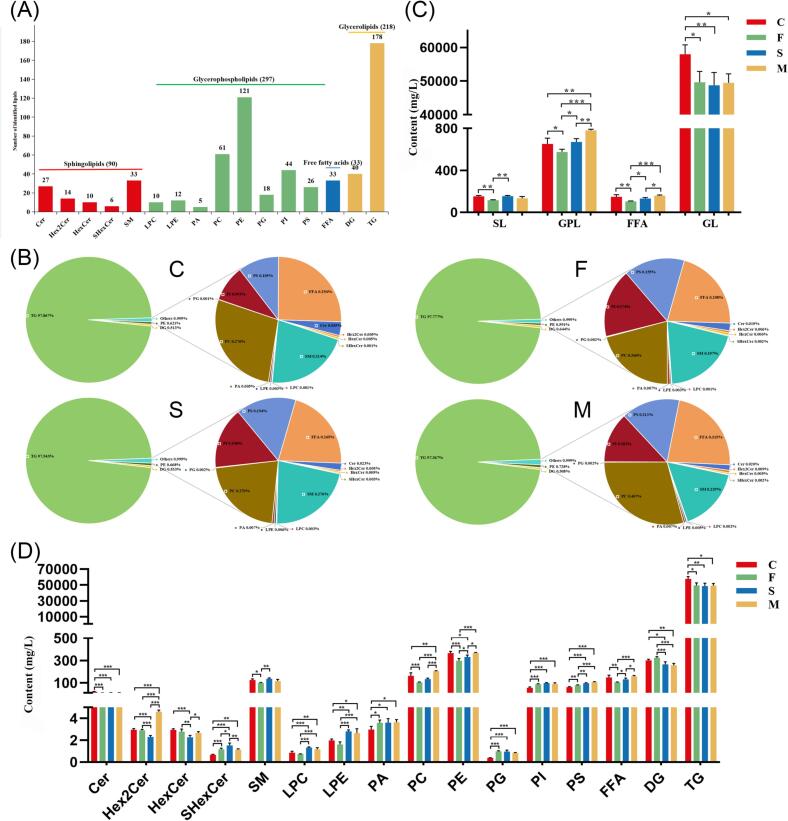
Lipidomics analysis of goat milk from different group. (A) Lipid subclasses identified in goat milk. Number of lipids in each subclass. (B) Distribution of lipid subclasses identified in goat milk. (C) Contents of total SL, GPL, FFA and GL species in goat milk. (D) Contents of each lipid subclass in goat milk. C: control, F: flax seed, S: *Schizochytrium* sp., M: mixture of *Schizochytrium* sp. and flax seed. Values are mean ± SEM (*n* = 4); *p < 0.05, **p < 0.01, ***p < 0.001.

Comprehensive lipid profile of goat milk was elucidated through untargeted lipidomics using UPLC Q-TOF-MS/MS. Drawing on previous experience (Wang et al., 2024; Wu et al., 2020), we systematically annotated all lipid molecular species in goat milk based on retention time, accurate mass, and MS/MS fragmentation information. Deuterated isotope internal standards were employed to complete the quantitative analysis of each lipid molecular species. A total of 638 lipid molecular species were detected in goat milk (Fig. 1 A), including 90 sphingolipids (SL), 297 glycerophospholipids (GPL), 33 free fatty acids (FFA) and 218 glycerolipids (GL). Triacylglycerol (TG) and phosphatidylethanolamine (PE) were the most diverse lipid subclasses, with 178 and 121 molecular species, respectively. Other notable lipid subclasses included phosphatidylcholine (PC, 61), phosphatidylinositol (PI, 44), and diacylglycerol (DG, 40), along with 27 ceramide (Cer), 14 dihexosylceramide (Hex2Cer), 10 hexosylceramide (HexCer), 6 sulfatide (SHexCer), 33 sphingomyelin (SM), 10 lysophosphatidylcholine (LPC), 12 lysophosphatidylethanolamine (LPE), 5 phosphatidic acid (PA), 18 phosphatidylglycerol (PG), 26 phosphatidylserine (PS), and 33 free fatty acid (FFA). In subsequent quantitative analysis, TG content in goat milk from different diets consistently occupied an absolute high content (Fig. 1 B), exceeding 97 %, which aligns with previous studies indicating that TG is the core lipid of fat globules in goat milk (Zhang et al., 2020). The remaining GPL and SL accounted for a very small fraction of milk lipids, primarily located in the MFGM (Silva et al., 2021). Due to the predominance of TG, dietary supplementation had minimal impact on the proportion of each lipid subclass. However, in terms of absolute content (Fig. 1 C), dietary supplementation caused significant differences in lipid composition. The content of GL decreased significantly (*p* < 0.05) due to dietary supplementation. The addition of flax seed alone significantly decreased the main lipid classes, while the addition of the mixture of *Schizochytrium* sp. and flax seed significantly increased (*p* < 0.05) the content of GPL compared to the other groups. Changes in lipid subclass content were observed (Fig. 1 D). A significant reduction (*p* < 0.05) in TG was responsible for the decrease in GL. Unlike the other groups, group F showed an increase in DG. PC and PE, the two most important phospholipids, followed the same trend of change, with group F having the lowest content. This could explain the elevated DG in group F, as DG is required as a substrate for the biosynthesis of PC and PE. However, the addition of flax seed led to a decrease in PC and PE content, thereby reducing the consumption of DG. There were also significant differences in other lipid subclasses among the groups, indicating that the lipid composition in goat milk varies with different dietary supplements.

### 3.3 GL profile of goat milk

**Fig. 2:**
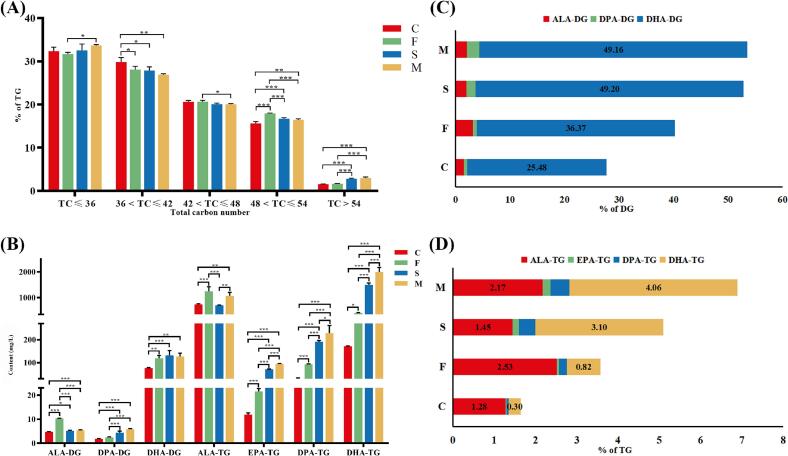
GL analysis of goat milk from different group. (A) Distribution of total carbon number of TG in goat milk (B) Contents of individual ω-3 PUFA GL in goat milk. (C) Percentage of various ω-3 PUFA DG content in goat milk. (D) Percentage of various ω-3 PUFA TG content in goat milk. C: control, F: flax seed, S: *Schizochytrium* sp., M: mixture of *Schizochytrium* sp. and flax seed. Values are mean ± SEM (n = 4); *p < 0.05, **p < 0.01, ***p < 0.001.

As a vital energy substrate, glycerolipids (GL) significantly contribute to milk's nutritional profile and physiological functions, while critically influencing its textural and organoleptic properties. Goat milk GL comprises both TG and DG, with the content of each glyceride molecule detailed in Table S2. TG 8:0_12:0_14:0, TG 10:0_12:0_14:0, TG 8:0_10:0_14:0 and TG 8:0_10:0_12:0 were the most abundant TG, while DG 18:1_22:6, DG 16:0_18:1 and DG 16:0_18:2 were the most abundant DG. Structural analysis revealed that the majority of fatty acids linked to the glycerol backbone of these prevalent TG were medium-chain fatty acids (MCFA) such as C8:0 and C10:0, whereas DG predominantly contained long-chain fatty acids (LFA). Consequently, we calculated the total carbon (TC) number of fatty acids in TG (Fig. 2A). Over 30 % of the TG fatty acids had a TC < 36, which aligns with previous studies indicating that goat milk is richer in MCFA and medium-chain TG compared to cow milk (Gallier et al., 2020). When comparing the results from different diets, this characteristic was most pronounced in group M, which had the highest abundance of TC < 36. Dietary supplementation significantly decreased (*p* < 0.05) the abundance of 36 < TC ≤ 42, while significantly increasing (*p* < 0.05) the abundance of 48 < TC ≤ 54 and TC > 54. This result may be attributed to the increased utilization of C16:0 for synthesizing long-chain ω-3 PUFA, such as EPA and DHA, through elongase and desaturase enzymes. To substantiate this finding, we conducted further analysis of ω-3 PUFA GL. As depicted in Fig. 2B, dietary supplementation led to a significant increase (*p* < 0.05) in the majority of ω-3 PUFA GL compared to group C. Notably, due to the high ALA content in flaxseed, Group F had the highest content of ALA-DG and ALA-TG, and the effect of adding flaxseed alone on ALA was more pronounced than in group M. The GL content of other ω-3 PUFA showed a consistent trend of C < F < S < M. The ω-3 PUFA GL content was most significantly increased by the mixture supplementation of *Schizochytrium* sp. and flaxseed. Although ALA can be used as a biosynthetic precursor for EPA and DHA, the converting efficiency is relatively low (Zhu et al., 2024). Therefore, the addition of flax seed had less effect on ω-3 PUFA GL than the addition of *Schizochytrium* sp. except ALA-DG and ALA-TG. Observing the relative content of PUFA, dietary supplementation made the increase of DHA most evident, with *Schizochytrium* sp. supplementation increasing the relative content of DHA-DG from 25 % to 49 % (Fig. 2C), thus causing the relative content of ω-3 PUFA DG in groups S and M to exceed 50 %. Among TG (Fig. 2D), ALA was most abundant in group C, and the addition of flax seed increased ALA by approximately twofold, while the addition of *Schizochytrium* sp. made DHA-TG the most abundant ω-3 PUFA TG, with a relative content increase of more than tenfold.

### 3.4 GPL profile of goat milk

**Fig. 3:**
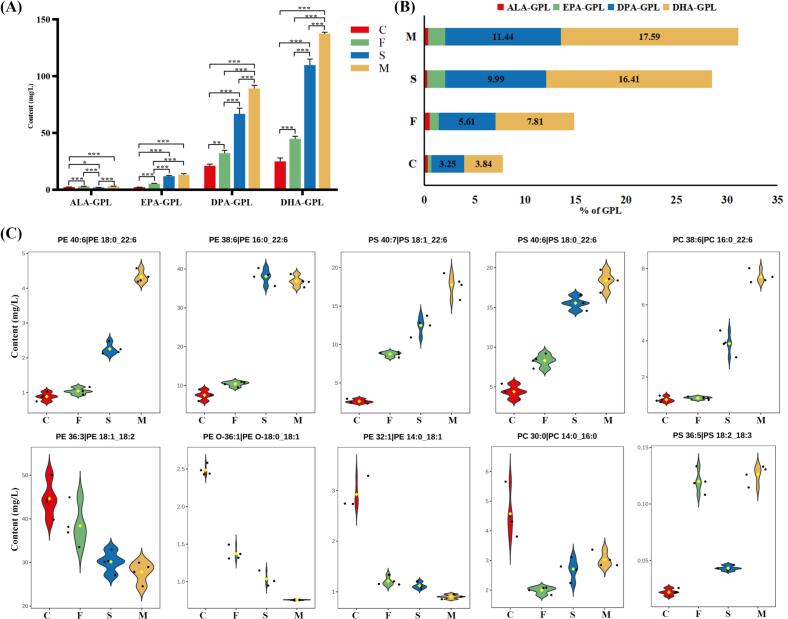
GPL analysis of goat milk from different group. (A) Contents of individual ω-3 PUFA GPL in goat milk. (B) Percentage of various ω-3 PUFA GPL content in goat milk. (C) Some of the differentially varied ω-3 PUFA GPL in goat milk. C: control, F: flax seed, S: *Schizochytrium* sp., M: mixture of *Schizochytrium* sp. and flax seed. Values are mean ± SEM (n = 4); *p < 0.05, **p < 0.01, ***p < 0.001.

As the main component of cell membranes, GPL has a variety of physiological functions and nutritional value. Although GPL constitutes only about 1 % of the total lipids in goat milk, they are the most significant polar lipids and form the MFGM. According to the different polar head groups of GPL, they are divided into different GPL subclasses, such as PC, PE and PS. Our comprehensive analysis of goat milk has revealed the presence of 297 GPL, a number that substantially exceeds the 168 reported by Liu et al. in goat milk (Liu, Yu, Li, Shao & Zhang, 2020). Notably, this finding brings our research closer to the extensive GPL diversity documented by Shang et al., who identified 331 GPL in goat milk (Shang et al., 2022). PE 18:1_18:1, PE 18:1_18:2 and PC 16:0_18:1 were the most abundant GPL molecules in group C (Table S2). We found that these GPL molecules in group C predominantly contained C16:0, C18:1, or C18:2, and their content significantly decreased after dietary supplementation, particularly in group F. Previous studies have shown that C16:0, C18:0, and C18:1 were the major phospholipid fatty acids in animal milk (Wang et al., 2023). This accounts for the significant reduction (*p* < 0.05) in PC and PE content in group F compared to group C. As shown in Fig. 1C, only group M experienced a significant increase (*p* < 0.05) in GPL content relative to group C. However, when analyzing ω-3 PUFA, all three supplemented groups showed a significant increase (*p* < 0.05) in ω-3 PUFA content relative to group C, following the trend of C < F < S < M, except for ALA (Fig. 3A). This trend mirrors that observed in GL ω-3 PUFA content, with group M exerting the most pronounced effect on the increase of ω-3 PUFA. Consequently, PE 16:0_22:6, PE 18:1_22:5, and PS 18:0_22:6 were among the phospholipid molecules with higher content in Group F. The relative contents of ω-3 PUFA GPL were analyzed (Fig. 3B), revealing a composition distinct from that of GL ω-3 PUFA, with a lower proportion of ALA and a predominance of DPA and DHA. Compared to group C, the DPA-GPL content in group M increased from 3.25 % to 11.44 %, and the DHA content increased from 3.84 % to 17.59 %. Further analysis of different GPL molecules indicated that the addition of *Schizochytrium* sp. significantly increased DHA-GPL molecules (Fig. 3C), such as PE 18:0_22:6 and PS 18:1_22:6. Previous studies have shown that DHA supplementation can promote lipid droplet accumulation, thereby altering lipid metabolism in mammary cells (Wu et al., 2023). Therefore, the addition of DHA-rich *Schizochytrium* sp. caused these positive changes in goat milk. The addition of flax seed more prominently increased ALA-GPL, such as PS 18:2_18:3. Additionally, some plasmalogens, like PE P-18:1–22:6 and PE P-18:0–22:6, were significantly increased after dietary supplementation. Plasmalogens are recognized for their significant nutritional functions, including the amelioration of neurodegenerative diseases (Wang et al., 2021). The enrichment of these ω-3 PUFA GPL in goat milk is undoubtedly highly beneficial for its nutritional value. ω-3 PUFA GPL are known for their higher bioavailability (Wang et al., 2024), typically sourced from marine organisms, dietary supplementation now positions goat milk as an important source of ω-3 PUFA GPL. Moreover, the increase of all ω-3 PUFA lipids caused by dietary supplements makes goat milk more suitable for infant formula.

### 3.5 Multivariate statistical analysis of goat milk

**Fig. 4:**
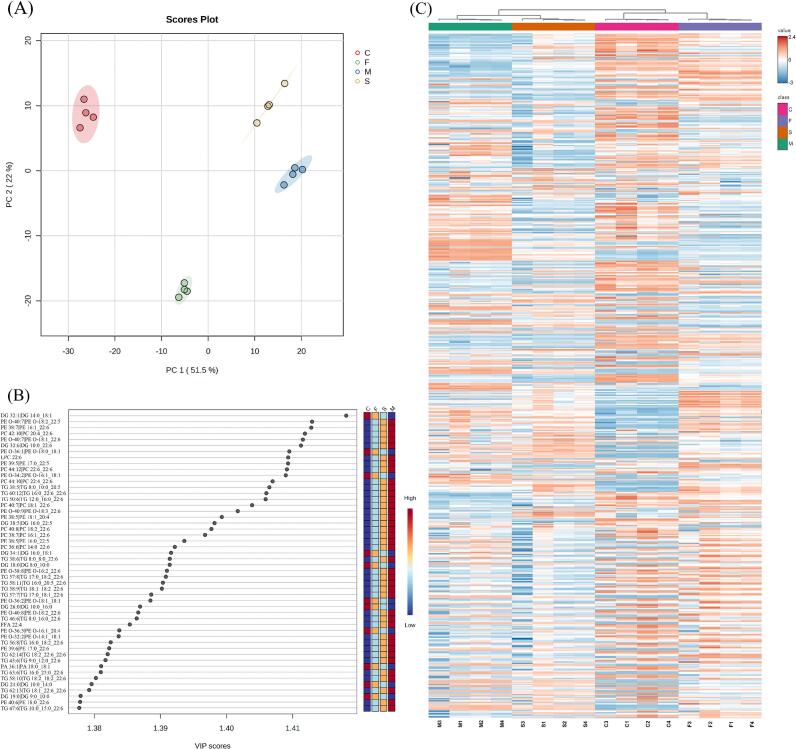
Multivariate statistical analysis of goat milk from different group. (A) PCA score plot of all lipid molecular species in goat milk. (B) VIP scores of individual lipids in PLS-DA. (C) Heat map analysis of all lipid molecular species in goat milk. C: control, F: flax seed, S: *Schizochytrium* sp., M: mixture of *Schizochytrium* sp. and flax seed.

We employed multivariate statistical analysis to differentiate the lipid profiles among the four groups and assess the impact of various dietary supplements on goat milk lipids. The PCA score plot (Fig. 4A) revealed that the groups formed four distinct clusters based on principal component 1 (PC1) and principal component 2 (PC2), signifying clear differences in lipid content. PC1, contributing 51.5 %, positioned Groups S and M on the right axis, indicating that the addition of *Schizochytrium* sp. was a primary factor influencing the lipid composition of goat milk. PC2, contributing 22 %, placed group F below the axis, suggesting that the addition of flax seed was a secondary factor affecting the lipidomics of goat milk. The variable importance in the projection (VIP) values (Fig. 4B) was used to identify lipids closely associated with the classification in the PLS-DA, with more than half of the lipid molecules exhibiting VIP values >1. The top 50 lipids, as shown in Fig. 4B, were highlighted, with DG 14:0_18:1 having the highest VIP value, followed by PE O-18:2_22:5, PE 16:1_22:6, and PC 20:4_22:6. The majority of these lipid molecules contained ω-3 PUFA, indicating the effectiveness of dietary supplementation in enhancing the ω-3 PUFA content in goat milk. A heat map with unsupervised hierarchical clustering was then used to visualize the variation in lipid content among the groups (Fig. 4C). Two main clusters were separated: group S and M on the left, and group C and F on the right, implying that the lipid profile differences between F and C were less pronounced compared to other groups. Additionally, the lipid composition of group M was more similar to that of group S, suggesting that *Schizochytrium* sp. supplementation may have had a more significant impact on goat milk lipids than flax seed supplementation. This finding is consistent with the PCA analysis results.

**Fig. 5:**
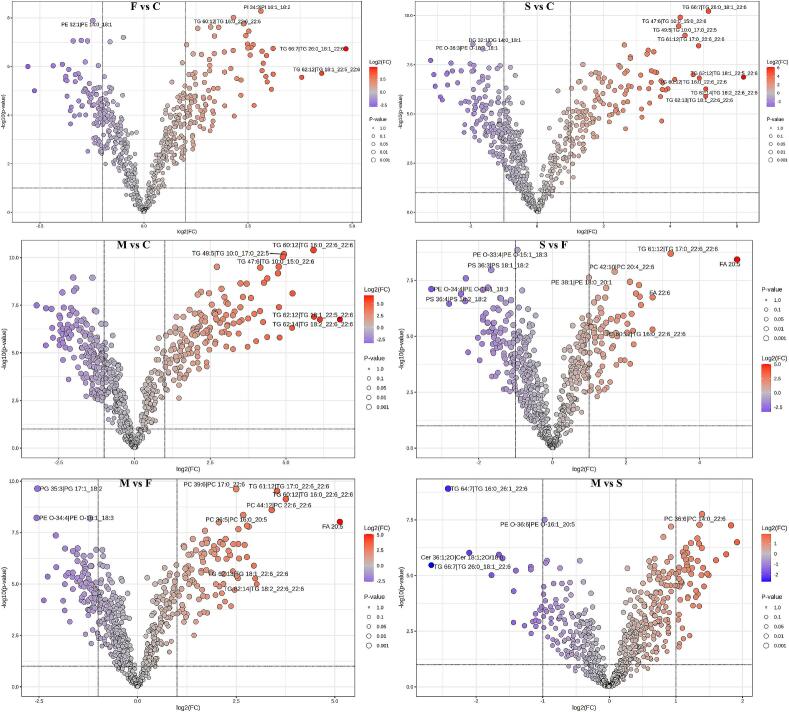
Volcano plot of lipid composition in different groups of goat milk. C: control, F: flax seed, S: *Schizochytrium* sp., M: mixture of *Schizochytrium* sp. and flax seed.

Subsequently, we conducted a volcano plot analysis to explore the differences among these groups in greater detail. The volcano plot (Fig. 5), with a threshold of VIP value >1, *p* < 0.05, and fold change (FC) > 1 (up-regulated lipids) or < 1 (down-regulated lipids), compared the dietary supplementation groups with group C. A higher number of differential lipids were identified between group M vs C, while the least number of differential lipids were found between group F vs C. These results indicate that the combined supplementation of *Schizochytrium* sp. and flax seed significantly affected the lipid content in goat milk, particularly contributing to the up-regulation of lipids containing ω-3 PUFA. Pairwise comparisons were also used to explore differential lipids among groups S, F, and M. These comparisons revealed a high number of differential lipids in group S vs F and Group M vs F, with 20 down-regulated and 53 up-regulated lipids identified between group M and S. Compared with group F, some ALA-containing lipids were down-regulated in groups S and M. Collectively, these results demonstrate that *Schizochytrium* sp. dietary supplementation significantly impacts the lipid content of goat milk and accounts for a substantial proportion of the effects observed in mixed dietary supplement interventions on goat milk lipids.

## 4 Conclusion

In conclusion, this study utilized *Schizochytrium* sp., flax seed, and their mixture as dietary supplements to obtain goat milk. We comprehensively evaluated and compared the lipid molecular species composition across four groups of goat milk using untargeted lipidomics with UPLC Q-TOF-MS/MS technology. A total of 638 lipid molecular species across 16 lipid subclasses were detected, with TG constituting over 97 % of the total lipids. Dietary supplementation significantly reduced GL content while elevating ω-3 PUFA levels across both GL and GPL. The mixed supplementation exhibited the most pronounced effect, increasing DHA-TG and DHA-GPL by >10 fold and > 4 fold, respectively, with ω-3 PUFA DG exceeding 50 % in group M The addition of flax seed was particularly effective in increasing ALA-containing lipids in goat milk, whereas *Schizochytrium* sp. and the mixed diet enriched DHA and DPA, demonstrating distinct metabolic incorporation pathways for different ω-3 PUFA sources. Finally, multivariate statistical analysis confirmed ω-3 PUFA as key differential metabolites, aligning with the enhanced bioavailability and health benefits of ω-3 PUFA-enriched goat milk, particularly for infant formula applications. Collectively, these findings provide valuable insights into the production of ω-3 PUFA-enriched goat milk and offer a robust theoretical foundation for its nutritional evaluation, identification, and application. Our study highlights the potential of dietary supplementation with *Schizochytrium* sp. and flax seed to enhance the nutritional quality of goat milk, making it a promising functional dairy product for infant formula and other nutritional applications.

## CRediT authorship contribution statement

**Jie Wang:** Writing – original draft, Software, Methodology, Investigation, Data curation, Conceptualization. **Shiqian Ran:** Writing – original draft, Software, Data curation, Conceptualization. **Xin Lv:** Formal analysis, Data curation. **Dan Wang:** Writing – review & editing, Conceptualization. **Hong Chen:** Software, Methodology. **Fang Wei:** Writing – review & editing, Validation, Supervision, Investigation.

## Fundings

This work is supported by the 10.13039/501100001809National Natural Science Foundation of China (Grant No. 32472446). We also gratefully acknowledge the support of the National Key Research and Development Pro-gram Key Special Project (Grant No. 2024YFF1106101 and 2021YFD1600103), Technology Innovation Project of Hubei Province (Grant No. 2023DJC150), the innovation group project of Hubei Province (2023AFA042), and Agricultural Science and Technology Innovation Project of Chinese Academy of Agricultural Sciences (CAASASTIP-2013-OCRI).

## Declaration of competing interest

The authors declare that they have no known competing financial interests or personal relationships that could have appeared to influence the work reported in this paper.

## Data Availability

No data was used for the research described in the article.

## Appendix A Supplementary data

Supplementary material Image 1
