# Supplementary material for: Comprehensive lipidomics analysis reveals the effects in goat milk from different ω-3 PUFA dietary supplementation by UPLC Q-TOF-MS/MS
Source: Food Chem X. 2025 May 23;28:102581. doi: 10.1016/j.fochx.2025.102581 (PMC12155763; doi:10.1016/j.fochx.2025.102581)
Supplement: Supplementary file 1 — Supplementary material [file mmc1.docx]

**Comprehensive lipidomics analysis reveals the effects in goat milk from different ω-3 PUFA dietary supplementation by UPLC Q-TOF-MS/MS**

Jie Wang^a#^, Shiqian Ran^a,c#^, Xin Lv^a^, Dan Wang^a^, Hong Chen^a,d^, Fang Wei^a,b*^

^a^ Key Laboratory of Oilseeds Processing of Ministry of Agriculture, Hubei Key Laboratory of Lipid Chemistry and Nutrition, Oil Crops Research Institute of the Chinese Academy of Agricultural Sciences, Wuhan, Hubei 430062, P.R. China

^b^ Hubei Hongshan Laboratory, Wuhan, Hubei, 430070, PR China

^c^ Zunyi Rural Development Service Center, Zunyi, Guizhou 563000, P.R. China

^d^ Hubei Key Laboratory of Animal Nutrition and Feed Science, Wuhan Polytechnic University, Wuhan, People's Republic of China, 430023

^#^These authors contributed equally to this work

*To whom correspondence should be addressed. Tel.: +86-27-86711669; Fax: +86-27-86822291. E-mail address: Fang Wei: [willasa@163.com](mailto:willasa@163.com)


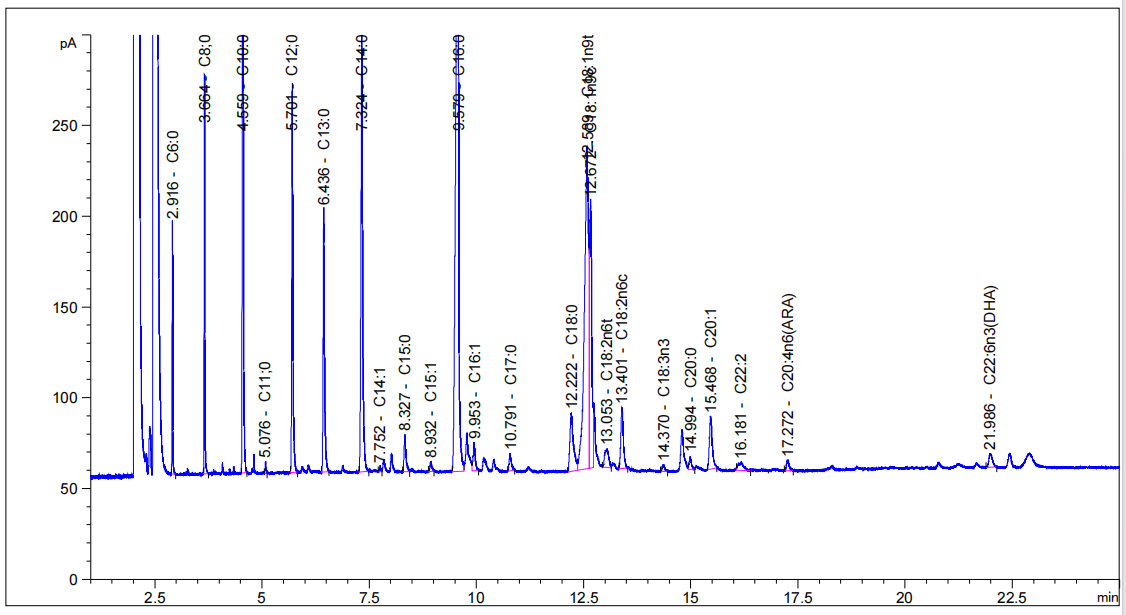
Figure S1. The GC chromatogram of goat milk.


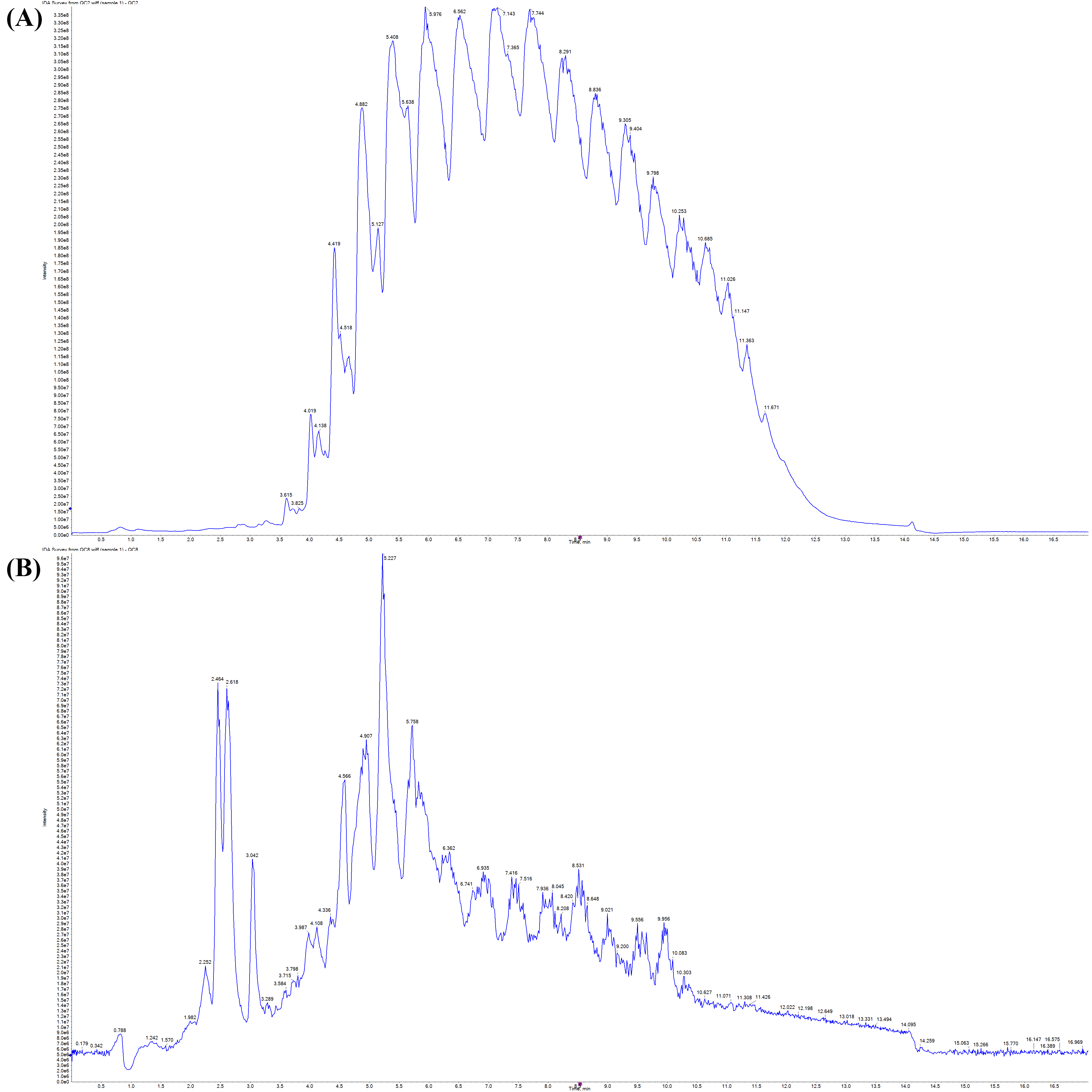


Figure S2. Total ion chromatogram (TIC) of the lipid profile in goat milk acquired using UPLC-Q-TOF-MS/MS, (A) positive, (B) negative .

Table S1 Ingredients of experimental diets and FA composition of flax seed and Schizochytrium sp. (%)

| Composition（%） | C | F | S | M |
| --- | --- | --- | --- | --- |
| Corn | 57% | 57% | 57% | 57% |
| Wheat bran | 12% | 12% | 12% | 12% |
| Soybean meal | 25% | 25% | 25% | 25% |
| Sodium bicarbonate | 1% | 1% | 1% | 1% |
| Dicalcium phosphate | 1% | 1% | 1% | 1% |
| Salt | 1% | 1% | 1% | 1% |
| Premix | 3% | 3% | 3% | 3% |
| Flax seed（mg） | - | 50 | - | 50 |
| Schizochytrium sp.(mg) | - | - | 50 | 50 |
|  | | | | |
| FFAtty acid | Schizochytrium sp. | | flax seed | |
| C14:0 | 7.81±0.018 | | nd | |
| C16:0 | 26.15±0.033 | | 5.97±0.24 | |
| C16:1 | 4.98±0.017 | | nd | |
| C18:0 | nd | | 4.55±0.11 | |
| C18:1 | 6.29±0.044 | | 20.18±0.65 | |
| C18:2 | nd | | 13.88±0.54 | |
| C18:3 | nd | | 55.43±0.47 | |
| C20:4 | 0.58±0.029 | | nd | |
| C22:0 | 0.51±0.036 | | nd | |
| C22:5 | 1.79±0.026 | | nd | |
| C24:0 | 14.48±0.052 | | nd | |
| C22:6 | 37.41±0.137 | | nd | |

Table S2. Lipid contens (μg/mL) in goat milk.

| lipid Name | C1 | C2 | C3 | C4 | F1 | F2 | F3 | F4 | S1 | S2 | S3 | S4 | M1 | M2 | M3 | M4 |
| --- | --- | --- | --- | --- | --- | --- | --- | --- | --- | --- | --- | --- | --- | --- | --- | --- |
| Cer 26:1;2O\|Cer 16:1;2O/10:0 | 0.01 | 0.01 | 0.01 | 0.01 | 0.01 | 0.01 | 0.01 | 0.01 | 0.01 | 0.01 | 0.01 | 0.01 | 0.01 | 0.01 | 0.01 | 0.01 |
| Cer 28:0;2O\|Cer 18:0;2O/10:0 | 0.02 | 0.02 | 0.02 | 0.02 | 0.01 | 0.01 | 0.01 | 0.01 | 0.02 | 0.02 | 0.01 | 0.01 | 0.01 | 0.01 | 0.01 | 0.01 |
| Cer 28:1;2O\|Cer 18:1;2O/10:0 | 0.04 | 0.04 | 0.04 | 0.04 | 0.02 | 0.03 | 0.03 | 0.03 | 0.03 | 0.03 | 0.02 | 0.02 | 0.02 | 0.02 | 0.01 | 0.02 |
| Cer 28:2;2O\|Cer 12:2;2O/16:0 | 0.01 | 0.01 | 0.01 | 0.01 | 0.01 | 0.01 | 0.01 | 0.01 | 0.01 | 0.01 | 0.01 | 0.01 | 0.01 | 0.01 | 0.01 | 0.01 |
| Cer 30:0;2O\|Cer 12:0;2O/18:0 | 0.05 | 0.04 | 0.05 | 0.05 | 0.01 | 0.02 | 0.01 | 0.01 | 0.03 | 0.03 | 0.02 | 0.02 | 0.01 | 0.01 | 0.01 | 0.01 |
| Cer 32:1;2O\|Cer 18:1;2O/14:0 | 0.35 | 0.29 | 0.25 | 0.29 | 0.12 | 0.12 | 0.13 | 0.12 | 0.17 | 0.16 | 0.11 | 0.14 | 0.10 | 0.08 | 0.07 | 0.08 |
| Cer 33:1;2O\|Cer 17:1;2O/16:0 | 0.12 | 0.11 | 0.14 | 0.12 | 0.06 | 0.05 | 0.07 | 0.06 | 0.06 | 0.05 | 0.03 | 0.05 | 0.04 | 0.03 | 0.04 | 0.04 |
| Cer 34:0;2O\|Cer 15:0;2O/19:0 | 0.20 | 0.19 | 0.16 | 0.18 | 0.19 | 0.18 | 0.22 | 0.20 | 0.17 | 0.19 | 0.10 | 0.14 | 0.10 | 0.09 | 0.08 | 0.09 |
| Cer 34:1;2O\|Cer 18:1;2O/16:0 | 2.14 | 2.44 | 2.45 | 2.29 | 0.71 | 0.76 | 0.95 | 0.80 | 0.90 | 0.96 | 0.69 | 0.82 | 0.59 | 0.49 | 0.51 | 0.53 |
| Cer 34:2;2O\|Cer 18:1;2O/16:1 | 0.17 | 0.18 | 0.23 | 0.19 | 0.18 | 0.14 | 0.14 | 0.15 | 0.18 | 0.12 | 0.10 | 0.13 | 0.17 | 0.19 | 0.16 | 0.17 |
| Cer 35:0;2O\|Cer 12:0;2O/23:0 | 0.05 | 0.06 | 0.07 | 0.06 | 0.05 | 0.05 | 0.06 | 0.05 | 0.06 | 0.06 | 0.05 | 0.06 | 0.03 | 0.03 | 0.02 | 0.03 |
| Cer 35:1;2O\|Cer 19:1;2O/16:0 | 0.10 | 0.12 | 0.11 | 0.11 | 0.06 | 0.05 | 0.07 | 0.06 | 0.14 | 0.12 | 0.08 | 0.11 | 0.08 | 0.06 | 0.05 | 0.06 |
| Cer 36:1;2O\|Cer 18:1;2O/18:0 | 0.28 | 0.35 | 0.46 | 0.36 | 0.06 | 0.05 | 0.06 | 0.06 | 0.35 | 0.39 | 0.45 | 0.40 | 0.10 | 0.09 | 0.07 | 0.09 |
| Cer 36:2;2O\|Cer 18:1;2O/18:1 | 0.20 | 0.19 | 0.23 | 0.20 | 0.13 | 0.14 | 0.13 | 0.14 | 0.26 | 0.28 | 0.16 | 0.22 | 0.10 | 0.09 | 0.09 | 0.09 |
| Cer 39:1;2O\|Cer 18:1;2O/21:0 | 0.22 | 0.26 | 0.34 | 0.28 | 0.10 | 0.09 | 0.08 | 0.09 | 0.21 | 0.21 | 0.27 | 0.23 | 0.19 | 0.14 | 0.12 | 0.15 |
| Cer 40:1;2O\|Cer 18:1;2O/22:0 | 1.76 | 2.45 | 2.06 | 2.10 | 1.28 | 1.11 | 1.28 | 1.22 | 1.65 | 1.47 | 1.67 | 1.59 | 1.66 | 1.17 | 1.31 | 1.38 |
| Cer 40:2;2O\|Cer 18:1;2O/22:1 | 0.65 | 0.71 | 0.91 | 0.76 | 0.12 | 0.10 | 0.10 | 0.11 | 0.14 | 0.13 | 0.12 | 0.13 | 0.16 | 0.13 | 0.15 | 0.15 |
| Cer 41:1;2O\|Cer 18:1;2O/23:0 | 3.12 | 4.07 | 5.04 | 4.09 | 1.93 | 1.79 | 1.86 | 1.86 | 2.40 | 2.29 | 2.64 | 2.44 | 2.91 | 1.97 | 1.71 | 2.20 |
| Cer 41:2;3O\|Cer 21:2;2O/20:0;(2OH) | 0.02 | 0.02 | 0.02 | 0.02 | 0.04 | 0.04 | 0.05 | 0.05 | 0.04 | 0.05 | 0.03 | 0.04 | 0.03 | 0.03 | 0.03 | 0.03 |
| Cer 42:1;2O\|Cer 18:1;2O/24:0 | 4.50 | 5.79 | 5.96 | 5.43 | 3.39 | 3.17 | 3.15 | 3.23 | 2.74 | 2.78 | 2.89 | 2.80 | 3.96 | 2.65 | 2.25 | 2.95 |
| Cer 42:2;2O\|Cer 18:1;2O/24:1 | 1.99 | 2.20 | 2.84 | 2.35 | 0.74 | 0.61 | 0.57 | 0.64 | 1.17 | 1.19 | 1.33 | 1.23 | 1.31 | 1.08 | 0.92 | 1.10 |
| Cer 42:2;3O\|Cer 21:1;2O/21:1;O | 0.02 | 0.02 | 0.02 | 0.02 | 0.04 | 0.03 | 0.04 | 0.03 | 0.06 | 0.07 | 0.05 | 0.06 | 0.03 | 0.02 | 0.03 | 0.03 |
| Cer 43:1;2O\|Cer 18:1;2O/25:0 | 1.01 | 1.29 | 1.71 | 1.34 | 0.65 | 0.55 | 0.64 | 0.61 | 0.42 | 0.45 | 0.55 | 0.47 | 0.76 | 0.69 | 0.81 | 0.75 |
| Cer 44:2;3O\|Cer 22:0;2O/22:2;(2OH) | 0.03 | 0.03 | 0.02 | 0.03 | 0.06 | 0.06 | 0.05 | 0.06 | 0.09 | 0.08 | 0.05 | 0.07 | 0.05 | 0.04 | 0.05 | 0.05 |
| Cer 45:3;4O\|Cer 27:1;3O(FFA 18:1) | 0.22 | 0.17 | 0.21 | 0.20 | 0.10 | 0.09 | 0.11 | 0.10 | 0.08 | 0.10 | 0.07 | 0.08 | 0.07 | 0.07 | 0.08 | 0.07 |
| Cer 58:3;4O\|Cer 44:1;3O(FFA 14:1) | 0.01 | 0.01 | 0.01 | 0.01 | 0.01 | 0.02 | 0.02 | 0.02 | 0.01 | 0.01 | 0.00 | 0.01 | 0.01 | 0.00 | 0.00 | 0.01 |
| Cer 59:2;4O\|Cer 41:1;3O(FFA 18:0) | 0.01 | 0.01 | 0.01 | 0.01 | 0.00 | 0.01 | 0.01 | 0.01 | 0.01 | 0.01 | 0.01 | 0.01 | 0.00 | 0.00 | 0.00 | 0.00 |
| DG 18:0\|DG 8:0_10:0 | 4.09 | 3.04 | 3.25 | 3.47 | 2.05 | 1.88 | 1.73 | 1.89 | 1.96 | 1.70 | 1.48 | 1.71 | 0.96 | 0.73 | 0.62 | 0.77 |
| DG 19:0\|DG 9:0_10:0 | 0.75 | 0.56 | 0.66 | 0.66 | 0.57 | 0.53 | 0.60 | 0.57 | 0.39 | 0.44 | 0.40 | 0.41 | 0.24 | 0.25 | 0.27 | 0.25 |
| DG 20:0\|DG 10:0_10:0 | 13.39 | 11.60 | 11.83 | 12.29 | 8.80 | 8.20 | 8.35 | 8.44 | 8.47 | 8.37 | 7.98 | 8.28 | 3.17 | 3.15 | 2.69 | 3.01 |
| DG 21:0\|DG 10:0_11:0 | 0.83 | 0.78 | 0.89 | 0.83 | 0.97 | 0.88 | 1.11 | 0.98 | 0.68 | 0.68 | 0.86 | 0.74 | 0.24 | 0.34 | 0.44 | 0.34 |
| DG 22:0\|DG 10:0_12:0 | 3.97 | 3.69 | 4.58 | 4.08 | 2.37 | 2.32 | 2.43 | 2.37 | 2.47 | 2.81 | 3.18 | 2.82 | 1.05 | 1.24 | 1.28 | 1.19 |
| DG 24:0\|DG 10:0_14:0 | 10.78 | 10.89 | 12.88 | 11.51 | 6.74 | 7.43 | 9.28 | 7.83 | 7.06 | 5.30 | 4.64 | 5.68 | 2.68 | 3.01 | 2.65 | 2.78 |
| DG 26:0\|DG 10:0_16:0 | 8.62 | 8.22 | 10.21 | 9.02 | 5.21 | 5.83 | 7.07 | 6.04 | 4.01 | 6.07 | 6.09 | 5.37 | 3.36 | 3.33 | 2.94 | 3.21 |
| DG 26:1\|DG 8:0_18:1 | 9.88 | 9.68 | 8.12 | 9.23 | 7.72 | 8.62 | 10.77 | 9.05 | 6.28 | 5.13 | 5.34 | 5.60 | 3.81 | 4.50 | 4.64 | 4.32 |
| DG 26:2\|DG 8:0_18:2 | 8.00 | 7.62 | 8.83 | 8.15 | 11.08 | 12.25 | 14.59 | 12.66 | 6.27 | 5.33 | 6.15 | 5.93 | 5.55 | 6.74 | 8.02 | 6.77 |
| DG 26:3\|DG 8:0_18:3 | 0.90 | 0.84 | 0.72 | 0.82 | 1.69 | 1.62 | 1.41 | 1.57 | 0.83 | 0.84 | 0.66 | 0.78 | 0.69 | 0.72 | 0.65 | 0.69 |
| DG 27:2\|DG 9:0_18:2 | 0.28 | 0.31 | 0.35 | 0.31 | 0.69 | 0.69 | 0.59 | 0.65 | 0.36 | 0.36 | 0.42 | 0.38 | 0.25 | 0.28 | 0.34 | 0.29 |
| DG 28:0\|DG 12:0_16:0 | 5.35 | 5.49 | 6.06 | 5.63 | 2.81 | 2.48 | 2.17 | 2.48 | 2.89 | 2.81 | 2.71 | 2.80 | 1.58 | 1.33 | 1.09 | 1.33 |
| DG 28:1\|DG 10:0_18:1 | 11.79 | 10.61 | 12.73 | 11.72 | 11.41 | 11.78 | 13.25 | 12.15 | 6.80 | 8.67 | 8.24 | 7.89 | 6.43 | 6.12 | 5.83 | 6.13 |
| DG 28:2\|DG 10:0_18:2 | 16.84 | 15.99 | 19.51 | 17.45 | 23.43 | 24.08 | 27.94 | 25.16 | 12.59 | 11.06 | 9.23 | 10.97 | 13.23 | 14.43 | 14.06 | 13.91 |
| DG 28:3\|DG 10:0_18:3 | 2.07 | 2.03 | 1.91 | 2.00 | 4.37 | 4.69 | 4.01 | 4.36 | 1.74 | 1.60 | 1.38 | 1.57 | 1.79 | 1.95 | 1.58 | 1.78 |
| DG 28:4\|DG 8:0_20:4 | 0.68 | 0.81 | 0.62 | 0.70 | 2.10 | 1.89 | 1.24 | 1.74 | 1.62 | 1.57 | 1.19 | 1.46 | 3.10 | 3.65 | 2.26 | 3.00 |
| DG 30:0\|DG 14:0_16:0 | 8.67 | 8.21 | 10.27 | 9.05 | 3.06 | 2.82 | 2.55 | 2.81 | 3.02 | 3.62 | 3.98 | 3.54 | 1.90 | 1.74 | 1.48 | 1.71 |
| DG 30:1\|DG 12:0_18:1 | 5.54 | 6.04 | 7.19 | 6.25 | 4.76 | 4.32 | 5.05 | 4.71 | 3.49 | 3.18 | 2.83 | 3.17 | 2.48 | 2.14 | 2.36 | 2.32 |
| DG 30:2\|DG 12:0_18:2 | 3.45 | 3.24 | 2.95 | 3.21 | 3.96 | 3.99 | 4.82 | 4.26 | 1.91 | 2.19 | 1.73 | 1.94 | 2.46 | 2.38 | 2.41 | 2.41 |
| DG 30:3\|DG 12:0_18:3 | 0.81 | 0.79 | 0.80 | 0.80 | 1.36 | 1.45 | 1.77 | 1.53 | 0.60 | 0.78 | 0.61 | 0.66 | 0.75 | 0.72 | 0.70 | 0.72 |
| DG 30:4\|DG 10:0_20:4 | 0.77 | 0.82 | 0.69 | 0.76 | 0.98 | 0.99 | 0.98 | 0.98 | 1.10 | 1.02 | 0.90 | 1.01 | 1.11 | 1.23 | 1.55 | 1.30 |
| DG 30:6\|DG 8:0_22:6 | 2.36 | 2.53 | 2.99 | 2.62 | 9.76 | 9.04 | 5.19 | 7.99 | 5.11 | 5.32 | 5.09 | 5.17 | 12.43 | 15.74 | 13.05 | 13.74 |
| DG 32:0\|DG 16:0_16:0 | 10.95 | 10.02 | 10.66 | 10.55 | 4.05 | 3.80 | 4.15 | 4.00 | 4.85 | 4.96 | 5.15 | 4.98 | 3.26 | 2.77 | 2.38 | 2.80 |
| DG 32:1\|DG 14:0_18:1 | 11.50 | 10.84 | 10.66 | 11.00 | 6.26 | 5.81 | 6.67 | 6.24 | 4.62 | 5.42 | 5.56 | 5.19 | 3.50 | 3.46 | 3.04 | 3.33 |
| DG 32:2\|DG 14:0_18:2 | 7.21 | 7.58 | 9.30 | 8.03 | 5.73 | 5.25 | 5.41 | 5.46 | 2.65 | 3.13 | 2.48 | 2.75 | 3.84 | 3.68 | 3.04 | 3.52 |
| DG 32:3\|DG 14:0_18:3 | 0.80 | 0.77 | 0.78 | 0.78 | 1.06 | 1.08 | 1.37 | 1.17 | 0.48 | 0.48 | 0.61 | 0.52 | 0.68 | 0.59 | 0.72 | 0.66 |
| DG 32:6\|DG 10:0_22:6 | 1.88 | 1.53 | 1.49 | 1.64 | 3.04 | 2.94 | 3.00 | 2.99 | 5.72 | 5.47 | 5.80 | 5.67 | 8.96 | 9.54 | 8.25 | 8.91 |
| DG 34:0\|DG 16:0_18:0 | 2.46 | 2.70 | 3.07 | 2.74 | 1.12 | 0.99 | 1.01 | 1.04 | 0.88 | 1.04 | 1.11 | 1.01 | 0.74 | 0.60 | 0.50 | 0.61 |
| DG 34:1\|DG 16:0_18:1 | 20.20 | 22.01 | 22.67 | 21.61 | 14.69 | 13.92 | 14.68 | 14.42 | 12.46 | 11.24 | 14.24 | 12.66 | 11.08 | 8.63 | 8.16 | 9.29 |
| DG 34:2\|DG 16:0_18:2 | 17.75 | 19.31 | 19.88 | 18.97 | 16.12 | 14.76 | 16.36 | 15.73 | 9.78 | 10.47 | 8.57 | 9.60 | 13.66 | 12.26 | 12.45 | 12.79 |
| DG 34:3\|DG 16:1_18:2 | 1.81 | 1.89 | 1.74 | 1.81 | 2.75 | 2.44 | 3.19 | 2.79 | 0.90 | 1.21 | 1.20 | 1.10 | 1.83 | 1.56 | 1.97 | 1.79 |
| DG 36:1\|DG 18:0_18:1 | 3.32 | 3.46 | 4.07 | 3.62 | 1.93 | 1.70 | 1.46 | 1.69 | 1.23 | 1.01 | 1.05 | 1.10 | 1.01 | 0.79 | 1.00 | 0.93 |
| DG 36:2\|DG 18:1_18:1 | 11.10 | 12.33 | 11.36 | 11.59 | 12.47 | 11.88 | 11.43 | 11.92 | 6.49 | 6.85 | 5.45 | 6.26 | 7.07 | 5.79 | 7.07 | 6.64 |
| DG 36:3\|DG 18:1_18:2 | 9.50 | 10.11 | 12.04 | 10.54 | 15.96 | 14.33 | 12.86 | 14.36 | 6.31 | 6.82 | 5.71 | 6.27 | 10.06 | 8.60 | 7.38 | 8.68 |
| DG 36:4\|DG 18:2_18:2 | 4.11 | 4.97 | 2.79 | 3.95 | 13.97 | 12.63 | 8.21 | 11.59 | 3.59 | 3.89 | 3.86 | 3.78 | 14.17 | 14.69 | 12.20 | 13.68 |
| DG 36:5\|DG 18:2_18:3 | 0.27 | 0.25 | 0.30 | 0.27 | 1.58 | 1.53 | 1.77 | 1.63 | 1.56 | 1.73 | 1.57 | 1.62 | 1.63 | 1.52 | 1.36 | 1.50 |
| DG 36:6\|DG 14:0_22:6 | 15.51 | 17.04 | 17.55 | 16.69 | 17.85 | 15.93 | 15.98 | 16.56 | 17.93 | 19.57 | 16.78 | 18.08 | 21.86 | 21.49 | 12.58 | 18.64 |
| DG 38:5\|DG 16:0_22:5 | 1.76 | 1.88 | 1.88 | 1.84 | 2.35 | 2.19 | 2.77 | 2.43 | 3.68 | 4.38 | 5.19 | 4.41 | 6.02 | 5.51 | 6.03 | 5.86 |
| DG 38:6\|DG 16:0_22:6 | 2.86 | 3.21 | 3.93 | 3.33 | 3.63 | 3.76 | 4.81 | 4.07 | 6.87 | 8.53 | 7.90 | 7.75 | 12.06 | 10.75 | 9.51 | 10.77 |
| DG 40:7\|DG 18:1_22:6 | 52.39 | 52.85 | 52.87 | 52.70 | 98.61 | 88.92 | 73.11 | 86.75 | 105.26 | 112.39 | 65.72 | 94.40 | 77.45 | 83.08 | 63.16 | 74.55 |
| FFA 16:1 | 8.52 | 7.47 | 8.09 | 9.34 | 3.73 | 3.80 | 4.47 | 4.01 | 4.23 | 3.99 | 4.02 | 4.08 | 5.33 | 5.00 | 4.65 | 5.01 |
| FFA 16:2 | 0.42 | 0.36 | 0.32 | 0.43 | 0.35 | 0.36 | 0.27 | 0.32 | 0.26 | 0.28 | 0.27 | 0.27 | 0.68 | 0.65 | 0.66 | 0.66 |
| FFA 17:0 | 1.99 | 1.68 | 1.99 | 2.19 | 1.37 | 1.21 | 1.32 | 1.30 | 2.27 | 1.91 | 1.79 | 1.99 | 2.06 | 1.89 | 2.47 | 2.13 |
| FFA 17:1 | 0.87 | 0.82 | 1.00 | 1.04 | 0.29 | 0.31 | 0.26 | 0.29 | 0.54 | 0.52 | 0.58 | 0.55 | 0.44 | 0.44 | 0.40 | 0.43 |
| FFA 18:0 | 48.91 | 31.88 | 32.84 | 44.07 | 49.21 | 41.07 | 46.09 | 45.43 | 51.71 | 41.19 | 37.97 | 43.45 | 46.25 | 41.29 | 42.73 | 43.47 |
| FFA 18:1 | 58.93 | 53.87 | 43.22 | 60.50 | 27.13 | 26.01 | 24.87 | 25.97 | 36.47 | 34.44 | 30.56 | 33.73 | 41.56 | 38.24 | 49.05 | 42.78 |
| FFA 18:2 | 33.42 | 31.73 | 29.22 | 36.59 | 19.09 | 19.23 | 18.73 | 19.01 | 19.70 | 18.89 | 18.99 | 19.19 | 31.34 | 29.95 | 34.02 | 31.71 |
| FFA 18:3 | 2.77 | 2.28 | 2.92 | 3.09 | 2.42 | 2.46 | 2.32 | 2.40 | 1.50 | 1.49 | 1.30 | 1.43 | 2.96 | 2.77 | 3.02 | 2.91 |
| FFA 19:0 | 0.34 | 0.17 | 0.17 | 0.27 | 0.23 | 0.22 | 0.26 | 0.24 | 0.35 | 0.30 | 0.25 | 0.30 | 0.26 | 0.25 | 0.23 | 0.24 |
| FFA 19:1 | 0.62 | 0.58 | 0.74 | 0.75 | 0.19 | 0.19 | 0.16 | 0.18 | 0.33 | 0.31 | 0.24 | 0.29 | 0.34 | 0.35 | 0.37 | 0.35 |
| FFA 20:0 | 1.61 | 1.01 | 1.11 | 1.45 | 1.05 | 0.84 | 0.83 | 0.90 | 1.12 | 0.80 | 0.65 | 0.85 | 0.73 | 0.79 | 0.85 | 0.79 |
| FFA 20:1 | 0.61 | 0.42 | 0.47 | 0.58 | 0.25 | 0.24 | 0.22 | 0.24 | 0.40 | 0.38 | 0.44 | 0.41 | 0.41 | 0.43 | 0.42 | 0.42 |
| FFA 20:2 | 0.17 | 0.17 | 0.21 | 0.21 | 0.09 | 0.08 | 0.07 | 0.08 | 0.11 | 0.11 | 0.11 | 0.11 | 0.11 | 0.09 | 0.08 | 0.09 |
| FFA 20:3 | 0.27 | 0.28 | 0.31 | 0.33 | 0.17 | 0.17 | 0.17 | 0.17 | 0.27 | 0.25 | 0.29 | 0.27 | 0.35 | 0.32 | 0.39 | 0.35 |
| FFA 20:4 | 0.94 | 0.95 | 1.07 | 1.15 | 0.77 | 0.80 | 0.65 | 0.74 | 3.48 | 3.37 | 2.90 | 3.24 | 4.33 | 3.90 | 3.92 | 4.05 |
| FFA 20:5 | 0.08 | 0.10 | 0.11 | 0.11 | 0.06 | 0.06 | 0.04 | 0.05 | 2.34 | 2.29 | 2.23 | 2.28 | 2.04 | 2.63 | 2.44 | 2.37 |
| FFA 21:0 | 0.08 | 0.05 | 0.06 | 0.07 | 0.03 | 0.03 | 0.04 | 0.03 | 0.06 | 0.04 | 0.03 | 0.04 | 0.04 | 0.03 | 0.03 | 0.03 |
| FFA 21:1 | 0.06 | 0.07 | 0.08 | 0.08 | 0.03 | 0.03 | 0.03 | 0.03 | 0.04 | 0.04 | 0.04 | 0.04 | 0.04 | 0.04 | 0.05 | 0.04 |
| FFA 22:0 | 0.19 | 0.19 | 0.18 | 0.21 | 0.15 | 0.13 | 0.11 | 0.13 | 0.22 | 0.15 | 0.16 | 0.18 | 0.19 | 0.21 | 0.19 | 0.20 |
| FFA 22:1 | 2.08 | 1.19 | 1.30 | 1.77 | 0.52 | 0.48 | 0.37 | 0.45 | 0.52 | 0.41 | 0.40 | 0.44 | 0.43 | 0.38 | 0.39 | 0.40 |
| FFA 22:2 | 0.45 | 0.34 | 0.39 | 0.46 | 0.12 | 0.12 | 0.09 | 0.11 | 0.12 | 0.10 | 0.10 | 0.11 | 0.05 | 0.05 | 0.06 | 0.05 |
| FFA 22:3 | 0.18 | 0.18 | 0.21 | 0.22 | 0.21 | 0.22 | 0.21 | 0.21 | 0.23 | 0.23 | 0.23 | 0.23 | 0.13 | 0.12 | 0.16 | 0.14 |
| FFA 22:4 | 0.14 | 0.15 | 0.18 | 0.18 | 0.24 | 0.23 | 0.20 | 0.22 | 0.45 | 0.43 | 0.44 | 0.44 | 0.55 | 0.49 | 0.60 | 0.54 |
| FFA 22:5 | 0.56 | 0.57 | 0.64 | 0.69 | 0.92 | 0.92 | 0.75 | 0.86 | 5.73 | 5.55 | 4.35 | 5.18 | 7.09 | 6.22 | 8.44 | 7.22 |
| FFA 22:6 | 0.79 | 0.77 | 0.82 | 0.92 | 1.45 | 1.47 | 1.29 | 1.40 | 12.90 | 12.70 | 11.13 | 12.21 | 13.43 | 11.85 | 11.33 | 12.24 |
| FFA 23:0 | 0.09 | 0.11 | 0.12 | 0.12 | 0.10 | 0.08 | 0.09 | 0.09 | 0.13 | 0.10 | 0.08 | 0.10 | 0.10 | 0.09 | 0.10 | 0.10 |
| FFA 24:0 | 0.09 | 0.11 | 0.14 | 0.13 | 0.12 | 0.09 | 0.07 | 0.09 | 0.15 | 0.10 | 0.10 | 0.12 | 0.12 | 0.18 | 0.17 | 0.16 |
| FFA 24:1 | 0.14 | 0.13 | 0.16 | 0.17 | 0.09 | 0.08 | 0.08 | 0.08 | 0.11 | 0.09 | 0.10 | 0.10 | 0.09 | 0.10 | 0.11 | 0.10 |
| FFA 24:3 | 0.02 | 0.02 | 0.03 | 0.03 | 0.01 | 0.01 | 0.01 | 0.01 | 0.01 | 0.01 | 0.01 | 0.01 | 0.01 | 0.01 | 0.01 | 0.01 |
| FFA 25:0 | 0.03 | 0.04 | 0.04 | 0.05 | 0.04 | 0.03 | 0.03 | 0.03 | 0.04 | 0.03 | 0.03 | 0.03 | 0.03 | 0.03 | 0.03 | 0.03 |
| FFA 26:0 | 0.02 | 0.03 | 0.03 | 0.03 | 0.03 | 0.03 | 0.03 | 0.03 | 0.04 | 0.03 | 0.03 | 0.03 | 0.04 | 0.06 | 0.07 | 0.06 |
| FFA 26:1 | 0.02 | 0.02 | 0.02 | 0.03 | 0.01 | 0.01 | 0.01 | 0.01 | 0.02 | 0.02 | 0.02 | 0.02 | 0.01 | 0.01 | 0.02 | 0.01 |
| FFA 28:0 | 0.03 | 0.04 | 0.05 | 0.05 | 0.04 | 0.04 | 0.03 | 0.04 | 0.03 | 0.03 | 0.03 | 0.03 | 0.04 | 0.06 | 0.05 | 0.05 |
| Hex2Cer 34:1;2O\|Hex2Cer 18:1;2O/16:0 | 0.76 | 0.79 | 0.74 | 0.77 | 0.82 | 0.85 | 0.74 | 0.80 | 0.39 | 0.36 | 0.28 | 0.35 | 0.83 | 0.85 | 0.73 | 0.80 |
| Hex2Cer 34:2;2O\|Hex2Cer 18:1;2O/16:1 | 0.33 | 0.33 | 0.32 | 0.33 | 0.34 | 0.32 | 0.33 | 0.33 | 0.27 | 0.26 | 0.22 | 0.25 | 0.65 | 0.67 | 0.72 | 0.68 |
| Hex2Cer 35:1;2O\|Hex2Cer 18:1;2O/17:0 | 0.18 | 0.20 | 0.21 | 0.20 | 0.18 | 0.16 | 0.22 | 0.19 | 0.27 | 0.25 | 0.30 | 0.27 | 0.63 | 0.56 | 0.60 | 0.59 |
| Hex2Cer 36:1;2O\|Hex2Cer 18:1;2O/18:0 | 0.11 | 0.11 | 0.10 | 0.11 | 0.08 | 0.09 | 0.09 | 0.09 | 0.13 | 0.13 | 0.10 | 0.12 | 0.12 | 0.12 | 0.08 | 0.11 |
| Hex2Cer 36:2;2O\|Hex2Cer 18:1;2O/18:1 | 0.11 | 0.10 | 0.11 | 0.11 | 0.13 | 0.13 | 0.11 | 0.12 | 0.13 | 0.11 | 0.13 | 0.12 | 0.18 | 0.19 | 0.15 | 0.17 |
| Hex2Cer 38:1;2O\|Hex2Cer 18:1;2O/20:0 | 0.06 | 0.06 | 0.07 | 0.06 | 0.06 | 0.06 | 0.05 | 0.06 | 0.07 | 0.08 | 0.04 | 0.06 | 0.10 | 0.10 | 0.09 | 0.10 |
| Hex2Cer 38:2;2O\|Hex2Cer 16:1;2O/22:1 | 0.09 | 0.10 | 0.11 | 0.10 | 0.07 | 0.05 | 0.06 | 0.06 | 0.12 | 0.10 | 0.13 | 0.12 | 0.15 | 0.13 | 0.11 | 0.13 |
| Hex2Cer 39:1;2O\|Hex2Cer 18:1;2O/21:0 | 0.04 | 0.04 | 0.04 | 0.04 | 0.04 | 0.04 | 0.03 | 0.04 | 0.04 | 0.05 | 0.02 | 0.04 | 0.07 | 0.07 | 0.04 | 0.06 |
| Hex2Cer 40:1;2O\|Hex2Cer 18:1;2O/22:0 | 0.40 | 0.40 | 0.38 | 0.39 | 0.45 | 0.47 | 0.33 | 0.42 | 0.36 | 0.33 | 0.40 | 0.36 | 0.68 | 0.65 | 0.81 | 0.71 |
| Hex2Cer 40:2;2O\|Hex2Cer 18:1;2O/22:1 | 0.15 | 0.15 | 0.15 | 0.15 | 0.06 | 0.06 | 0.05 | 0.06 | 0.02 | 0.02 | 0.02 | 0.02 | 0.04 | 0.04 | 0.03 | 0.03 |
| Hex2Cer 41:1;2O\|Hex2Cer 18:1;2O/23:0 | 0.27 | 0.27 | 0.21 | 0.25 | 0.27 | 0.29 | 0.31 | 0.29 | 0.22 | 0.22 | 0.22 | 0.22 | 0.44 | 0.44 | 0.25 | 0.37 |
| Hex2Cer 42:1;2O\|Hex2Cer 18:1;2O/24:0 | 0.37 | 0.37 | 0.27 | 0.34 | 0.29 | 0.33 | 0.33 | 0.32 | 0.24 | 0.22 | 0.16 | 0.21 | 0.48 | 0.48 | 0.57 | 0.51 |
| Hex2Cer 43:1;2O\|Hex2Cer 18:1;2O/25:0 | 0.06 | 0.06 | 0.04 | 0.06 | 0.04 | 0.05 | 0.04 | 0.04 | 0.04 | 0.04 | 0.05 | 0.04 | 0.09 | 0.09 | 0.06 | 0.08 |
| Hex2Cer 44:1;2O\|Hex2Cer 20:1;2O/24:0 | 0.06 | 0.07 | 0.07 | 0.07 | 0.10 | 0.08 | 0.09 | 0.09 | 0.16 | 0.10 | 0.09 | 0.12 | 0.36 | 0.22 | 0.21 | 0.26 |
| HexCer 32:1;2O\|HexCer 18:1;2O/14:0 | 0.06 | 0.07 | 0.07 | 0.06 | 0.06 | 0.06 | 0.07 | 0.07 | 0.03 | 0.03 | 0.03 | 0.03 | 0.04 | 0.04 | 0.03 | 0.04 |
| HexCer 34:1;2O\|HexCer 18:1;2O/16:0 | 0.36 | 0.36 | 0.40 | 0.37 | 0.28 | 0.26 | 0.28 | 0.27 | 0.16 | 0.14 | 0.09 | 0.13 | 0.21 | 0.24 | 0.11 | 0.19 |
| HexCer 34:1;3O\|HexCer 18:1;2O/16:0;O | 0.05 | 0.05 | 0.06 | 0.06 | 0.04 | 0.04 | 0.02 | 0.03 | 0.03 | 0.03 | 0.02 | 0.03 | 0.04 | 0.04 | 0.02 | 0.03 |
| HexCer 34:2;2O\|HexCer 18:1;2O/16:1 | 0.07 | 0.07 | 0.05 | 0.06 | 0.06 | 0.06 | 0.07 | 0.06 | 0.02 | 0.02 | 0.02 | 0.02 | 0.05 | 0.05 | 0.05 | 0.05 |
| HexCer 35:1;2O\|HexCer 18:1;2O/17:0 | 0.35 | 0.34 | 0.28 | 0.32 | 0.57 | 0.56 | 0.58 | 0.57 | 0.53 | 0.58 | 0.42 | 0.51 | 0.57 | 0.59 | 0.49 | 0.55 |
| HexCer 36:1;2O\|HexCer 18:1;2O/18:0 | 0.20 | 0.18 | 0.12 | 0.17 | 0.12 | 0.11 | 0.10 | 0.11 | 0.13 | 0.13 | 0.10 | 0.12 | 0.09 | 0.12 | 0.08 | 0.10 |
| HexCer 39:1;2O\|HexCer 18:1;2O/21:0 | 0.31 | 0.32 | 0.28 | 0.30 | 0.45 | 0.45 | 0.32 | 0.41 | 0.49 | 0.44 | 0.27 | 0.40 | 0.46 | 0.50 | 0.51 | 0.49 |
| HexCer 40:1;2O\|HexCer 18:1;2O/22:0 | 0.34 | 0.32 | 0.28 | 0.31 | 0.26 | 0.28 | 0.21 | 0.25 | 0.10 | 0.10 | 0.12 | 0.11 | 0.19 | 0.19 | 0.17 | 0.19 |
| HexCer 41:1;2O\|HexCer 18:1;2O/23:0 | 1.04 | 1.03 | 1.09 | 1.05 | 0.86 | 0.93 | 0.55 | 0.78 | 0.85 | 0.79 | 0.87 | 0.84 | 0.91 | 0.88 | 0.93 | 0.91 |
| HexCer 42:1;2O\|HexCer 18:1;2O/24:0 | 0.26 | 0.28 | 0.18 | 0.24 | 0.23 | 0.22 | 0.25 | 0.23 | 0.09 | 0.10 | 0.08 | 0.09 | 0.15 | 0.14 | 0.13 | 0.14 |
| LPC 14:0 | 0.02 | 0.02 | 0.02 | 0.02 | 0.01 | 0.02 | 0.02 | 0.02 | 0.01 | 0.01 | 0.01 | 0.01 | 0.01 | 0.01 | 0.01 | 0.01 |
| LPC 16:0 | 0.30 | 0.21 | 0.22 | 0.24 | 0.24 | 0.23 | 0.19 | 0.22 | 0.45 | 0.40 | 0.49 | 0.45 | 0.40 | 0.29 | 0.28 | 0.32 |
| LPC 16:1 | 0.01 | 0.01 | 0.01 | 0.01 | 0.01 | 0.01 | 0.01 | 0.01 | 0.01 | 0.01 | 0.01 | 0.01 | 0.01 | 0.01 | 0.02 | 0.01 |
| LPC 17:0 | 0.03 | 0.02 | 0.02 | 0.02 | 0.03 | 0.02 | 0.02 | 0.02 | 0.04 | 0.04 | 0.04 | 0.04 | 0.04 | 0.03 | 0.03 | 0.03 |
| LPC 18:0 | 0.17 | 0.12 | 0.16 | 0.15 | 0.11 | 0.11 | 0.11 | 0.11 | 0.15 | 0.16 | 0.12 | 0.14 | 0.07 | 0.07 | 0.08 | 0.08 |
| LPC 18:1 | 0.37 | 0.25 | 0.22 | 0.28 | 0.24 | 0.23 | 0.26 | 0.24 | 0.46 | 0.44 | 0.52 | 0.48 | 0.55 | 0.38 | 0.32 | 0.42 |
| LPC 18:2 | 0.10 | 0.07 | 0.08 | 0.08 | 0.07 | 0.07 | 0.09 | 0.08 | 0.07 | 0.06 | 0.07 | 0.07 | 0.11 | 0.10 | 0.11 | 0.11 |
| LPC 20:4 | 0.04 | 0.04 | 0.04 | 0.04 | 0.02 | 0.03 | 0.02 | 0.02 | 0.05 | 0.05 | 0.06 | 0.05 | 0.05 | 0.04 | 0.05 | 0.05 |
| LPC 22:5 | 0.01 | 0.01 | 0.01 | 0.01 | 0.01 | 0.01 | 0.01 | 0.01 | 0.03 | 0.03 | 0.02 | 0.02 | 0.07 | 0.06 | 0.07 | 0.06 |
| LPC 22:6 | 0.01 | 0.01 | 0.01 | 0.01 | 0.01 | 0.01 | 0.01 | 0.01 | 0.05 | 0.04 | 0.04 | 0.04 | 0.08 | 0.07 | 0.07 | 0.07 |
| LPE 14:0 | 0.02 | 0.01 | 0.01 | 0.01 | 0.01 | 0.01 | 0.01 | 0.01 | 0.02 | 0.01 | 0.01 | 0.01 | 0.01 | 0.02 | 0.02 | 0.01 |
| LPE 16:0 | 0.15 | 0.15 | 0.15 | 0.15 | 0.11 | 0.16 | 0.13 | 0.13 | 0.32 | 0.32 | 0.38 | 0.34 | 0.39 | 0.26 | 0.25 | 0.30 |
| LPE 17:0 | 0.02 | 0.01 | 0.01 | 0.01 | 0.01 | 0.01 | 0.01 | 0.01 | 0.02 | 0.02 | 0.02 | 0.02 | 0.02 | 0.01 | 0.01 | 0.01 |
| LPE 18:0 | 0.08 | 0.09 | 0.11 | 0.09 | 0.05 | 0.08 | 0.07 | 0.06 | 0.12 | 0.14 | 0.15 | 0.14 | 0.13 | 0.10 | 0.11 | 0.12 |
| LPE 18:1 | 0.74 | 0.73 | 0.76 | 0.74 | 0.44 | 0.63 | 0.49 | 0.51 | 0.93 | 0.94 | 1.12 | 1.00 | 1.21 | 0.79 | 0.78 | 0.92 |
| LPE 18:2 | 0.75 | 0.67 | 0.50 | 0.64 | 0.43 | 0.63 | 0.66 | 0.56 | 0.35 | 0.35 | 0.42 | 0.37 | 0.53 | 0.35 | 0.40 | 0.43 |
| LPE 20:3 | 0.06 | 0.06 | 0.06 | 0.06 | 0.02 | 0.03 | 0.02 | 0.03 | 0.03 | 0.03 | 0.04 | 0.03 | 0.03 | 0.02 | 0.02 | 0.02 |
| LPE 20:4 | 0.08 | 0.08 | 0.09 | 0.08 | 0.03 | 0.05 | 0.05 | 0.04 | 0.13 | 0.12 | 0.10 | 0.12 | 0.08 | 0.06 | 0.06 | 0.06 |
| LPE 20:5 | 0.01 | 0.01 | 0.01 | 0.01 | 0.01 | 0.01 | 0.01 | 0.01 | 0.06 | 0.05 | 0.05 | 0.05 | 0.03 | 0.02 | 0.03 | 0.03 |
| LPE 22:4 | 0.03 | 0.01 | 0.02 | 0.02 | 0.02 | 0.02 | 0.02 | 0.02 | 0.02 | 0.02 | 0.02 | 0.02 | 0.03 | 0.03 | 0.03 | 0.03 |
| LPE 22:5 | 0.10 | 0.05 | 0.05 | 0.07 | 0.07 | 0.08 | 0.08 | 0.08 | 0.13 | 0.12 | 0.13 | 0.13 | 0.13 | 0.11 | 0.12 | 0.12 |
| LPE 22:6 | 0.11 | 0.09 | 0.07 | 0.09 | 0.12 | 0.17 | 0.15 | 0.14 | 0.61 | 0.59 | 0.58 | 0.59 | 0.64 | 0.49 | 0.65 | 0.59 |
| PA 36:1\|PA 18:0_18:1 | 0.99 | 0.96 | 1.07 | 1.01 | 0.91 | 0.69 | 0.71 | 0.76 | 0.52 | 0.52 | 0.42 | 0.49 | 0.37 | 0.31 | 0.34 | 0.34 |
| PA 36:2\|PA 18:1_18:1 | 0.74 | 0.70 | 0.66 | 0.70 | 1.20 | 1.02 | 1.37 | 1.19 | 1.27 | 1.59 | 1.28 | 1.37 | 1.50 | 1.21 | 1.03 | 1.24 |
| PA 36:3\|PA 18:1_18:2 | 0.30 | 0.22 | 0.28 | 0.26 | 0.56 | 0.47 | 0.43 | 0.48 | 0.21 | 0.24 | 0.19 | 0.21 | 0.27 | 0.22 | 0.24 | 0.25 |
| PA 39:6\|PA 17:0_22:6 | 0.09 | 0.08 | 0.07 | 0.08 | 0.08 | 0.08 | 0.11 | 0.09 | 0.12 | 0.14 | 0.14 | 0.13 | 0.13 | 0.12 | 0.12 | 0.12 |
| PA 40:7\|PA 18:2_22:5 | 1.25 | 0.74 | 0.82 | 0.93 | 1.03 | 1.01 | 1.15 | 1.06 | 1.31 | 1.63 | 1.24 | 1.38 | 1.65 | 1.47 | 1.95 | 1.69 |
| PC 20:0\|PC 8:0_12:0 | 0.01 | 0.00 | 0.01 | 0.01 | 0.01 | 0.01 | 0.01 | 0.01 | 0.02 | 0.02 | 0.02 | 0.02 | 0.03 | 0.04 | 0.04 | 0.04 |
| PC 22:0\|PC 10:0_12:0 | 0.06 | 0.03 | 0.04 | 0.04 | 0.07 | 0.06 | 0.06 | 0.06 | 0.10 | 0.10 | 0.09 | 0.10 | 0.10 | 0.11 | 0.11 | 0.11 |
| PC 24:0\|PC 10:0_14:0 | 0.21 | 0.13 | 0.14 | 0.15 | 0.16 | 0.16 | 0.14 | 0.15 | 0.18 | 0.20 | 0.15 | 0.18 | 0.20 | 0.21 | 0.27 | 0.23 |
| PC 25:0\|PC 10:0_15:0 | 0.04 | 0.03 | 0.03 | 0.03 | 0.05 | 0.05 | 0.04 | 0.05 | 0.04 | 0.06 | 0.05 | 0.05 | 0.10 | 0.11 | 0.14 | 0.12 |
| PC 26:0\|PC 10:0_16:0 | 0.58 | 0.34 | 0.40 | 0.43 | 0.39 | 0.37 | 0.50 | 0.42 | 0.41 | 0.48 | 0.38 | 0.43 | 0.52 | 0.54 | 0.64 | 0.57 |
| PC 26:1\|PC 8:0_18:1 | 0.04 | 0.03 | 0.04 | 0.03 | 0.04 | 0.03 | 0.04 | 0.04 | 0.04 | 0.04 | 0.05 | 0.04 | 0.07 | 0.07 | 0.07 | 0.07 |
| PC 27:0\|PC 12:0_15:0 | 0.29 | 0.21 | 0.23 | 0.24 | 0.19 | 0.18 | 0.15 | 0.17 | 0.21 | 0.24 | 0.18 | 0.21 | 0.33 | 0.32 | 0.32 | 0.32 |
| PC 28:0\|PC 14:0_14:0 | 1.22 | 0.74 | 1.01 | 0.97 | 0.47 | 0.44 | 0.52 | 0.48 | 0.60 | 0.64 | 0.74 | 0.66 | 0.62 | 0.64 | 0.63 | 0.63 |
| PC 28:1\|PC 10:0_18:1 | 0.16 | 0.10 | 0.10 | 0.12 | 0.12 | 0.11 | 0.12 | 0.12 | 0.12 | 0.14 | 0.12 | 0.12 | 0.18 | 0.18 | 0.21 | 0.19 |
| PC 28:2\|PC 10:0_18:2 | 0.03 | 0.02 | 0.02 | 0.02 | 0.02 | 0.02 | 0.02 | 0.02 | 0.03 | 0.03 | 0.02 | 0.03 | 0.06 | 0.07 | 0.07 | 0.07 |
| PC 29:0\|PC 14:0_15:0 | 0.33 | 0.23 | 0.30 | 0.28 | 0.17 | 0.15 | 0.13 | 0.15 | 0.20 | 0.23 | 0.25 | 0.23 | 0.29 | 0.23 | 0.23 | 0.25 |
| PC 30:0\|PC 14:0_16:0 | 5.66 | 3.80 | 4.31 | 4.52 | 2.07 | 2.08 | 1.83 | 2.00 | 2.24 | 2.80 | 3.11 | 2.70 | 2.84 | 2.85 | 3.36 | 3.02 |
| PC 30:1\|PC 12:0_18:1 | 0.57 | 0.36 | 0.41 | 0.44 | 0.25 | 0.24 | 0.21 | 0.23 | 0.24 | 0.26 | 0.29 | 0.26 | 0.32 | 0.34 | 0.44 | 0.37 |
| PC 31:0\|PC 15:0_16:0 | 1.29 | 0.85 | 1.13 | 1.07 | 0.83 | 0.74 | 0.72 | 0.76 | 0.88 | 1.09 | 0.91 | 0.95 | 1.16 | 1.12 | 1.17 | 1.15 |
| PC 32:0\|PC 16:0_16:0 | 13.73 | 10.19 | 14.66 | 12.71 | 8.16 | 7.04 | 9.50 | 8.22 | 12.00 | 13.49 | 12.07 | 12.48 | 14.80 | 14.14 | 11.79 | 13.58 |
| PC 32:1\|PC 14:0_18:1 | 5.74 | 3.69 | 4.92 | 4.70 | 2.72 | 2.52 | 3.32 | 2.85 | 2.11 | 2.76 | 2.16 | 2.32 | 3.69 | 3.94 | 3.57 | 3.73 |
| PC 32:2\|PC 14:0_18:2 | 2.26 | 1.36 | 1.44 | 1.65 | 1.04 | 0.87 | 0.85 | 0.92 | 0.60 | 0.70 | 0.78 | 0.69 | 1.35 | 1.39 | 1.61 | 1.45 |
| PC 33:0\|PC 16:0_17:0 | 2.27 | 1.60 | 1.69 | 1.82 | 1.32 | 1.20 | 1.59 | 1.37 | 1.99 | 2.37 | 2.40 | 2.24 | 2.57 | 2.62 | 2.73 | 2.64 |
| PC 33:1\|PC 15:0_18:1 | 0.09 | 0.14 | 0.09 | 0.10 | 0.21 | 0.23 | 0.19 | 0.21 | 0.18 | 0.19 | 0.22 | 0.19 | 0.20 | 0.19 | 0.25 | 0.21 |
| PC 34:0\|PC 16:0_18:0 | 5.28 | 4.12 | 5.64 | 4.97 | 2.23 | 2.20 | 2.57 | 2.33 | 4.42 | 5.11 | 4.97 | 4.81 | 3.96 | 3.25 | 3.64 | 3.62 |
| PC 34:1\|PC 16:0_18:1 | 49.23 | 32.58 | 43.73 | 41.15 | 27.59 | 23.99 | 23.59 | 25.00 | 30.80 | 35.78 | 28.56 | 31.56 | 46.40 | 49.03 | 47.07 | 47.50 |
| PC 34:2\|PC 16:0_18:2 | 22.17 | 14.33 | 19.00 | 18.17 | 13.13 | 11.68 | 12.58 | 12.44 | 9.56 | 11.67 | 11.13 | 10.72 | 22.00 | 23.11 | 21.27 | 22.13 |
| PC 34:3\|PC 16:1_18:2 | 1.67 | 1.02 | 1.31 | 1.31 | 1.26 | 1.10 | 0.98 | 1.11 | 0.51 | 0.59 | 0.58 | 0.56 | 1.81 | 1.77 | 2.15 | 1.91 |
| PC 34:4\|PC 14:0_20:4 | 0.20 | 0.12 | 0.14 | 0.15 | 0.14 | 0.14 | 0.16 | 0.15 | 0.11 | 0.13 | 0.12 | 0.12 | 0.25 | 0.26 | 0.23 | 0.25 |
| PC 35:0\|PC 16:0_19:0 | 0.64 | 0.41 | 0.44 | 0.49 | 0.27 | 0.26 | 0.22 | 0.25 | 0.56 | 0.70 | 0.62 | 0.62 | 0.57 | 0.52 | 0.60 | 0.56 |
| PC 35:1\|PC 17:0_18:1 | 4.59 | 2.95 | 2.91 | 3.42 | 2.34 | 2.18 | 2.64 | 2.38 | 3.83 | 4.56 | 3.54 | 3.96 | 5.43 | 5.79 | 5.58 | 5.60 |
| PC 35:2\|PC 17:0_18:2 | 0.07 | 0.10 | 0.06 | 0.07 | 0.13 | 0.13 | 0.16 | 0.14 | 0.14 | 0.15 | 0.11 | 0.13 | 0.12 | 0.11 | 0.13 | 0.12 |
| PC 35:2\|PC 17:1_18:1 | 1.66 | 1.14 | 1.17 | 1.30 | 0.83 | 0.74 | 1.00 | 0.85 | 0.91 | 1.17 | 1.20 | 1.08 | 1.72 | 1.63 | 1.65 | 1.67 |
| PC 36:0\|PC 18:0_18:0 | 0.89 | 0.76 | 0.83 | 0.82 | 0.44 | 0.43 | 0.52 | 0.47 | 0.91 | 1.24 | 0.96 | 1.03 | 1.12 | 0.96 | 1.05 | 1.04 |
| PC 36:1\|PC 18:0_18:1 | 15.61 | 9.81 | 10.24 | 11.64 | 4.91 | 4.78 | 6.12 | 5.27 | 7.27 | 8.60 | 9.99 | 8.58 | 8.77 | 8.86 | 9.72 | 9.11 |
| PC 36:2\|PC 18:1_18:1 | 28.11 | 18.44 | 21.87 | 22.40 | 16.62 | 14.52 | 16.12 | 15.72 | 16.09 | 19.02 | 19.18 | 18.01 | 24.88 | 27.17 | 26.64 | 26.23 |
| PC 36:3\|PC 18:1_18:2 | 16.37 | 10.23 | 11.45 | 12.42 | 10.22 | 8.96 | 7.71 | 8.94 | 7.01 | 8.59 | 6.87 | 7.44 | 13.82 | 14.63 | 16.34 | 14.93 |
| PC 36:4\|PC 18:2_18:2 | 3.28 | 2.03 | 1.99 | 2.38 | 2.33 | 2.06 | 2.56 | 2.31 | 1.36 | 1.68 | 1.61 | 1.54 | 4.00 | 4.03 | 3.59 | 3.88 |
| PC 36:5\|PC 16:0_20:5 | 0.26 | 0.18 | 0.17 | 0.20 | 0.20 | 0.19 | 0.20 | 0.20 | 0.79 | 0.84 | 0.72 | 0.78 | 1.49 | 1.55 | 1.76 | 1.60 |
| PC 36:5\|PC 18:2_18:3 | 0.42 | 0.33 | 0.35 | 0.36 | 0.26 | 0.22 | 0.29 | 0.26 | 0.17 | 0.23 | 0.23 | 0.21 | 0.40 | 0.43 | 0.37 | 0.40 |
| PC 36:6\|PC 14:0_22:6 | 0.07 | 0.04 | 0.04 | 0.05 | 0.14 | 0.08 | 0.09 | 0.10 | 0.19 | 0.21 | 0.21 | 0.20 | 0.51 | 0.47 | 0.55 | 0.51 |
| PC 37:1\|PC 18:0_19:1 | 0.40 | 0.26 | 0.33 | 0.32 | 0.15 | 0.15 | 0.16 | 0.15 | 0.30 | 0.41 | 0.43 | 0.37 | 0.38 | 0.40 | 0.49 | 0.42 |
| PC 37:2\|PC 18:1_19:1 | 0.04 | 0.06 | 0.06 | 0.05 | 0.06 | 0.07 | 0.07 | 0.07 | 0.08 | 0.09 | 0.09 | 0.08 | 0.07 | 0.06 | 0.07 | 0.07 |
| PC 38:1\|PC 16:0_22:1 | 1.24 | 0.77 | 0.96 | 0.97 | 0.30 | 0.30 | 0.36 | 0.32 | 0.59 | 0.77 | 0.73 | 0.69 | 0.79 | 0.84 | 0.91 | 0.85 |
| PC 38:2\|PC 18:1_20:1 | 2.27 | 1.46 | 1.67 | 1.77 | 0.91 | 0.86 | 0.90 | 0.89 | 1.30 | 1.49 | 1.65 | 1.47 | 1.75 | 1.87 | 2.33 | 1.98 |
| PC 38:3\|PC 18:0_20:3 | 1.37 | 0.93 | 0.91 | 1.05 | 0.67 | 0.61 | 0.81 | 0.70 | 0.65 | 0.77 | 0.90 | 0.77 | 1.27 | 1.31 | 1.30 | 1.29 |
| PC 38:4\|PC 16:0_22:4 | 1.03 | 0.74 | 0.94 | 0.89 | 0.58 | 0.50 | 0.47 | 0.51 | 0.60 | 0.73 | 0.66 | 0.66 | 1.45 | 1.50 | 1.49 | 1.48 |
| PC 38:5\|PC 16:0_22:5 | 1.18 | 0.82 | 1.00 | 0.98 | 0.90 | 0.81 | 1.09 | 0.93 | 1.98 | 2.56 | 3.02 | 2.50 | 5.49 | 5.74 | 5.79 | 5.67 |
| PC 38:6\|PC 16:0_22:6 | 0.97 | 0.61 | 0.69 | 0.74 | 0.87 | 0.74 | 0.92 | 0.84 | 3.09 | 3.91 | 4.58 | 3.84 | 7.25 | 7.36 | 8.02 | 7.55 |
| PC 38:7\|PC 16:1_22:6 | 0.11 | 0.06 | 0.07 | 0.08 | 0.14 | 0.12 | 0.12 | 0.13 | 0.27 | 0.32 | 0.27 | 0.28 | 0.65 | 0.66 | 0.83 | 0.72 |
| PC 39:6\|PC 17:0_22:6 | 0.05 | 0.03 | 0.04 | 0.04 | 0.04 | 0.04 | 0.04 | 0.04 | 0.12 | 0.16 | 0.13 | 0.13 | 0.28 | 0.28 | 0.32 | 0.29 |
| PC 39:7\|PC 17:1_22:6 | 0.03 | 0.02 | 0.02 | 0.02 | 0.02 | 0.02 | 0.02 | 0.02 | 0.05 | 0.05 | 0.04 | 0.04 | 0.07 | 0.07 | 0.08 | 0.07 |
| PC 40:3\|PC 18:0_22:3 | 0.65 | 0.42 | 0.42 | 0.48 | 0.18 | 0.17 | 0.17 | 0.17 | 0.14 | 0.18 | 0.19 | 0.17 | 0.13 | 0.12 | 0.10 | 0.12 |
| PC 40:4\|PC 18:1_22:3 | 0.65 | 0.45 | 0.60 | 0.56 | 0.37 | 0.35 | 0.32 | 0.34 | 0.36 | 0.44 | 0.50 | 0.43 | 0.73 | 0.73 | 0.85 | 0.77 |
| PC 40:5\|PC 18:1_22:4 | 0.34 | 0.21 | 0.21 | 0.24 | 0.26 | 0.23 | 0.25 | 0.25 | 0.28 | 0.35 | 0.34 | 0.32 | 0.72 | 0.70 | 0.89 | 0.77 |
| PC 40:6\|PC 18:1_22:5 | 0.76 | 0.50 | 0.62 | 0.62 | 0.75 | 0.42 | 0.45 | 0.54 | 1.52 | 2.04 | 1.50 | 1.67 | 3.64 | 3.68 | 4.64 | 3.99 |
| PC 40:7\|PC 18:1_22:6 | 0.92 | 0.57 | 0.61 | 0.69 | 1.03 | 0.91 | 1.01 | 0.98 | 2.20 | 2.78 | 2.48 | 2.47 | 4.98 | 5.18 | 4.28 | 4.82 |
| PC 40:8\|PC 18:2_22:6 | 0.46 | 0.29 | 0.34 | 0.36 | 0.68 | 0.62 | 0.59 | 0.63 | 1.02 | 1.11 | 1.31 | 1.14 | 2.25 | 2.32 | 2.82 | 2.46 |
| PC 40:9\|PC 18:3_22:6 | 0.04 | 0.02 | 0.03 | 0.03 | 0.07 | 0.06 | 0.08 | 0.07 | 0.08 | 0.09 | 0.09 | 0.09 | 0.20 | 0.20 | 0.23 | 0.21 |
| PC 42:10\|PC 20:4_22:6 | 0.04 | 0.03 | 0.04 | 0.04 | 0.08 | 0.07 | 0.07 | 0.07 | 0.25 | 0.31 | 0.34 | 0.30 | 0.48 | 0.49 | 0.60 | 0.52 |
| PC 42:11\|PC 20:5_22:6 | 0.02 | 0.01 | 0.02 | 0.02 | 0.03 | 0.02 | 0.03 | 0.03 | 0.13 | 0.16 | 0.18 | 0.16 | 0.19 | 0.20 | 0.17 | 0.19 |
| PC 42:7\|PC 20:1_22:6 | 0.04 | 0.02 | 0.03 | 0.03 | 0.03 | 0.03 | 0.03 | 0.03 | 0.05 | 0.06 | 0.06 | 0.06 | 0.10 | 0.09 | 0.12 | 0.10 |
| PC 42:9\|PC 20:4_22:5 | 0.05 | 0.04 | 0.04 | 0.04 | 0.05 | 0.04 | 0.04 | 0.04 | 0.10 | 0.10 | 0.10 | 0.10 | 0.21 | 0.22 | 0.24 | 0.22 |
| PC 44:10\|PC 22:4_22:6 | 0.03 | 0.02 | 0.03 | 0.03 | 0.04 | 0.04 | 0.04 | 0.04 | 0.10 | 0.12 | 0.13 | 0.12 | 0.25 | 0.27 | 0.23 | 0.25 |
| PC 44:11\|PC 22:5_22:6 | 0.02 | 0.02 | 0.02 | 0.02 | 0.02 | 0.02 | 0.02 | 0.02 | 0.06 | 0.07 | 0.08 | 0.07 | 0.12 | 0.12 | 0.14 | 0.13 |
| PC 44:12\|PC 22:6_22:6 | 0.05 | 0.03 | 0.03 | 0.04 | 0.10 | 0.10 | 0.08 | 0.09 | 0.58 | 0.70 | 0.61 | 0.62 | 1.18 | 1.18 | 1.37 | 1.24 |
| PE 24:0\|PE 10:0_14:0 | 0.02 | 0.01 | 0.01 | 0.02 | 0.01 | 0.01 | 0.01 | 0.01 | 0.01 | 0.01 | 0.00 | 0.01 | 0.01 | 0.01 | 0.01 | 0.01 |
| PE 26:0\|PE 10:0_16:0 | 0.01 | 0.01 | 0.02 | 0.01 | 0.01 | 0.01 | 0.01 | 0.01 | 0.02 | 0.02 | 0.02 | 0.02 | 0.01 | 0.01 | 0.02 | 0.01 |
| PE 28:0\|PE 12:0_16:0 | 0.14 | 0.15 | 0.18 | 0.16 | 0.09 | 0.08 | 0.08 | 0.08 | 0.06 | 0.07 | 0.06 | 0.06 | 0.06 | 0.06 | 0.07 | 0.06 |
| PE 28:1\|PE 10:0_18:1 | 0.09 | 0.05 | 0.06 | 0.07 | 0.05 | 0.04 | 0.06 | 0.05 | 0.04 | 0.04 | 0.04 | 0.04 | 0.06 | 0.06 | 0.07 | 0.06 |
| PE 30:0\|PE 14:0_16:0 | 0.93 | 0.83 | 0.86 | 0.87 | 0.22 | 0.19 | 0.25 | 0.22 | 0.59 | 0.63 | 0.71 | 0.64 | 0.43 | 0.40 | 0.35 | 0.39 |
| PE 30:1\|PE 12:0_18:1 | 0.22 | 0.21 | 0.21 | 0.21 | 0.22 | 0.19 | 0.24 | 0.22 | 0.11 | 0.13 | 0.13 | 0.12 | 0.11 | 0.11 | 0.11 | 0.11 |
| PE 30:2\|PE 12:0_18:2 | 0.04 | 0.04 | 0.04 | 0.04 | 0.04 | 0.03 | 0.05 | 0.04 | 0.02 | 0.02 | 0.02 | 0.02 | 0.03 | 0.02 | 0.03 | 0.03 |
| PE 31:0\|PE 15:0_16:0 | 0.09 | 0.09 | 0.07 | 0.08 | 0.07 | 0.06 | 0.08 | 0.07 | 0.09 | 0.10 | 0.08 | 0.09 | 0.10 | 0.09 | 0.07 | 0.08 |
| PE 31:1\|PE 13:0_18:1 | 0.09 | 0.09 | 0.07 | 0.08 | 0.07 | 0.05 | 0.07 | 0.06 | 0.06 | 0.06 | 0.05 | 0.06 | 0.05 | 0.05 | 0.05 | 0.05 |
| PE 32:0\|PE 16:0_16:0 | 0.44 | 0.50 | 0.51 | 0.48 | 0.26 | 0.21 | 0.21 | 0.23 | 0.79 | 0.84 | 0.81 | 0.81 | 0.82 | 0.63 | 0.50 | 0.65 |
| PE 32:1\|PE 14:0_18:1 | 3.29 | 2.74 | 2.74 | 2.93 | 1.33 | 1.15 | 1.15 | 1.21 | 1.10 | 1.20 | 1.06 | 1.12 | 0.85 | 0.90 | 0.95 | 0.90 |
| PE 32:2\|PE 14:0_18:2 | 1.18 | 1.10 | 1.39 | 1.22 | 1.07 | 0.98 | 1.15 | 1.06 | 0.43 | 0.48 | 0.37 | 0.42 | 0.47 | 0.51 | 0.59 | 0.52 |
| PE 33:0\|PE 16:0_17:0 | 0.20 | 0.19 | 0.19 | 0.19 | 0.17 | 0.14 | 0.19 | 0.16 | 0.25 | 0.27 | 0.27 | 0.26 | 0.28 | 0.24 | 0.20 | 0.24 |
| PE 33:1\|PE 15:0_18:1 | 0.41 | 0.40 | 0.49 | 0.44 | 0.30 | 0.25 | 0.28 | 0.27 | 0.29 | 0.32 | 0.26 | 0.29 | 0.25 | 0.25 | 0.27 | 0.26 |
| PE 33:2\|PE 15:0_18:2 | 0.81 | 0.65 | 0.63 | 0.70 | 0.66 | 0.58 | 0.53 | 0.58 | 0.29 | 0.35 | 0.33 | 0.32 | 0.30 | 0.31 | 0.36 | 0.33 |
| PE 34:0\|PE 16:0_18:0 | 0.68 | 0.75 | 0.88 | 0.77 | 0.54 | 0.43 | 0.44 | 0.47 | 1.15 | 1.26 | 1.27 | 1.22 | 1.03 | 0.79 | 0.85 | 0.89 |
| PE 34:1\|PE 16:0_18:1 | 17.48 | 15.21 | 19.36 | 17.36 | 9.80 | 8.68 | 8.46 | 8.95 | 13.67 | 14.47 | 11.48 | 13.19 | 13.02 | 13.12 | 11.49 | 12.54 |
| PE 34:2\|PE 16:0_18:2 | 29.02 | 25.75 | 32.83 | 29.22 | 22.72 | 18.74 | 21.37 | 20.83 | 11.91 | 13.20 | 13.21 | 12.74 | 14.47 | 14.62 | 14.47 | 14.52 |
| PE 34:3\|PE 16:0_18:3 | 0.85 | 0.66 | 0.72 | 0.74 | 1.07 | 1.01 | 1.11 | 1.06 | 0.24 | 0.29 | 0.23 | 0.25 | 0.45 | 0.41 | 0.34 | 0.40 |
| PE 34:3\|PE 16:1_18:2 | 6.17 | 5.79 | 6.75 | 6.24 | 9.26 | 7.88 | 6.86 | 7.96 | 1.87 | 2.13 | 2.24 | 2.08 | 3.46 | 3.66 | 4.35 | 3.82 |
| PE 34:4\|PE 16:1_18:3 | 0.15 | 0.14 | 0.15 | 0.15 | 0.20 | 0.18 | 0.20 | 0.19 | 0.09 | 0.11 | 0.09 | 0.10 | 0.13 | 0.13 | 0.12 | 0.13 |
| PE 35:1\|PE 17:0_18:1 | 2.63 | 2.49 | 3.03 | 2.71 | 1.60 | 1.43 | 1.84 | 1.62 | 3.11 | 3.49 | 3.93 | 3.50 | 2.81 | 2.69 | 2.28 | 2.59 |
| PE 35:2\|PE 17:0_18:2 | 2.00 | 1.83 | 2.23 | 2.02 | 1.66 | 1.33 | 1.33 | 1.43 | 1.24 | 1.41 | 1.41 | 1.35 | 1.25 | 1.30 | 1.31 | 1.29 |
| PE 35:3\|PE 17:1_18:2 | 0.57 | 0.51 | 0.65 | 0.58 | 0.43 | 0.37 | 0.41 | 0.40 | 0.21 | 0.26 | 0.31 | 0.26 | 0.19 | 0.20 | 0.26 | 0.22 |
| PE 36:0\|PE 18:0_18:0 | 0.28 | 0.41 | 0.47 | 0.38 | 0.19 | 0.17 | 0.20 | 0.19 | 0.42 | 0.47 | 0.45 | 0.45 | 0.43 | 0.23 | 0.21 | 0.29 |
| PE 36:1\|PE 18:0_18:1 | 25.03 | 21.44 | 21.23 | 22.58 | 12.50 | 10.08 | 8.90 | 10.43 | 15.15 | 18.37 | 16.59 | 16.63 | 11.84 | 12.34 | 15.53 | 13.23 |
| PE 36:2\|PE 18:1_18:1 | 55.87 | 51.64 | 55.04 | 54.20 | 41.25 | 35.11 | 30.46 | 35.44 | 41.46 | 46.77 | 35.04 | 40.97 | 38.10 | 37.16 | 43.40 | 39.56 |
| PE 36:3\|PE 18:1_18:2 | 50.08 | 43.94 | 39.83 | 44.65 | 44.97 | 36.85 | 33.53 | 38.23 | 27.11 | 30.38 | 33.02 | 30.09 | 28.92 | 29.97 | 24.53 | 27.80 |
| PE 36:4\|PE 18:2_18:2 | 38.42 | 35.37 | 38.53 | 37.45 | 41.52 | 35.86 | 40.00 | 38.97 | 14.74 | 16.40 | 15.16 | 15.40 | 20.61 | 22.14 | 28.30 | 23.68 |
| PE 36:5\|PE 14:0_22:5 | 0.46 | 0.30 | 0.41 | 0.38 | 0.58 | 0.57 | 0.52 | 0.56 | 0.37 | 0.49 | 0.49 | 0.45 | 0.54 | 0.54 | 0.67 | 0.58 |
| PE 36:5\|PE 16:0_20:5 | 1.02 | 0.87 | 0.92 | 0.94 | 1.93 | 1.71 | 2.33 | 1.99 | 2.40 | 2.88 | 3.31 | 2.85 | 2.70 | 2.81 | 3.31 | 2.94 |
| PE 36:6\|PE 14:0_22:6 | 0.10 | 0.10 | 0.11 | 0.10 | 0.19 | 0.17 | 0.16 | 0.18 | 0.27 | 0.30 | 0.31 | 0.29 | 0.37 | 0.36 | 0.32 | 0.35 |
| PE 37:0\|PE 16:0_21:0 | 0.11 | 0.13 | 0.12 | 0.12 | 0.09 | 0.08 | 0.10 | 0.09 | 0.19 | 0.23 | 0.21 | 0.21 | 0.20 | 0.12 | 0.13 | 0.15 |
| PE 37:1\|PE 17:0_20:1 | 0.93 | 0.85 | 1.04 | 0.94 | 0.51 | 0.45 | 0.41 | 0.46 | 0.87 | 0.97 | 1.13 | 0.99 | 0.69 | 0.69 | 0.81 | 0.73 |
| PE 37:2\|PE 18:1_19:1 | 1.08 | 1.08 | 1.27 | 1.14 | 0.77 | 0.64 | 0.76 | 0.72 | 1.12 | 1.28 | 1.21 | 1.20 | 0.87 | 0.94 | 1.17 | 0.99 |
| PE 37:3\|PE 19:1_18:2 | 0.35 | 0.33 | 0.26 | 0.31 | 0.28 | 0.22 | 0.28 | 0.26 | 0.23 | 0.26 | 0.31 | 0.26 | 0.22 | 0.21 | 0.23 | 0.22 |
| PE 37:5\|PE 15:0_22:5 | 0.15 | 0.16 | 0.15 | 0.15 | 0.20 | 0.17 | 0.21 | 0.19 | 0.38 | 0.48 | 0.56 | 0.47 | 0.35 | 0.35 | 0.39 | 0.36 |
| PE 38:0\|PE 18:0_20:0 | 0.14 | 0.19 | 0.16 | 0.16 | 0.12 | 0.10 | 0.10 | 0.11 | 0.22 | 0.26 | 0.27 | 0.25 | 0.20 | 0.11 | 0.12 | 0.15 |
| PE 38:1\|PE 18:0_20:1 | 2.54 | 2.39 | 1.94 | 2.29 | 1.27 | 1.14 | 1.12 | 1.17 | 2.70 | 3.23 | 3.42 | 3.11 | 2.69 | 2.27 | 2.05 | 2.34 |
| PE 38:2\|PE 18:1_20:1 | 5.72 | 4.76 | 4.59 | 5.03 | 5.40 | 4.49 | 5.86 | 5.23 | 6.50 | 7.96 | 7.59 | 7.32 | 7.76 | 8.24 | 9.16 | 8.38 |
| PE 38:3\|PE 18:0_20:3 | 3.06 | 2.81 | 2.77 | 2.88 | 2.51 | 2.05 | 1.88 | 2.13 | 2.08 | 2.46 | 2.54 | 2.35 | 2.54 | 2.73 | 2.44 | 2.57 |
| PE 38:4\|PE 18:1_20:3 | 3.22 | 2.91 | 3.20 | 3.11 | 2.75 | 2.29 | 2.40 | 2.47 | 2.86 | 3.34 | 2.93 | 3.03 | 3.26 | 3.36 | 4.26 | 3.63 |
| PE 38:5\|PE 16:0_22:5 | 1.86 | 1.78 | 2.21 | 1.95 | 2.76 | 2.19 | 2.21 | 2.37 | 5.41 | 6.18 | 5.77 | 5.77 | 9.75 | 9.66 | 10.99 | 10.13 |
| PE 38:5\|PE 18:1_20:4 | 1.73 | 1.74 | 2.15 | 1.87 | 2.58 | 2.05 | 2.63 | 2.41 | 5.88 | 6.39 | 6.73 | 6.32 | 10.11 | 9.78 | 9.28 | 9.73 |
| PE 38:6\|PE 16:0_22:6 | 9.00 | 7.59 | 6.01 | 7.54 | 10.94 | 9.56 | 10.74 | 10.38 | 35.62 | 40.26 | 38.48 | 38.01 | 36.70 | 38.71 | 35.28 | 36.89 |
| PE 38:7\|PE 16:1_22:6 | 0.11 | 0.07 | 0.09 | 0.09 | 0.13 | 0.13 | 0.12 | 0.13 | 0.23 | 0.29 | 0.27 | 0.26 | 0.43 | 0.43 | 0.41 | 0.42 |
| PE 39:0\|PE 18:0_21:0 | 0.05 | 0.06 | 0.08 | 0.07 | 0.03 | 0.02 | 0.02 | 0.02 | 0.05 | 0.06 | 0.07 | 0.06 | 0.04 | 0.04 | 0.05 | 0.04 |
| PE 39:1\|PE 21:0_18:1 | 0.35 | 0.36 | 0.30 | 0.34 | 0.24 | 0.22 | 0.31 | 0.26 | 0.50 | 0.61 | 0.51 | 0.54 | 0.52 | 0.41 | 0.41 | 0.45 |
| PE 39:3\|PE 21:1_18:2 | 0.14 | 0.14 | 0.14 | 0.14 | 0.16 | 0.13 | 0.15 | 0.15 | 0.17 | 0.16 | 0.16 | 0.16 | 0.14 | 0.14 | 0.12 | 0.13 |
| PE 39:5\|PE 17:0_22:5 | 0.13 | 0.12 | 0.15 | 0.13 | 0.25 | 0.20 | 0.21 | 0.22 | 0.55 | 0.62 | 0.47 | 0.55 | 0.80 | 0.83 | 0.99 | 0.87 |
| PE 39:6\|PE 17:0_22:6 | 0.11 | 0.11 | 0.12 | 0.12 | 0.23 | 0.20 | 0.26 | 0.23 | 0.77 | 0.89 | 0.90 | 0.85 | 0.94 | 0.92 | 1.12 | 0.99 |
| PE 40:1\|PE 22:0_18:1 | 0.50 | 0.58 | 0.48 | 0.52 | 0.34 | 0.28 | 0.32 | 0.31 | 0.54 | 0.65 | 0.51 | 0.56 | 0.51 | 0.37 | 0.45 | 0.44 |
| PE 40:2\|PE 18:1_22:1 | 2.08 | 1.31 | 1.54 | 1.65 | 0.35 | 0.33 | 0.37 | 0.35 | 0.65 | 0.88 | 0.66 | 0.72 | 0.69 | 0.75 | 0.75 | 0.73 |
| PE 40:3\|PE 18:1_22:2 | 2.69 | 2.18 | 2.27 | 2.38 | 0.93 | 0.79 | 0.73 | 0.81 | 0.71 | 0.82 | 0.74 | 0.76 | 0.46 | 0.54 | 0.61 | 0.54 |
| PE 40:4\|PE 18:1_22:3 | 2.51 | 2.44 | 2.35 | 2.44 | 3.04 | 2.61 | 3.61 | 3.07 | 2.91 | 3.20 | 3.84 | 3.31 | 5.07 | 5.34 | 5.41 | 5.27 |
| PE 40:5\|PE 18:1_22:4 | 1.23 | 1.12 | 1.12 | 1.15 | 1.76 | 1.41 | 1.67 | 1.60 | 2.90 | 3.30 | 2.61 | 2.93 | 6.63 | 6.80 | 6.20 | 6.54 |
| PE 40:6\|PE 18:0_22:6 | 1.03 | 0.75 | 0.89 | 0.88 | 1.16 | 1.03 | 0.94 | 1.04 | 2.17 | 2.48 | 2.12 | 2.25 | 4.58 | 4.19 | 4.23 | 4.33 |
| PE 40:6\|PE 18:1_22:5 | 2.85 | 2.55 | 2.43 | 2.61 | 8.76 | 7.17 | 7.18 | 7.66 | 18.05 | 20.06 | 24.17 | 20.71 | 25.99 | 26.87 | 22.81 | 25.22 |
| PE 40:7\|PE 18:1_22:6 | 1.58 | 1.05 | 1.37 | 1.31 | 1.72 | 1.61 | 1.49 | 1.61 | 2.78 | 3.52 | 2.90 | 3.04 | 5.75 | 5.34 | 4.41 | 5.17 |
| PE 40:8\|PE 18:2_22:6 | 0.01 | 0.02 | 0.01 | 0.01 | 0.01 | 0.01 | 0.01 | 0.01 | 0.03 | 0.03 | 0.03 | 0.03 | 0.02 | 0.02 | 0.02 | 0.02 |
| PE 41:1\|PE 23:0_18:1 | 0.04 | 0.04 | 0.04 | 0.04 | 0.02 | 0.02 | 0.02 | 0.02 | 0.04 | 0.05 | 0.06 | 0.05 | 0.05 | 0.04 | 0.04 | 0.04 |
| PE 43:7\|PE 21:1_22:6 | 0.06 | 0.05 | 0.06 | 0.06 | 0.03 | 0.03 | 0.03 | 0.03 | 0.06 | 0.07 | 0.05 | 0.06 | 0.04 | 0.04 | 0.04 | 0.04 |
| PE O-30:0\|PE O-18:0_12:0 | 0.21 | 0.23 | 0.20 | 0.21 | 0.41 | 0.36 | 0.41 | 0.39 | 0.23 | 0.23 | 0.25 | 0.24 | 0.48 | 0.49 | 0.63 | 0.53 |
| PE O-30:1\|PE O-18:1_12:0 | 0.07 | 0.07 | 0.07 | 0.07 | 0.04 | 0.04 | 0.05 | 0.04 | 0.03 | 0.03 | 0.03 | 0.03 | 0.03 | 0.03 | 0.03 | 0.03 |
| PE O-30:2\|PE O-18:2_12:0 | 0.05 | 0.06 | 0.06 | 0.06 | 0.04 | 0.03 | 0.03 | 0.03 | 0.03 | 0.03 | 0.03 | 0.03 | 0.03 | 0.03 | 0.03 | 0.03 |
| PE O-32:1\|PE O-16:1_16:0 | 0.17 | 0.19 | 0.20 | 0.19 | 0.18 | 0.16 | 0.19 | 0.18 | 0.45 | 0.46 | 0.50 | 0.47 | 0.51 | 0.54 | 0.51 | 0.52 |
| PE O-32:2\|PE O-14:1_18:1 | 0.09 | 0.08 | 0.06 | 0.08 | 0.10 | 0.09 | 0.08 | 0.09 | 0.26 | 0.27 | 0.25 | 0.26 | 0.37 | 0.40 | 0.41 | 0.39 |
| PE O-32:3\|PE O-14:1_18:2 | 0.47 | 0.40 | 0.48 | 0.45 | 0.21 | 0.20 | 0.20 | 0.20 | 0.09 | 0.10 | 0.08 | 0.09 | 0.09 | 0.09 | 0.08 | 0.08 |
| PE O-33:4\|PE O-15:1_18:3 | 0.09 | 0.07 | 0.09 | 0.09 | 0.10 | 0.09 | 0.09 | 0.09 | 0.06 | 0.07 | 0.07 | 0.06 | 0.09 | 0.09 | 0.11 | 0.09 |
| PE O-34:1\|PE O-16:0_18:1 | 1.58 | 1.73 | 1.65 | 1.65 | 1.32 | 1.07 | 1.22 | 1.20 | 1.04 | 1.16 | 0.93 | 1.04 | 1.00 | 0.96 | 1.17 | 1.05 |
| PE O-34:2\|PE O-16:1_18:1 | 12.08 | 11.20 | 11.19 | 11.49 | 8.92 | 7.33 | 7.30 | 7.81 | 6.11 | 6.81 | 7.74 | 6.87 | 5.41 | 5.35 | 5.55 | 5.44 |
| PE O-34:3\|PE O-16:1_18:2 | 7.18 | 6.57 | 5.70 | 6.48 | 6.64 | 5.50 | 6.48 | 6.17 | 1.59 | 1.74 | 1.45 | 1.59 | 1.88 | 1.93 | 1.81 | 1.87 |
| PE O-34:4\|PE O-16:1_18:3 | 0.63 | 0.63 | 0.62 | 0.63 | 0.79 | 0.67 | 0.62 | 0.69 | 0.10 | 0.10 | 0.08 | 0.09 | 0.14 | 0.15 | 0.16 | 0.15 |
| PE O-34:5\|PE O-14:1_20:4 | 0.15 | 0.15 | 0.18 | 0.16 | 0.11 | 0.10 | 0.10 | 0.10 | 0.11 | 0.13 | 0.14 | 0.12 | 0.09 | 0.10 | 0.11 | 0.10 |
| PE O-35:2\|PE O-17:1_18:1 | 1.93 | 1.89 | 1.51 | 1.78 | 1.75 | 1.46 | 1.98 | 1.72 | 1.08 | 1.21 | 1.39 | 1.22 | 1.05 | 1.12 | 1.00 | 1.05 |
| PE O-35:5\|PE O-15:1_20:4 | 0.15 | 0.13 | 0.11 | 0.13 | 0.15 | 0.13 | 0.13 | 0.13 | 0.14 | 0.16 | 0.18 | 0.16 | 0.14 | 0.15 | 0.16 | 0.15 |
| PE O-36:1\|PE O-16:1_20:0 | 0.20 | 0.25 | 0.29 | 0.25 | 0.19 | 0.15 | 0.19 | 0.18 | 0.31 | 0.35 | 0.38 | 0.34 | 0.23 | 0.19 | 0.19 | 0.20 |
| PE O-36:1\|PE O-18:0_18:1 | 2.58 | 2.43 | 2.44 | 2.49 | 1.49 | 1.31 | 1.32 | 1.37 | 0.95 | 1.01 | 1.15 | 1.03 | 0.76 | 0.76 | 0.75 | 0.76 |
| PE O-36:2\|PE O-18:1_18:1 | 14.50 | 12.06 | 13.72 | 13.44 | 8.31 | 7.32 | 9.75 | 8.43 | 4.71 | 5.46 | 6.56 | 5.56 | 4.29 | 4.33 | 4.51 | 4.38 |
| PE O-36:3\|PE O-18:2_18:1 | 19.27 | 17.75 | 17.24 | 18.09 | 13.73 | 11.32 | 13.93 | 12.93 | 5.61 | 6.55 | 6.21 | 6.10 | 7.45 | 7.89 | 6.79 | 7.37 |
| PE O-36:4\|PE O-18:2_18:2 | 5.58 | 5.23 | 6.03 | 5.62 | 4.67 | 4.14 | 3.69 | 4.16 | 1.39 | 1.61 | 1.48 | 1.49 | 2.43 | 2.54 | 3.20 | 2.72 |
| PE O-36:5\|PE O-16:1_20:4 | 1.75 | 1.63 | 1.53 | 1.64 | 1.33 | 1.10 | 1.45 | 1.28 | 1.27 | 1.50 | 1.33 | 1.36 | 1.01 | 1.01 | 1.12 | 1.05 |
| PE O-36:6\|PE O-16:1_20:5 | 0.61 | 0.56 | 0.69 | 0.62 | 0.62 | 0.54 | 0.67 | 0.61 | 1.53 | 1.71 | 1.80 | 1.68 | 0.80 | 0.83 | 0.83 | 0.82 |
| PE O-36:7\|PE O-14:1_22:6 | 0.06 | 0.06 | 0.05 | 0.06 | 0.10 | 0.09 | 0.11 | 0.10 | 0.18 | 0.21 | 0.16 | 0.18 | 0.18 | 0.19 | 0.23 | 0.20 |
| PE O-37:2\|PE O-19:1_18:1 | 1.86 | 1.97 | 1.86 | 1.89 | 2.71 | 2.34 | 3.03 | 2.69 | 3.15 | 3.08 | 3.32 | 3.19 | 2.85 | 2.95 | 3.24 | 3.01 |
| PE O-37:2\|PE O-21:2_16:0 | 1.07 | 0.82 | 0.66 | 0.85 | 0.35 | 0.33 | 0.36 | 0.34 | 0.61 | 0.68 | 0.72 | 0.67 | 0.44 | 0.46 | 0.56 | 0.48 |
| PE O-37:3\|PE O-19:2_18:1 | 1.19 | 1.03 | 1.15 | 1.12 | 1.82 | 1.61 | 1.77 | 1.73 | 1.38 | 1.66 | 1.73 | 1.59 | 1.36 | 1.51 | 1.46 | 1.44 |
| PE O-37:4\|PE O-19:2_18:2 | 0.27 | 0.19 | 0.23 | 0.23 | 0.49 | 0.41 | 0.40 | 0.43 | 0.32 | 0.38 | 0.33 | 0.34 | 0.43 | 0.48 | 0.39 | 0.43 |
| PE O-37:7\|PE O-15:1_22:6 | 0.14 | 0.13 | 0.11 | 0.13 | 0.20 | 0.18 | 0.21 | 0.19 | 0.52 | 0.57 | 0.62 | 0.57 | 0.43 | 0.46 | 0.53 | 0.47 |
| PE O-38:2\|PE O-18:1_20:1 | 1.19 | 1.13 | 1.37 | 1.23 | 0.73 | 0.66 | 0.60 | 0.66 | 0.94 | 1.10 | 1.21 | 1.08 | 0.95 | 0.93 | 1.05 | 0.98 |
| PE O-38:3\|PE O-18:2_20:1 | 1.79 | 1.67 | 1.51 | 1.66 | 1.21 | 1.02 | 1.29 | 1.17 | 1.10 | 1.26 | 1.21 | 1.19 | 1.34 | 1.34 | 1.55 | 1.41 |
| PE O-38:4\|PE O-18:1_20:3 | 2.05 | 1.84 | 2.14 | 2.01 | 1.49 | 1.25 | 1.56 | 1.42 | 0.80 | 0.93 | 1.07 | 0.93 | 1.14 | 1.25 | 1.10 | 1.16 |
| PE O-38:5\|PE O-18:1_20:4 | 2.55 | 2.46 | 3.07 | 2.70 | 2.24 | 1.95 | 2.49 | 2.22 | 2.38 | 2.63 | 2.45 | 2.48 | 3.26 | 3.27 | 3.40 | 3.31 |
| PE O-38:6\|PE O-16:1_22:5 | 2.56 | 2.46 | 2.06 | 2.36 | 3.33 | 2.88 | 4.04 | 3.40 | 5.13 | 6.01 | 5.75 | 5.61 | 6.12 | 6.44 | 7.62 | 6.72 |
| PE O-38:7\|PE O-16:1_22:6 | 1.30 | 1.26 | 1.60 | 1.39 | 2.28 | 1.95 | 2.44 | 2.21 | 5.71 | 6.47 | 7.31 | 6.48 | 5.27 | 5.20 | 4.45 | 4.98 |
| PE O-38:8\|PE O-16:2_22:6 | 0.10 | 0.10 | 0.12 | 0.11 | 0.17 | 0.15 | 0.15 | 0.15 | 0.37 | 0.43 | 0.32 | 0.38 | 0.44 | 0.47 | 0.53 | 0.48 |
| PE O-39:7\|PE O-17:1_22:6 | 0.15 | 0.14 | 0.16 | 0.15 | 0.25 | 0.20 | 0.18 | 0.21 | 0.59 | 0.68 | 0.82 | 0.70 | 0.63 | 0.67 | 0.66 | 0.65 |
| PE O-40:3\|PE O-18:2_22:1 | 0.76 | 0.70 | 0.87 | 0.78 | 0.32 | 0.27 | 0.34 | 0.31 | 0.35 | 0.37 | 0.31 | 0.34 | 0.46 | 0.44 | 0.49 | 0.46 |
| PE O-40:4\|PE O-18:1_22:3 | 1.45 | 1.22 | 1.24 | 1.30 | 0.53 | 0.54 | 0.69 | 0.59 | 0.40 | 0.51 | 0.45 | 0.45 | 0.36 | 0.43 | 0.47 | 0.42 |
| PE O-40:5\|PE O-18:1_22:4 | 2.37 | 2.14 | 2.08 | 2.20 | 2.08 | 1.77 | 1.82 | 1.88 | 1.48 | 1.71 | 1.45 | 1.54 | 2.81 | 3.21 | 4.09 | 3.37 |
| PE O-40:6\|PE O-18:1_22:5 | 1.55 | 1.57 | 1.48 | 1.53 | 2.17 | 1.74 | 2.15 | 2.01 | 2.73 | 3.33 | 2.52 | 2.85 | 3.13 | 3.14 | 2.76 | 3.01 |
| PE O-40:7\|PE O-18:1_22:6 | 1.41 | 1.22 | 0.99 | 1.21 | 2.30 | 1.92 | 1.83 | 2.00 | 4.69 | 5.65 | 4.23 | 4.84 | 10.19 | 10.23 | 8.86 | 9.76 |
| PE O-40:7\|PE O-18:2_22:5 | 1.35 | 1.22 | 1.10 | 1.22 | 2.30 | 2.08 | 2.88 | 2.41 | 4.89 | 5.65 | 6.76 | 5.75 | 10.19 | 10.23 | 8.37 | 9.60 |
| PE O-40:8\|PE O-18:2_22:6 | 0.92 | 0.88 | 0.77 | 0.86 | 1.38 | 1.12 | 0.97 | 1.15 | 3.74 | 4.33 | 3.54 | 3.86 | 4.83 | 4.91 | 6.30 | 5.35 |
| PE O-40:9\|PE O-18:3_22:6 | 0.33 | 0.34 | 0.40 | 0.36 | 0.50 | 0.43 | 0.56 | 0.49 | 1.11 | 1.26 | 1.28 | 1.21 | 1.64 | 1.72 | 1.60 | 1.65 |
| PE P-34:1\|PE P-16:0_18:1 | 0.80 | 0.58 | 0.60 | 0.65 | 0.31 | 0.28 | 0.24 | 0.28 | 0.18 | 0.22 | 0.17 | 0.19 | 0.18 | 0.17 | 0.20 | 0.18 |
| PE P-34:2\|PE P-16:0_18:2 | 1.29 | 1.33 | 1.28 | 1.30 | 0.68 | 0.62 | 0.35 | 0.55 | 0.26 | 0.25 | 0.18 | 0.23 | 0.24 | 0.24 | 0.15 | 0.21 |
| PE P-35:1\|PE P-17:0_18:1 | 0.20 | 0.13 | 0.12 | 0.15 | 0.08 | 0.07 | 0.07 | 0.08 | 0.05 | 0.06 | 0.05 | 0.05 | 0.08 | 0.08 | 0.06 | 0.07 |
| PE P-35:2\|PE P-17:0_18:2 | 0.19 | 0.20 | 0.18 | 0.19 | 0.10 | 0.10 | 0.08 | 0.09 | 0.06 | 0.06 | 0.03 | 0.05 | 0.07 | 0.07 | 0.07 | 0.07 |
| PE P-36:1\|PE P-18:0_18:1 | 0.41 | 0.34 | 0.48 | 0.41 | 0.18 | 0.15 | 0.17 | 0.17 | 0.08 | 0.08 | 0.09 | 0.08 | 0.19 | 0.21 | 0.20 | 0.20 |
| PE P-36:2\|PE P-18:1_18:1 | 1.06 | 0.78 | 1.02 | 0.94 | 0.34 | 0.27 | 0.32 | 0.31 | 0.12 | 0.18 | 0.14 | 0.15 | 0.17 | 0.15 | 0.15 | 0.16 |
| PE P-36:3\|PE P-18:1_18:2 | 1.06 | 0.78 | 0.92 | 0.91 | 0.46 | 0.41 | 0.35 | 0.41 | 0.17 | 0.21 | 0.22 | 0.20 | 0.32 | 0.29 | 0.29 | 0.30 |
| PE P-36:4\|PE P-18:2_18:2 | 0.34 | 0.25 | 0.25 | 0.28 | 0.16 | 0.15 | 0.15 | 0.15 | 0.15 | 0.16 | 0.12 | 0.14 | 0.16 | 0.13 | 0.15 | 0.15 |
| PE P-36:5\|PE P-16:0_20:5 | 0.09 | 0.11 | 0.07 | 0.09 | 0.09 | 0.09 | 0.10 | 0.10 | 0.22 | 0.24 | 0.23 | 0.23 | 0.14 | 0.16 | 0.17 | 0.16 |
| PE P-36:6\|PE P-14:0_22:6 | 0.04 | 0.02 | 0.02 | 0.03 | 0.03 | 0.03 | 0.04 | 0.03 | 0.05 | 0.06 | 0.05 | 0.05 | 0.06 | 0.06 | 0.08 | 0.07 |
| PE P-38:4\|PE P-16:0_22:4 | 0.07 | 0.06 | 0.07 | 0.07 | 0.05 | 0.05 | 0.06 | 0.05 | 0.06 | 0.08 | 0.06 | 0.06 | 0.08 | 0.08 | 0.09 | 0.08 |
| PE P-38:5\|PE P-16:0_22:5 | 0.56 | 0.42 | 0.43 | 0.46 | 0.39 | 0.34 | 0.45 | 0.39 | 0.52 | 0.64 | 0.65 | 0.60 | 0.76 | 0.68 | 0.71 | 0.72 |
| PE P-39:6\|PE P-17:0_22:6 | 0.04 | 0.05 | 0.05 | 0.05 | 0.04 | 0.04 | 0.03 | 0.04 | 0.13 | 0.13 | 0.08 | 0.11 | 0.12 | 0.13 | 0.13 | 0.13 |
| PE P-40:6\|PE P-18:1_22:5 | 0.19 | 0.14 | 0.20 | 0.17 | 0.19 | 0.15 | 0.15 | 0.16 | 0.28 | 0.29 | 0.34 | 0.30 | 0.66 | 0.57 | 0.71 | 0.65 |
| PE P-40:7\|PE P-18:1_22:6 | 0.25 | 0.26 | 0.14 | 0.22 | 0.22 | 0.22 | 0.18 | 0.21 | 0.76 | 0.78 | 0.68 | 0.74 | 0.85 | 0.88 | 0.81 | 0.84 |
| PE P-40:8\|PE P-18:2_22:6 | 0.05 | 0.06 | 0.03 | 0.05 | 0.06 | 0.06 | 0.07 | 0.07 | 0.14 | 0.15 | 0.07 | 0.12 | 0.18 | 0.21 | 0.16 | 0.18 |
| PG 30:0\|PG 14:0_16:0 | 0.01 | 0.00 | 0.01 | 0.01 | 0.01 | 0.01 | 0.01 | 0.01 | 0.01 | 0.01 | 0.01 | 0.01 | 0.01 | 0.01 | 0.01 | 0.01 |
| PG 30:1\|PG 12:0_18:1 | 0.00 | 0.00 | 0.00 | 0.00 | 0.01 | 0.01 | 0.01 | 0.01 | 0.01 | 0.01 | 0.01 | 0.01 | 0.01 | 0.01 | 0.01 | 0.01 |
| PG 32:1\|PG 14:0_18:1 | 0.02 | 0.02 | 0.01 | 0.02 | 0.03 | 0.03 | 0.03 | 0.03 | 0.03 | 0.02 | 0.02 | 0.02 | 0.02 | 0.02 | 0.01 | 0.02 |
| PG 32:2\|PG 14:0_18:2 | 0.01 | 0.00 | 0.00 | 0.00 | 0.02 | 0.02 | 0.02 | 0.02 | 0.01 | 0.01 | 0.01 | 0.01 | 0.01 | 0.01 | 0.01 | 0.01 |
| PG 34:1\|PG 16:0_18:1 | 0.06 | 0.06 | 0.05 | 0.06 | 0.16 | 0.16 | 0.17 | 0.16 | 0.29 | 0.20 | 0.29 | 0.26 | 0.30 | 0.28 | 0.31 | 0.30 |
| PG 34:3\|PG 16:1_18:2 | 0.02 | 0.01 | 0.01 | 0.01 | 0.03 | 0.03 | 0.03 | 0.03 | 0.02 | 0.02 | 0.02 | 0.02 | 0.01 | 0.01 | 0.01 | 0.01 |
| PG 34:4\|PG 14:0_20:4 | 0.00 | 0.00 | 0.00 | 0.00 | 0.01 | 0.01 | 0.01 | 0.01 | 0.01 | 0.01 | 0.01 | 0.01 | 0.01 | 0.01 | 0.01 | 0.01 |
| PG 35:3\|PG 17:1_18:2 | 0.04 | 0.05 | 0.05 | 0.05 | 0.11 | 0.10 | 0.09 | 0.10 | 0.06 | 0.06 | 0.09 | 0.07 | 0.02 | 0.02 | 0.02 | 0.02 |
| PG 36:1\|PG 18:0_18:1 | 0.07 | 0.07 | 0.07 | 0.07 | 0.14 | 0.16 | 0.18 | 0.16 | 0.22 | 0.15 | 0.18 | 0.18 | 0.16 | 0.14 | 0.14 | 0.15 |
| PG 36:3\|PG 18:1_18:2 | 0.06 | 0.05 | 0.05 | 0.05 | 0.12 | 0.13 | 0.16 | 0.14 | 0.13 | 0.09 | 0.10 | 0.11 | 0.13 | 0.12 | 0.10 | 0.11 |
| PG 36:4\|PG 18:2_18:2 | 0.02 | 0.02 | 0.02 | 0.02 | 0.03 | 0.04 | 0.04 | 0.03 | 0.04 | 0.02 | 0.03 | 0.03 | 0.03 | 0.03 | 0.03 | 0.03 |
| PG 36:6\|PG 14:0_22:6 | 0.00 | 0.00 | 0.00 | 0.00 | 0.01 | 0.01 | 0.01 | 0.01 | 0.02 | 0.01 | 0.02 | 0.01 | 0.01 | 0.01 | 0.02 | 0.02 |
| PG 38:3\|PG 18:1_20:2 | 0.07 | 0.05 | 0.04 | 0.05 | 0.12 | 0.13 | 0.16 | 0.14 | 0.06 | 0.07 | 0.09 | 0.08 | 0.04 | 0.04 | 0.04 | 0.04 |
| PG 38:6\|PG 16:0_22:6 | 0.01 | 0.01 | 0.02 | 0.01 | 0.04 | 0.04 | 0.04 | 0.04 | 0.06 | 0.04 | 0.04 | 0.04 | 0.04 | 0.04 | 0.05 | 0.04 |
| PG 40:4\|PG 18:0_22:4 | 0.03 | 0.02 | 0.02 | 0.03 | 0.06 | 0.06 | 0.06 | 0.06 | 0.05 | 0.04 | 0.05 | 0.05 | 0.03 | 0.03 | 0.04 | 0.04 |
| PG 40:8\|PG 18:2_22:6 | 0.00 | 0.00 | 0.00 | 0.00 | 0.01 | 0.01 | 0.01 | 0.01 | 0.02 | 0.01 | 0.01 | 0.01 | 0.01 | 0.01 | 0.02 | 0.01 |
| PG 42:10\|PG 20:4_22:6 | 0.00 | 0.00 | 0.00 | 0.00 | 0.01 | 0.01 | 0.01 | 0.01 | 0.01 | 0.01 | 0.01 | 0.01 | 0.01 | 0.01 | 0.02 | 0.01 |
| PG 44:11\|PG 22:5_22:6 | 0.00 | 0.00 | 0.00 | 0.00 | 0.01 | 0.01 | 0.01 | 0.01 | 0.03 | 0.02 | 0.03 | 0.02 | 0.02 | 0.02 | 0.02 | 0.02 |
| PI 28:0\|PI 12:0_16:0 | 0.01 | 0.01 | 0.01 | 0.01 | 0.01 | 0.02 | 0.01 | 0.01 | 0.02 | 0.02 | 0.02 | 0.02 | 0.02 | 0.02 | 0.02 | 0.02 |
| PI 28:1PI 10:0_18:1 | 0.00 | 0.00 | 0.00 | 0.00 | 0.01 | 0.01 | 0.01 | 0.01 | 0.01 | 0.01 | 0.01 | 0.01 | 0.02 | 0.01 | 0.02 | 0.02 |
| PI 29:0\|PI 14:0_15:0 | 0.02 | 0.01 | 0.01 | 0.01 | 0.03 | 0.03 | 0.02 | 0.03 | 0.03 | 0.03 | 0.03 | 0.03 | 0.05 | 0.04 | 0.03 | 0.04 |
| PI 30:0\|PI 14:0_16:0 | 0.03 | 0.02 | 0.02 | 0.03 | 0.07 | 0.07 | 0.07 | 0.07 | 0.06 | 0.07 | 0.05 | 0.06 | 0.07 | 0.05 | 0.05 | 0.06 |
| PI 30:1\|PI 12:0_18:1 | 0.02 | 0.02 | 0.02 | 0.02 | 0.05 | 0.05 | 0.05 | 0.05 | 0.05 | 0.06 | 0.05 | 0.06 | 0.11 | 0.07 | 0.08 | 0.09 |
| PI 31:0\|PI 15:0_16:0 | 0.09 | 0.08 | 0.07 | 0.08 | 0.09 | 0.10 | 0.09 | 0.09 | 0.11 | 0.11 | 0.12 | 0.12 | 0.10 | 0.09 | 0.09 | 0.09 |
| PI 31:0\|PI 15:0_16:1 | 0.04 | 0.04 | 0.04 | 0.04 | 0.03 | 0.03 | 0.03 | 0.03 | 0.07 | 0.07 | 0.08 | 0.07 | 0.07 | 0.07 | 0.06 | 0.06 |
| PI 32:1\|PI 14:0_18:1 | 0.28 | 0.26 | 0.29 | 0.28 | 0.36 | 0.40 | 0.47 | 0.41 | 0.49 | 0.59 | 0.45 | 0.50 | 0.50 | 0.38 | 0.52 | 0.46 |
| PI 32:2\|PI 14:0_18:2 | 0.04 | 0.03 | 0.03 | 0.03 | 0.09 | 0.10 | 0.09 | 0.09 | 0.09 | 0.11 | 0.09 | 0.09 | 0.22 | 0.14 | 0.15 | 0.17 |
| PI 33:1\|PI 15:0_18:1 | 0.23 | 0.20 | 0.20 | 0.21 | 0.40 | 0.43 | 0.49 | 0.44 | 0.43 | 0.52 | 0.39 | 0.44 | 0.56 | 0.43 | 0.55 | 0.51 |
| PI 33:2\|PI 16:1_17:1 | 0.05 | 0.05 | 0.03 | 0.04 | 0.04 | 0.04 | 0.04 | 0.04 | 0.07 | 0.09 | 0.08 | 0.08 | 0.10 | 0.07 | 0.08 | 0.08 |
| PI 34:1\|PI 16:0_18:1 | 5.87 | 5.48 | 3.97 | 5.13 | 8.25 | 8.52 | 7.99 | 8.25 | 10.73 | 12.29 | 12.61 | 11.82 | 10.93 | 8.02 | 8.56 | 9.08 |
| PI 34:2\|PI 16:0_18:2 | 7.73 | 5.96 | 5.57 | 6.50 | 10.25 | 11.57 | 9.66 | 10.49 | 9.54 | 10.51 | 7.88 | 9.27 | 9.91 | 7.14 | 6.96 | 7.91 |
| PI 34:3\|PI 16:1_18:2 | 0.10 | 0.08 | 0.08 | 0.09 | 0.57 | 0.58 | 0.60 | 0.58 | 0.28 | 0.34 | 0.25 | 0.28 | 0.65 | 0.53 | 0.69 | 0.62 |
| PI 34:4\|PI 14:0_20:4 | 0.01 | 0.01 | 0.01 | 0.01 | 0.04 | 0.04 | 0.04 | 0.04 | 0.05 | 0.05 | 0.05 | 0.05 | 0.07 | 0.06 | 0.06 | 0.06 |
| PI 35:0\|PI 17:0_18:0 | 0.04 | 0.04 | 0.04 | 0.04 | 0.08 | 0.08 | 0.08 | 0.08 | 0.11 | 0.12 | 0.09 | 0.10 | 0.12 | 0.05 | 0.06 | 0.08 |
| PI 35:1\|PI 17:0_18:1 | 0.24 | 0.19 | 0.16 | 0.20 | 0.36 | 0.36 | 0.38 | 0.37 | 0.48 | 0.57 | 0.46 | 0.50 | 0.50 | 0.37 | 0.47 | 0.44 |
| PI 35:2\|PI 17:1_18:1 | 0.26 | 0.22 | 0.18 | 0.22 | 0.33 | 0.37 | 0.29 | 0.33 | 0.29 | 0.33 | 0.25 | 0.29 | 0.34 | 0.23 | 0.27 | 0.28 |
| PI 36:1\|PI 18:0_18:1 | 5.99 | 3.93 | 4.30 | 4.83 | 4.14 | 4.43 | 3.78 | 4.12 | 5.96 | 6.54 | 5.12 | 5.85 | 5.69 | 4.01 | 4.25 | 4.60 |
| PI 36:2\|PI 18:1_18:1 | 9.46 | 6.32 | 4.97 | 7.06 | 10.55 | 10.66 | 12.34 | 11.19 | 9.57 | 10.86 | 8.24 | 9.51 | 10.46 | 7.96 | 11.31 | 9.83 |
| PI 36:3\|PI 18:1_18:2 | 21.13 | 16.31 | 16.36 | 18.15 | 26.40 | 30.00 | 37.25 | 31.21 | 15.75 | 16.96 | 17.63 | 16.73 | 18.97 | 14.31 | 18.57 | 17.13 |
| PI 36:4\|PI 18:2_18:2 | 1.87 | 1.18 | 1.30 | 1.48 | 1.70 | 1.91 | 1.56 | 1.72 | 3.42 | 3.50 | 2.83 | 3.25 | 3.66 | 2.91 | 3.34 | 3.28 |
| PI 36:5\|PI 16:0_20:5 | 0.07 | 0.04 | 0.05 | 0.05 | 0.33 | 0.36 | 0.43 | 0.37 | 0.81 | 0.88 | 0.75 | 0.81 | 0.88 | 0.59 | 0.69 | 0.71 |
| PI 37:1\|PI 19:0_18:1 | 0.10 | 0.08 | 0.07 | 0.09 | 0.09 | 0.10 | 0.10 | 0.10 | 0.14 | 0.18 | 0.15 | 0.16 | 0.15 | 0.09 | 0.08 | 0.11 |
| PI 37:2\|PI 19:0_18:2 | 0.19 | 0.16 | 0.13 | 0.16 | 0.24 | 0.26 | 0.21 | 0.24 | 0.31 | 0.34 | 0.35 | 0.33 | 0.31 | 0.24 | 0.31 | 0.29 |
| PI 37:3\|PI 19:1_18:2 | 0.05 | 0.05 | 0.05 | 0.05 | 0.03 | 0.03 | 0.03 | 0.03 | 0.03 | 0.04 | 0.03 | 0.03 | 0.02 | 0.02 | 0.03 | 0.03 |
| PI 37:4\|PI 17:0_20:4 | 0.16 | 0.13 | 0.12 | 0.14 | 0.13 | 0.15 | 0.15 | 0.14 | 0.25 | 0.30 | 0.22 | 0.26 | 0.21 | 0.16 | 0.22 | 0.19 |
| PI 37:5\|PI 17:0_20:5 | 0.03 | 0.02 | 0.02 | 0.03 | 0.04 | 0.04 | 0.04 | 0.04 | 0.10 | 0.11 | 0.10 | 0.10 | 0.08 | 0.07 | 0.09 | 0.08 |
| PI 38:1\|PI 18:0_20:1 | 0.08 | 0.08 | 0.07 | 0.08 | 0.05 | 0.06 | 0.06 | 0.05 | 0.11 | 0.13 | 0.11 | 0.12 | 0.12 | 0.07 | 0.09 | 0.09 |
| PI 38:1\|PI 19:0_19:1 | 0.03 | 0.03 | 0.03 | 0.03 | 0.02 | 0.01 | 0.01 | 0.01 | 0.01 | 0.01 | 0.01 | 0.01 | 0.01 | 0.01 | 0.01 | 0.01 |
| PI 38:2\|PI 18:0_20:2 | 0.29 | 0.26 | 0.27 | 0.28 | 0.31 | 0.33 | 0.34 | 0.32 | 0.47 | 0.57 | 0.45 | 0.49 | 0.58 | 0.41 | 0.55 | 0.51 |
| PI 38:3\|PI 18:0_20:3 | 0.99 | 0.71 | 0.72 | 0.82 | 0.49 | 0.50 | 0.41 | 0.47 | 0.83 | 0.93 | 1.01 | 0.92 | 0.64 | 0.48 | 0.67 | 0.59 |
| PI 38:4\|PI 18:1_20:3 | 1.69 | 1.20 | 1.19 | 1.38 | 1.51 | 1.58 | 1.32 | 1.47 | 2.63 | 2.97 | 2.64 | 2.73 | 2.18 | 2.09 | 2.43 | 2.23 |
| PI 38:5\|PI 18:1_20:4 | 7.66 | 5.88 | 5.16 | 6.32 | 8.64 | 9.61 | 10.06 | 9.44 | 19.45 | 19.62 | 20.12 | 19.72 | 18.53 | 16.14 | 15.65 | 16.70 |
| PI 38:6\|PI 18:1_20:5 | 0.36 | 0.24 | 0.25 | 0.29 | 1.80 | 1.98 | 2.06 | 1.95 | 4.97 | 5.72 | 4.13 | 4.91 | 6.85 | 4.85 | 6.90 | 6.14 |
| PI 38:7\|PI 18:2_20:5 | 0.03 | 0.03 | 0.03 | 0.03 | 0.09 | 0.10 | 0.12 | 0.11 | 0.48 | 0.44 | 0.41 | 0.44 | 0.37 | 0.54 | 0.47 | 0.45 |
| PI 39:3\|PI 19:0_20:3 | 0.05 | 0.05 | 0.05 | 0.05 | 0.04 | 0.04 | 0.04 | 0.04 | 0.06 | 0.07 | 0.06 | 0.06 | 0.05 | 0.04 | 0.04 | 0.04 |
| PI 39:4\|PI 19:0_20:4 | 0.03 | 0.02 | 0.02 | 0.02 | 0.03 | 0.03 | 0.03 | 0.03 | 0.06 | 0.08 | 0.09 | 0.08 | 0.06 | 0.05 | 0.07 | 0.06 |
| PI 39:6\|PI 17:0_22:6 | 0.03 | 0.02 | 0.03 | 0.03 | 0.05 | 0.05 | 0.06 | 0.05 | 0.11 | 0.12 | 0.08 | 0.10 | 0.09 | 0.08 | 0.08 | 0.08 |
| PI 40:3\|PI 18:0_22:3 | 0.15 | 0.13 | 0.14 | 0.14 | 0.14 | 0.11 | 0.13 | 0.13 | 0.16 | 0.19 | 0.14 | 0.16 | 0.14 | 0.10 | 0.09 | 0.11 |
| PI 40:4\|PI 18:0_22:4 | 0.13 | 0.12 | 0.13 | 0.12 | 0.22 | 0.24 | 0.25 | 0.24 | 0.28 | 0.34 | 0.37 | 0.33 | 0.27 | 0.19 | 0.27 | 0.24 |
| PI 40:6\|PI 18:1_22:5 | 0.47 | 0.43 | 0.32 | 0.41 | 1.18 | 1.29 | 1.26 | 1.24 | 2.59 | 2.99 | 3.41 | 2.98 | 4.00 | 2.86 | 2.75 | 3.16 |
| PI 40:7\|PI 18:1_22:6 | 0.48 | 0.42 | 0.37 | 0.42 | 1.51 | 1.67 | 1.50 | 1.56 | 3.73 | 3.87 | 3.76 | 3.78 | 4.06 | 3.48 | 3.96 | 3.81 |
| PI 40:8\|PI 18:2_22:6 | 0.00 | 0.00 | 0.00 | 0.00 | 0.34 | 0.35 | 0.29 | 0.33 | 0.39 | 0.46 | 0.40 | 0.41 | 0.85 | 0.58 | 0.74 | 0.72 |
| PS 34:1\|PS 16:0_18:1 | 0.29 | 0.30 | 0.27 | 0.29 | 0.33 | 0.28 | 0.26 | 0.29 | 0.85 | 0.93 | 0.94 | 0.91 | 0.74 | 0.67 | 0.78 | 0.73 |
| PS 34:2\|PS 16:0_18:2 | 0.53 | 0.53 | 0.53 | 0.53 | 0.54 | 0.48 | 0.59 | 0.54 | 0.32 | 0.39 | 0.39 | 0.37 | 0.45 | 0.38 | 0.40 | 0.41 |
| PS 35:0\|PS 17:0_18:0 | 0.12 | 0.10 | 0.10 | 0.11 | 0.07 | 0.06 | 0.09 | 0.07 | 0.14 | 0.13 | 0.11 | 0.13 | 0.17 | 0.15 | 0.14 | 0.16 |
| PS 35:1\|PS 17:0_18:1 | 0.46 | 0.45 | 0.52 | 0.48 | 0.47 | 0.38 | 0.47 | 0.44 | 0.79 | 0.84 | 0.86 | 0.83 | 0.81 | 0.76 | 0.65 | 0.74 |
| PS 35:2\|PS 17:1_18:1 | 0.36 | 0.35 | 0.42 | 0.38 | 0.36 | 0.38 | 0.49 | 0.41 | 0.26 | 0.25 | 0.26 | 0.25 | 0.33 | 0.23 | 0.24 | 0.26 |
| PS 36:1\|PS 18:0_18:1 | 16.19 | 13.63 | 11.81 | 13.89 | 15.06 | 12.77 | 15.66 | 14.43 | 19.02 | 19.52 | 23.34 | 20.62 | 17.61 | 13.95 | 16.03 | 15.88 |
| PS 36:2\|PS 18:1_18:1 | 17.65 | 13.56 | 11.19 | 14.16 | 18.50 | 15.77 | 16.81 | 16.95 | 14.03 | 15.13 | 17.32 | 15.47 | 17.20 | 14.91 | 17.95 | 16.69 |
| PS 36:3\|PS 18:1_18:2 | 4.63 | 3.56 | 4.20 | 4.14 | 6.37 | 5.54 | 5.29 | 5.71 | 2.13 | 2.38 | 2.71 | 2.40 | 3.77 | 3.27 | 3.53 | 3.53 |
| PS 36:4\|PS 18:2_18:2 | 0.74 | 0.68 | 0.58 | 0.67 | 1.67 | 1.50 | 2.04 | 1.73 | 0.33 | 0.35 | 0.32 | 0.33 | 0.68 | 0.68 | 0.76 | 0.70 |
| PS 36:5\|PS 18:2_18:3 | 0.03 | 0.02 | 0.02 | 0.02 | 0.12 | 0.11 | 0.13 | 0.12 | 0.04 | 0.05 | 0.04 | 0.04 | 0.13 | 0.13 | 0.11 | 0.13 |
| PS 37:1\|PS 19:0_18:1 | 0.38 | 0.29 | 0.26 | 0.31 | 0.24 | 0.19 | 0.18 | 0.20 | 0.44 | 0.53 | 0.47 | 0.48 | 0.30 | 0.22 | 0.27 | 0.26 |
| PS 37:2\|PS 18:1_19:1 | 0.16 | 0.16 | 0.18 | 0.17 | 0.16 | 0.17 | 0.17 | 0.17 | 0.20 | 0.20 | 0.18 | 0.19 | 0.17 | 0.11 | 0.16 | 0.14 |
| PS 38:1\|PS 20:0_18:1 | 1.75 | 1.62 | 1.97 | 1.78 | 2.09 | 1.87 | 2.18 | 2.04 | 1.61 | 1.81 | 1.82 | 1.74 | 3.14 | 3.21 | 3.17 | 3.17 |
| PS 38:2\|PS 18:1_20:1 | 5.16 | 7.57 | 6.20 | 6.30 | 3.20 | 2.97 | 3.64 | 3.27 | 2.77 | 2.88 | 2.82 | 2.82 | 2.13 | 2.86 | 3.00 | 2.66 |
| PS 38:3\|PS 18:0_20:3 | 0.43 | 0.42 | 0.42 | 0.42 | 0.29 | 0.30 | 0.26 | 0.28 | 0.24 | 0.26 | 0.32 | 0.27 | 0.30 | 0.19 | 0.30 | 0.26 |
| PS 38:5\|PS 18:1_20:4 | 0.30 | 0.32 | 0.25 | 0.29 | 0.32 | 0.36 | 0.37 | 0.35 | 0.45 | 0.43 | 0.42 | 0.44 | 0.66 | 0.44 | 0.63 | 0.56 |
| PS 38:6\|PS 16:0_22:6 | 0.16 | 0.17 | 0.19 | 0.17 | 0.27 | 0.23 | 0.22 | 0.24 | 0.60 | 0.75 | 0.85 | 0.73 | 0.66 | 0.54 | 0.63 | 0.61 |
| PS 39:1\|PS 21:0_18:1 | 0.10 | 0.11 | 0.11 | 0.11 | 0.11 | 0.10 | 0.09 | 0.10 | 0.12 | 0.14 | 0.14 | 0.14 | 0.13 | 0.14 | 0.17 | 0.15 |
| PS 39:5\|PS 17:0_22:5 | 0.19 | 0.19 | 0.18 | 0.19 | 0.23 | 0.27 | 0.21 | 0.24 | 0.45 | 0.43 | 0.52 | 0.47 | 0.61 | 0.43 | 0.60 | 0.53 |
| PS 39:6\|PS 17:0_22:6 | 0.09 | 0.10 | 0.11 | 0.10 | 0.16 | 0.14 | 0.17 | 0.16 | 0.38 | 0.43 | 0.51 | 0.44 | 0.53 | 0.45 | 0.43 | 0.47 |
| PS 40:4\|PS 18:0_22:4 | 3.52 | 3.08 | 3.37 | 3.33 | 2.81 | 2.34 | 3.28 | 2.80 | 2.90 | 3.22 | 3.36 | 3.15 | 4.24 | 3.56 | 4.00 | 3.94 |
| PS 40:5\|PS 18:0_22:5 | 7.44 | 6.01 | 7.22 | 6.90 | 8.73 | 7.41 | 10.01 | 8.68 | 13.73 | 14.97 | 14.59 | 14.40 | 18.31 | 15.52 | 13.65 | 15.84 |
| PS 40:6\|PS 18:0_22:6 | 5.41 | 4.34 | 3.44 | 4.40 | 8.49 | 7.31 | 9.20 | 8.30 | 14.58 | 15.54 | 16.56 | 15.54 | 18.57 | 19.75 | 16.86 | 18.39 |
| PS 40:7\|PS 18:1_22:6 | 2.95 | 2.52 | 2.35 | 2.61 | 8.90 | 8.33 | 9.04 | 8.74 | 12.82 | 13.74 | 10.96 | 12.49 | 18.19 | 15.82 | 19.31 | 17.78 |
| PS 40:8\|PS 18:2_22:6 | 0.15 | 0.17 | 0.21 | 0.17 | 1.28 | 1.36 | 1.74 | 1.46 | 1.41 | 1.43 | 1.26 | 1.36 | 2.78 | 1.82 | 2.53 | 2.31 |
| PS 44:12\|PS 22:6_22:6 | 0.06 | 0.05 | 0.05 | 0.06 | 0.17 | 0.17 | 0.17 | 0.17 | 0.48 | 0.48 | 0.39 | 0.45 | 0.30 | 0.21 | 0.22 | 0.23 |
| SHexCer 42:2;2O | 0.22 | 0.24 | 0.22 | 0.22 | 0.24 | 0.23 | 0.25 | 0.24 | 0.37 | 0.45 | 0.34 | 0.38 | 0.25 | 0.19 | 0.21 | 0.22 |
| SHexCer 42:3;2O | 0.30 | 0.20 | 0.21 | 0.23 | 0.39 | 0.38 | 0.50 | 0.42 | 0.62 | 0.57 | 0.42 | 0.52 | 0.47 | 0.44 | 0.45 | 0.45 |
| SHexCer 43:4;3O | 0.08 | 0.06 | 0.08 | 0.07 | 0.18 | 0.20 | 0.23 | 0.20 | 0.27 | 0.26 | 0.18 | 0.23 | 0.19 | 0.17 | 0.18 | 0.18 |
| SHexCer 45:2;2O | 0.04 | 0.04 | 0.04 | 0.04 | 0.10 | 0.10 | 0.13 | 0.11 | 0.17 | 0.14 | 0.10 | 0.13 | 0.13 | 0.08 | 0.10 | 0.10 |
| SHexCer 46:3;3O | 0.05 | 0.05 | 0.05 | 0.05 | 0.09 | 0.09 | 0.12 | 0.10 | 0.13 | 0.12 | 0.07 | 0.10 | 0.09 | 0.08 | 0.07 | 0.08 |
| SHexCer 48:3;3O | 0.05 | 0.04 | 0.05 | 0.05 | 0.10 | 0.09 | 0.09 | 0.09 | 0.14 | 0.14 | 0.10 | 0.12 | 0.11 | 0.10 | 0.09 | 0.10 |
| SM 27:1;2O\|SM 15:1;2O/12:0 | 0.01 | 0.01 | 0.01 | 0.01 | 0.01 | 0.01 | 0.01 | 0.01 | 0.01 | 0.01 | 0.01 | 0.01 | 0.01 | 0.01 | 0.01 | 0.01 |
| SM 28:0;2O\|SM 17:0;2O/11:0 | 0.16 | 0.09 | 0.12 | 0.12 | 0.07 | 0.07 | 0.09 | 0.08 | 0.12 | 0.13 | 0.12 | 0.12 | 0.03 | 0.04 | 0.04 | 0.04 |
| SM 28:1;2O\|SM 10:0;2O/18:1 | 0.20 | 0.14 | 0.17 | 0.17 | 0.13 | 0.15 | 0.15 | 0.15 | 0.16 | 0.16 | 0.16 | 0.16 | 0.07 | 0.07 | 0.06 | 0.07 |
| SM 28:2;2O\|SM 8:1;2O/20:1 | 0.07 | 0.04 | 0.04 | 0.05 | 0.04 | 0.04 | 0.04 | 0.04 | 0.06 | 0.07 | 0.07 | 0.07 | 0.03 | 0.03 | 0.03 | 0.03 |
| SM 31:1;2O\|SM 17:1;2O/14:0 | 0.15 | 0.10 | 0.09 | 0.11 | 0.07 | 0.07 | 0.06 | 0.07 | 0.07 | 0.08 | 0.10 | 0.08 | 0.06 | 0.07 | 0.07 | 0.07 |
| SM 32:1;2O\|SM 16:1;2O/16:0 | 9.16 | 5.70 | 6.37 | 6.93 | 3.64 | 3.75 | 4.02 | 3.81 | 4.35 | 4.12 | 3.88 | 4.12 | 3.00 | 2.71 | 3.01 | 2.91 |
| SM 32:2;2O\|SM 18:1;2O/14:1 | 0.25 | 0.16 | 0.16 | 0.19 | 0.12 | 0.12 | 0.11 | 0.11 | 0.11 | 0.12 | 0.12 | 0.12 | 0.11 | 0.11 | 0.12 | 0.11 |
| SM 33:1;2O\|SM 17:1;2O/16:0 | 2.10 | 1.57 | 1.93 | 1.84 | 1.62 | 1.48 | 1.83 | 1.64 | 1.32 | 1.33 | 1.30 | 1.32 | 1.59 | 1.50 | 1.43 | 1.51 |
| SM 34:1;2O\|SM 18:0;2O/16:1 | 2.51 | 2.64 | 2.89 | 2.68 | 4.42 | 4.58 | 3.96 | 4.32 | 3.54 | 3.28 | 3.64 | 3.49 | 2.66 | 2.26 | 2.42 | 2.45 |
| SM 34:1;2O\|SM 18:1;2O/16:0 | 25.91 | 19.80 | 25.35 | 23.43 | 19.93 | 18.34 | 23.67 | 20.62 | 17.18 | 16.79 | 18.21 | 17.41 | 18.20 | 14.93 | 12.70 | 15.28 |
| SM 34:2;2O\|SM 18:1;2O/16:1 | 1.10 | 0.76 | 0.72 | 0.85 | 1.01 | 0.90 | 1.03 | 0.98 | 0.68 | 0.69 | 0.65 | 0.67 | 0.75 | 0.74 | 0.63 | 0.70 |
| SM 34:4;2O\|SM 18:1;2O/16:3 | 0.36 | 0.39 | 0.37 | 0.37 | 0.41 | 0.40 | 0.23 | 0.35 | 0.37 | 0.38 | 0.21 | 0.32 | 0.28 | 0.26 | 0.24 | 0.26 |
| SM 35:1;2O\|SM 18:1;2O/17:0 | 1.92 | 1.55 | 1.82 | 1.75 | 1.59 | 1.53 | 1.31 | 1.48 | 1.86 | 2.01 | 1.54 | 1.80 | 1.93 | 1.70 | 1.72 | 1.78 |
| SM 35:2;2O\|SM 18:1;2O/17:1 | 0.46 | 0.34 | 0.34 | 0.37 | 0.47 | 0.44 | 0.37 | 0.43 | 0.29 | 0.38 | 0.42 | 0.36 | 0.44 | 0.40 | 0.36 | 0.40 |
| SM 36:1;2O\|SM 18:1;2O/18:0 | 2.71 | 2.50 | 3.16 | 2.78 | 1.25 | 1.06 | 0.94 | 1.08 | 5.43 | 4.65 | 4.38 | 4.84 | 1.53 | 1.20 | 1.38 | 1.37 |
| SM 36:2;2O\|SM 16:2;2O/20:0 | 0.17 | 0.18 | 0.21 | 0.19 | 0.38 | 0.40 | 0.40 | 0.40 | 0.48 | 0.50 | 0.47 | 0.48 | 0.24 | 0.25 | 0.20 | 0.23 |
| SM 36:3;2O\|SM 18:1;2O/18:2 | 0.20 | 0.14 | 0.16 | 0.16 | 0.31 | 0.26 | 0.28 | 0.28 | 0.20 | 0.22 | 0.18 | 0.20 | 0.31 | 0.29 | 0.31 | 0.30 |
| SM 37:1;2O\|SM 18:0;2O/19:1 | 1.50 | 1.15 | 1.37 | 1.33 | 1.35 | 1.30 | 1.12 | 1.26 | 1.67 | 1.82 | 1.87 | 1.78 | 1.50 | 1.44 | 1.65 | 1.53 |
| SM 37:2;2O\|SM 20:0;2O/17:2 | 0.54 | 0.44 | 0.60 | 0.52 | 0.62 | 0.58 | 0.62 | 0.61 | 0.45 | 0.54 | 0.54 | 0.51 | 0.61 | 0.57 | 0.60 | 0.59 |
| SM 38:1;2O\|SM 16:1;2O/22:0 | 2.03 | 1.78 | 2.46 | 2.08 | 1.67 | 1.53 | 1.33 | 1.51 | 5.09 | 4.18 | 4.49 | 4.61 | 3.01 | 2.43 | 2.50 | 2.64 |
| SM 38:1;2O\|SM 20:0;2O/18:1 | 0.29 | 0.38 | 0.45 | 0.37 | 0.62 | 0.63 | 0.80 | 0.68 | 1.37 | 1.18 | 1.22 | 1.26 | 0.53 | 0.43 | 0.38 | 0.45 |
| SM 39:1;2O\|SM 16:1;2O/23:0 | 2.63 | 1.80 | 1.99 | 2.10 | 1.46 | 1.37 | 1.39 | 1.41 | 3.53 | 3.55 | 2.89 | 3.32 | 2.72 | 2.03 | 1.85 | 2.20 |
| SM 39:1;2O\|SM 25:0;2O/14:1 | 0.46 | 0.55 | 0.57 | 0.53 | 0.71 | 0.71 | 0.81 | 0.74 | 1.05 | 1.04 | 1.17 | 1.09 | 0.67 | 0.57 | 0.64 | 0.63 |
| SM 40:1;2O\|SM 12:0;2O/28:1 | 3.17 | 4.38 | 4.75 | 4.10 | 7.09 | 7.17 | 8.15 | 7.47 | 6.64 | 6.25 | 4.86 | 5.93 | 5.11 | 4.08 | 4.97 | 4.72 |
| SM 40:1;2O\|SM 18:1;2O/22:0 | 14.72 | 13.21 | 15.49 | 14.41 | 14.97 | 13.35 | 14.79 | 14.34 | 20.26 | 19.20 | 20.27 | 19.94 | 20.81 | 14.37 | 12.65 | 15.94 |
| SM 40:2;2O\|SM 18:0;2O/22:2 | 11.71 | 8.56 | 8.89 | 9.59 | 2.47 | 2.27 | 3.04 | 2.59 | 2.04 | 2.29 | 2.05 | 2.12 | 2.31 | 2.55 | 2.94 | 2.60 |
| SM 40:3;2O\|SM 16:1;2O/24:2 | 2.40 | 1.62 | 2.11 | 2.01 | 0.61 | 0.56 | 0.51 | 0.56 | 0.37 | 0.39 | 0.44 | 0.40 | 0.43 | 0.45 | 0.57 | 0.48 |
| SM 40:4;2O\|SM 18:1;2O/22:3 | 0.25 | 0.17 | 0.25 | 0.22 | 0.16 | 0.15 | 0.20 | 0.17 | 0.06 | 0.07 | 0.08 | 0.07 | 0.20 | 0.22 | 0.26 | 0.23 |
| SM 41:1;2O\|SM 18:1;2O/23:0 | 18.91 | 18.37 | 19.56 | 18.93 | 14.76 | 12.63 | 16.99 | 14.76 | 29.51 | 24.61 | 25.53 | 26.70 | 29.76 | 18.89 | 20.79 | 23.13 |
| SM 41:2;2O\|SM 16:2;2O/25:0 | 0.36 | 0.34 | 0.38 | 0.36 | 0.35 | 0.37 | 0.33 | 0.35 | 0.33 | 0.38 | 0.33 | 0.34 | 0.32 | 0.36 | 0.42 | 0.37 |
| SM 42:1;2O\|SM 18:1;2O/24:0 | 14.95 | 15.20 | 20.18 | 16.79 | 14.42 | 11.87 | 10.16 | 12.11 | 27.38 | 20.40 | 20.04 | 22.82 | 26.42 | 15.33 | 19.83 | 20.51 |
| SM 42:2;2O\|SM 18:0;2O/24:2 | 11.29 | 6.97 | 7.12 | 8.28 | 4.14 | 3.92 | 3.63 | 3.90 | 7.13 | 8.73 | 7.30 | 7.67 | 8.04 | 8.40 | 10.59 | 9.01 |
| SM 43:1;2O\|SM 18:1;2O/25:0 | 1.69 | 1.69 | 2.01 | 1.80 | 1.21 | 1.09 | 1.27 | 1.19 | 4.13 | 2.86 | 2.73 | 3.28 | 4.14 | 2.57 | 3.29 | 3.33 |
| TG 28:0\|TG 8:0_8:0_12:0 | 1798.33 | 1846.84 | 1018.98 | 1554.68 | 1664.90 | 1722.43 | 1985.01 | 1790.31 | 1838.50 | 1815.43 | 1487.88 | 1712.63 | 1787.92 | 1950.02 | 1152.17 | 1629.90 |
| TG 29:0\|TG 8:0_10:0_11:0 | 213.61 | 217.82 | 157.78 | 196.40 | 213.61 | 214.77 | 135.20 | 187.85 | 236.34 | 227.94 | 189.57 | 217.79 | 220.47 | 237.60 | 168.68 | 208.90 |
| TG 30:0\|TG 8:0_10:0_12:0 | 2206.86 | 2275.28 | 2355.64 | 2279.20 | 1838.45 | 1901.82 | 1199.19 | 1645.96 | 2191.22 | 2108.60 | 2151.95 | 2148.75 | 2042.64 | 2196.65 | 2136.91 | 2125.26 |
| TG 31:0\|TG 8:0_11:0_12:0 | 289.86 | 300.27 | 146.82 | 245.64 | 236.93 | 234.85 | 141.74 | 204.52 | 284.09 | 276.59 | 241.01 | 267.02 | 257.66 | 269.02 | 251.72 | 259.46 |
| TG 32:0\|TG 8:0_10:0_14:0 | 2260.78 | 2361.95 | 1387.61 | 2003.36 | 1744.22 | 1732.07 | 1405.75 | 1627.45 | 2093.44 | 2005.68 | 1564.24 | 1886.47 | 1880.16 | 2107.94 | 1888.42 | 1958.63 |
| TG 32:1\|TG 8:0_8:0_16:1 | 1258.71 | 1301.45 | 1197.30 | 1252.45 | 1257.10 | 1281.35 | 1087.28 | 1208.38 | 1116.95 | 1110.93 | 776.76 | 1000.86 | 1149.00 | 1166.65 | 585.81 | 967.14 |
| TG 33:0\|TG 8:0_10:0_15:0 | 343.67 | 353.11 | 400.35 | 365.70 | 259.10 | 266.20 | 268.54 | 264.55 | 321.68 | 305.17 | 183.35 | 269.92 | 261.24 | 264.49 | 239.72 | 255.15 |
| TG 33:1\|TG 8:0_9:0_16:1 | 111.10 | 112.90 | 55.80 | 93.26 | 122.53 | 121.37 | 75.35 | 106.43 | 107.45 | 105.28 | 104.32 | 105.59 | 108.15 | 113.19 | 56.38 | 92.57 |
| TG 33:4\|TG 8:0_9:0_16:4 | 0.36 | 0.36 | 0.29 | 0.34 | 0.91 | 0.91 | 0.90 | 0.90 | 1.04 | 1.07 | 1.19 | 1.10 | 1.64 | 1.66 | 1.53 | 1.61 |
| TG 34:0\|TG 8:0_12:0_14:0 | 2713.19 | 2781.23 | 2543.04 | 2679.09 | 1716.36 | 1728.52 | 1589.05 | 1677.88 | 2120.75 | 2025.07 | 1168.50 | 1770.48 | 1691.62 | 1838.61 | 1392.38 | 1640.74 |
| TG 34:1\|TG 8:0_10:0_16:1 | 1344.55 | 1336.42 | 739.20 | 1140.06 | 1312.14 | 1313.44 | 883.80 | 1169.78 | 1221.30 | 1153.84 | 1362.97 | 1244.88 | 1168.58 | 1230.76 | 1013.94 | 1137.70 |
| TG 34:2\|TG 8:0_8:0_18:2 | 791.51 | 817.01 | 443.64 | 684.03 | 931.56 | 927.15 | 663.12 | 840.64 | 771.88 | 739.99 | 517.86 | 676.15 | 1028.33 | 1086.32 | 1182.87 | 1099.12 |
| TG 34:3\|TG 8:0_8:0_18:3 | 89.20 | 90.16 | 73.11 | 84.16 | 178.58 | 183.39 | 100.85 | 154.24 | 103.76 | 101.12 | 103.17 | 102.59 | 171.60 | 175.25 | 119.84 | 155.56 |
| TG 34:4\|TG 8:0_8:0_18:4 | 10.21 | 10.46 | 12.18 | 10.95 | 18.18 | 18.56 | 19.66 | 18.80 | 27.07 | 26.65 | 21.29 | 24.99 | 41.65 | 43.42 | 50.01 | 45.03 |
| TG 35:0\|TG 9:0_10:0_16:0 | 351.28 | 354.92 | 295.31 | 333.83 | 241.02 | 250.56 | 154.29 | 215.21 | 324.24 | 319.98 | 241.18 | 294.92 | 245.42 | 268.79 | 302.27 | 272.14 |
| TG 35:1\|TG 8:0_10:0_17:1 | 153.32 | 158.61 | 147.37 | 153.09 | 151.23 | 155.34 | 182.11 | 162.86 | 142.94 | 140.84 | 100.18 | 127.90 | 128.63 | 134.80 | 156.29 | 139.90 |
| TG 35:2\|TG 8:0_9:0_18:2 | 16.24 | 16.91 | 16.81 | 16.65 | 42.81 | 43.54 | 23.71 | 36.68 | 22.33 | 21.96 | 15.92 | 20.05 | 34.91 | 37.01 | 35.12 | 35.67 |
| TG 35:3\|TG 8:0_9:0_18:3 | 1.76 | 1.80 | 1.86 | 1.80 | 4.97 | 5.14 | 5.59 | 5.23 | 2.39 | 2.30 | 2.25 | 2.31 | 3.50 | 4.15 | 2.23 | 3.29 |
| TG 35:4\|TG 8:0_9:0_18:4 | 0.00 | 0.00 | 0.00 | 0.00 | 1.06 | 1.17 | 1.23 | 1.16 | 1.81 | 1.76 | 2.19 | 1.92 | 2.77 | 2.93 | 3.25 | 2.98 |
| TG 36:0\|TG 10:0_12:0_14:0 | 2548.99 | 2548.20 | 2753.48 | 2616.89 | 1498.45 | 1539.36 | 1765.75 | 1600.85 | 2001.88 | 1907.99 | 1192.81 | 1699.91 | 1631.82 | 1679.14 | 1761.28 | 1690.70 |
| TG 36:1\|TG 8:0_10:0_18:1 | 1645.05 | 1679.38 | 1777.56 | 1700.64 | 1480.80 | 1473.46 | 1296.10 | 1416.85 | 1397.01 | 1349.12 | 877.81 | 1207.24 | 1268.44 | 1385.79 | 1103.48 | 1252.47 |
| TG 36:2\|TG 8:0_10:0_18:2 | 1134.92 | 1158.56 | 973.30 | 1088.91 | 1173.25 | 1207.98 | 736.66 | 1039.01 | 952.18 | 921.61 | 648.10 | 840.08 | 1207.12 | 1251.22 | 1421.22 | 1293.15 |
| TG 36:3\|TG 8:0_10:0_18:3 | 169.93 | 171.16 | 193.38 | 178.15 | 253.15 | 254.20 | 233.19 | 246.84 | 164.56 | 160.51 | 147.20 | 157.30 | 249.53 | 259.80 | 292.66 | 267.32 |
| TG 36:4\|TG 8:0_10:0_18:4 | 14.32 | 14.37 | 12.00 | 13.56 | 22.34 | 21.94 | 11.51 | 18.60 | 31.36 | 30.12 | 35.71 | 32.37 | 45.14 | 47.61 | 30.54 | 41.10 |
| TG 37:0\|TG 10:0_12:0_15:0 | 308.89 | 306.68 | 269.01 | 294.86 | 192.55 | 196.12 | 198.47 | 195.68 | 287.82 | 278.13 | 354.11 | 306.38 | 220.58 | 230.42 | 117.07 | 189.35 |
| TG 37:1\|TG 9:0_10:0_18:1 | 247.68 | 244.05 | 182.44 | 224.73 | 208.55 | 216.72 | 238.00 | 221.02 | 213.34 | 210.63 | 114.97 | 179.55 | 173.23 | 183.58 | 166.34 | 174.37 |
| TG 37:2\|TG 9:0_10:0_18:2 | 52.52 | 54.58 | 64.51 | 57.20 | 102.51 | 105.44 | 75.37 | 94.42 | 56.85 | 55.16 | 64.61 | 58.82 | 80.36 | 85.88 | 77.05 | 81.09 |
| TG 37:3\|TG 9:0_10:0_18:3 | 6.58 | 6.66 | 3.96 | 5.73 | 14.28 | 14.78 | 8.57 | 12.54 | 7.65 | 7.38 | 4.68 | 6.56 | 11.30 | 11.90 | 7.85 | 10.35 |
| TG 37:4\|TG 8:0_9:0_20:4 | 1.33 | 1.36 | 1.17 | 1.29 | 2.79 | 2.71 | 1.75 | 2.42 | 3.15 | 3.19 | 2.75 | 3.03 | 4.96 | 5.05 | 4.08 | 4.70 |
| TG 38:0\|TG 10:0_12:0_16:0 | 2100.44 | 2128.60 | 2443.55 | 2224.17 | 1169.98 | 1198.02 | 1285.59 | 1217.63 | 1575.23 | 1552.83 | 1825.80 | 1649.68 | 1259.51 | 1323.98 | 1310.79 | 1298.04 |
| TG 38:1\|TG 10:0_10:0_18:1 | 2133.42 | 2159.84 | 2418.34 | 2237.18 | 1904.10 | 1944.99 | 1017.81 | 1621.96 | 1924.56 | 1850.30 | 1199.38 | 1657.08 | 1747.39 | 1818.80 | 1854.47 | 1806.83 |
| TG 38:2\|TG 10:0_10:0_18:2 | 1610.03 | 1610.04 | 1581.16 | 1600.41 | 1641.98 | 1682.99 | 1659.35 | 1661.11 | 1346.18 | 1318.80 | 1641.99 | 1434.22 | 1622.30 | 1736.22 | 964.71 | 1440.97 |
| TG 38:3\|TG 10:0_10:0_18:3 | 358.54 | 368.28 | 347.85 | 358.21 | 520.84 | 532.12 | 367.32 | 473.34 | 299.96 | 291.87 | 318.45 | 303.15 | 443.82 | 465.76 | 310.16 | 406.56 |
| TG 38:5\|TG 8:0_10:0_20:5 | 8.00 | 8.11 | 9.25 | 8.45 | 15.17 | 15.88 | 15.23 | 15.42 | 48.32 | 47.63 | 48.46 | 48.09 | 62.32 | 65.09 | 72.09 | 66.50 |
| TG 38:6\|TG 8:0_8:0_22:6 | 1.02 | 1.03 | 0.95 | 1.00 | 4.01 | 4.21 | 4.62 | 4.28 | 12.95 | 13.39 | 12.69 | 13.00 | 20.96 | 21.74 | 23.36 | 22.02 |
| TG 39:0\|TG 8:0_15:0_16:0 | 261.87 | 261.35 | 173.44 | 232.22 | 145.22 | 150.10 | 150.34 | 148.51 | 220.64 | 212.61 | 129.14 | 187.36 | 155.77 | 167.27 | 149.17 | 157.39 |
| TG 39:1\|TG 10:0_11:0_18:1 | 290.23 | 280.12 | 224.92 | 265.10 | 243.12 | 249.00 | 287.99 | 259.99 | 250.08 | 249.12 | 166.59 | 221.78 | 214.49 | 223.86 | 189.81 | 209.38 |
| TG 39:2\|TG 10:0_11:0_18:2 | 76.30 | 76.52 | 68.17 | 73.67 | 111.90 | 121.81 | 97.48 | 110.32 | 74.67 | 73.01 | 47.69 | 65.09 | 98.60 | 102.56 | 77.86 | 93.01 |
| TG 39:3\|TG 10:0_11:0_18:3 | 11.63 | 12.11 | 8.00 | 10.58 | 23.30 | 23.79 | 19.34 | 22.14 | 12.13 | 12.08 | 11.80 | 11.99 | 16.54 | 18.30 | 15.29 | 16.71 |
| TG 40:0\|TG 10:0_14:0_16:0 | 1748.77 | 1752.64 | 1646.83 | 1716.08 | 870.60 | 890.14 | 490.12 | 750.13 | 1160.20 | 1136.85 | 821.43 | 1038.78 | 880.22 | 911.95 | 675.20 | 822.43 |
| TG 40:1\|TG 10:0_12:0_18:1 | 1760.71 | 1852.70 | 962.57 | 1525.24 | 1571.30 | 1589.74 | 1472.51 | 1544.36 | 1573.75 | 1556.02 | 1495.12 | 1540.31 | 1417.46 | 1521.08 | 1622.34 | 1520.20 |
| TG 40:2\|TG 10:0_12:0_18:2 | 1525.54 | 1500.03 | 1383.76 | 1469.80 | 1632.79 | 1691.80 | 1571.90 | 1631.68 | 1304.26 | 1269.78 | 1324.68 | 1298.42 | 1443.07 | 1534.48 | 842.52 | 1273.27 |
| TG 41:0\|TG 10:0_15:0_16:0 | 228.70 | 231.52 | 269.32 | 243.18 | 114.78 | 122.55 | 121.45 | 119.53 | 183.06 | 176.38 | 229.90 | 196.25 | 129.84 | 136.52 | 119.77 | 128.70 |
| TG 41:1\|TG 8:0_15:0_18:1 | 235.59 | 236.85 | 146.98 | 206.47 | 188.61 | 196.94 | 231.04 | 205.46 | 206.09 | 198.82 | 116.01 | 173.54 | 162.54 | 173.82 | 179.44 | 171.92 |
| TG 41:2\|TG 8:0_15:0_18:2 | 84.38 | 80.09 | 61.73 | 75.40 | 126.58 | 128.90 | 125.02 | 126.81 | 84.00 | 86.86 | 69.03 | 79.90 | 113.68 | 118.97 | 123.26 | 118.63 |
| TG 41:3\|TG 8:0_15:0_18:3 | 10.55 | 10.88 | 8.31 | 9.91 | 21.48 | 21.25 | 13.03 | 18.59 | 11.84 | 11.58 | 7.47 | 10.29 | 16.87 | 17.48 | 21.12 | 18.49 |
| TG 42:0\|TG 10:0_14:0_18:0 | 1432.48 | 1445.64 | 1721.06 | 1533.05 | 674.00 | 741.16 | 818.59 | 744.03 | 959.98 | 889.64 | 974.94 | 940.71 | 752.97 | 754.35 | 939.08 | 815.47 |
| TG 42:1\|TG 8:0_16:0_18:1 | 1467.12 | 1507.63 | 1348.72 | 1441.12 | 1167.73 | 1209.68 | 1427.17 | 1267.85 | 1233.23 | 1187.68 | 937.10 | 1118.54 | 1059.27 | 1139.80 | 1316.40 | 1171.75 |
| TG 42:2\|TG 10:0_14:0_18:2 | 1289.64 | 1317.38 | 1496.21 | 1367.72 | 1325.40 | 1365.63 | 1389.49 | 1359.84 | 1091.80 | 1056.37 | 651.66 | 932.73 | 1169.49 | 1235.03 | 1271.27 | 1225.20 |
| TG 43:0\|TG 10:0_16:0_17:0 | 194.35 | 188.16 | 156.87 | 179.80 | 98.43 | 103.35 | 50.61 | 84.09 | 151.40 | 149.70 | 142.91 | 147.88 | 112.08 | 110.86 | 64.68 | 95.87 |
| TG 43:1\|TG 10:0_15:0_18:1 | 247.99 | 249.32 | 163.20 | 220.17 | 196.29 | 198.11 | 200.54 | 198.30 | 220.65 | 217.28 | 122.55 | 186.72 | 182.05 | 187.69 | 147.40 | 172.38 |
| TG 43:2\|TG 10:0_15:0_18:2 | 101.56 | 102.04 | 90.34 | 97.98 | 136.56 | 143.80 | 86.80 | 122.33 | 96.00 | 92.41 | 105.27 | 97.80 | 112.65 | 119.01 | 126.94 | 119.53 |
| TG 43:3\|TG 8:0_17:1_18:2 | 13.00 | 13.50 | 9.47 | 11.99 | 29.61 | 31.04 | 21.62 | 27.41 | 16.18 | 16.42 | 19.69 | 17.41 | 21.72 | 22.72 | 15.90 | 20.11 |
| TG 43:6\|TG 9:0_12:0_22:6 | 1.51 | 1.57 | 0.84 | 1.30 | 3.77 | 3.92 | 4.26 | 3.98 | 14.97 | 15.62 | 13.58 | 14.71 | 22.82 | 24.88 | 16.37 | 21.36 |
| TG 44:0\|TG 12:0_14:0_18:0 | 1032.01 | 989.60 | 588.86 | 870.19 | 440.26 | 421.35 | 340.90 | 400.99 | 545.41 | 516.23 | 582.56 | 547.57 | 340.01 | 342.89 | 259.59 | 314.16 |
| TG 44:1\|TG 10:0_16:0_18:1 | 1632.14 | 1588.44 | 2018.22 | 1746.30 | 1254.59 | 1298.62 | 781.04 | 1111.06 | 1346.85 | 1340.79 | 833.43 | 1172.96 | 1157.71 | 1238.03 | 667.17 | 1020.90 |
| TG 44:2\|TG 10:0_16:0_18:2 | 1199.79 | 1223.43 | 956.87 | 1126.67 | 1151.38 | 1178.15 | 1224.78 | 1184.55 | 996.93 | 972.44 | 589.25 | 852.37 | 1095.75 | 1157.69 | 1291.80 | 1181.69 |
| TG 44:3\|TG 8:0_18:1_18:2 | 507.78 | 509.66 | 537.20 | 518.21 | 693.02 | 733.25 | 737.17 | 720.82 | 466.11 | 441.37 | 480.66 | 462.30 | 589.66 | 603.50 | 542.03 | 578.39 |
| TG 44:4\|TG 8:0_18:2_18:2 | 111.14 | 112.73 | 86.57 | 103.48 | 266.83 | 278.84 | 187.78 | 244.39 | 173.81 | 181.05 | 125.17 | 159.89 | 301.95 | 317.77 | 337.39 | 319.02 |
| TG 45:0\|TG 14:0_15:0_16:0 | 104.48 | 110.46 | 124.09 | 113.01 | 53.33 | 54.04 | 39.24 | 48.87 | 70.25 | 70.97 | 45.33 | 62.14 | 42.88 | 44.61 | 27.96 | 38.48 |
| TG 45:1\|TG 10:0_17:0_18:1 | 232.28 | 230.52 | 258.53 | 240.44 | 192.35 | 198.64 | 135.37 | 175.40 | 218.63 | 214.86 | 184.15 | 205.72 | 179.23 | 182.12 | 199.69 | 187.01 |
| TG 45:2\|TG 10:0_17:1_18:1 | 105.12 | 104.72 | 102.35 | 104.06 | 121.01 | 126.73 | 152.76 | 133.45 | 96.46 | 92.91 | 67.79 | 85.66 | 113.41 | 116.99 | 127.94 | 119.44 |
| TG 45:3\|TG 10:0_17:1_18:2 | 16.10 | 16.07 | 13.62 | 15.26 | 33.42 | 31.85 | 39.63 | 34.98 | 18.72 | 18.13 | 10.69 | 15.84 | 23.79 | 24.60 | 27.10 | 25.16 |
| TG 45:4\|TG 10:0_15:0_20:4 | 4.21 | 4.09 | 3.91 | 4.07 | 10.06 | 10.70 | 10.07 | 10.27 | 10.03 | 9.27 | 12.20 | 10.49 | 13.14 | 13.78 | 16.31 | 14.41 |
| TG 45:5\|TG 8:0_15:0_22:5 | 0.00 | 0.00 | 0.00 | 0.00 | 5.64 | 5.66 | 2.91 | 4.74 | 12.19 | 11.96 | 8.26 | 10.80 | 17.58 | 18.89 | 21.25 | 19.24 |
| TG 45:6\|TG 8:0_15:0_22:6 | 0.00 | 0.00 | 0.00 | 0.00 | 2.57 | 2.14 | 1.48 | 2.07 | 10.70 | 10.90 | 7.75 | 9.77 | 15.84 | 16.03 | 8.59 | 13.49 |
| TG 46:0\|TG 12:0_16:0_18:0 | 530.69 | 534.62 | 292.61 | 452.64 | 199.39 | 209.86 | 229.81 | 212.94 | 244.23 | 228.47 | 219.74 | 230.63 | 144.01 | 144.19 | 81.33 | 123.18 |
| TG 46:1\|TG 12:0_16:0_18:1 | 1039.15 | 1109.75 | 1136.43 | 1095.05 | 760.16 | 770.05 | 420.53 | 650.17 | 785.33 | 756.57 | 814.62 | 784.81 | 568.21 | 598.53 | 625.87 | 597.51 |
| TG 46:2\|TG 10:0_18:1_18:1 | 1115.56 | 1118.48 | 592.58 | 942.20 | 1163.19 | 1205.80 | 1242.93 | 1203.62 | 952.91 | 941.47 | 852.29 | 914.81 | 941.65 | 961.93 | 1154.72 | 1019.42 |
| TG 46:3\|TG 10:0_18:1_18:2 | 657.35 | 660.22 | 560.14 | 625.90 | 889.74 | 908.21 | 640.58 | 812.69 | 602.92 | 585.51 | 603.61 | 596.82 | 751.11 | 772.50 | 719.17 | 747.57 |
| TG 46:4\|TG 10:0_18:2_18:2 | 207.13 | 207.37 | 247.13 | 220.54 | 406.33 | 405.40 | 246.55 | 352.76 | 248.87 | 235.69 | 300.74 | 261.51 | 390.22 | 411.15 | 386.32 | 395.88 |
| TG 46:6\|TG 8:0_16:0_22:6 | 25.57 | 25.86 | 29.15 | 26.86 | 59.66 | 60.04 | 46.93 | 55.54 | 258.10 | 252.37 | 138.32 | 216.15 | 348.13 | 351.29 | 263.53 | 320.98 |
| TG 47:0\|TG 14:0_16:0_17:0 | 57.68 | 58.72 | 29.11 | 48.50 | 25.91 | 27.59 | 20.78 | 24.75 | 38.14 | 36.34 | 30.99 | 35.13 | 24.03 | 24.38 | 21.50 | 23.30 |
| TG 47:1\|TG 14:0_15:0_18:1 | 143.57 | 149.34 | 152.44 | 148.45 | 87.76 | 89.64 | 50.35 | 75.90 | 109.64 | 105.74 | 130.16 | 115.06 | 65.38 | 67.95 | 46.84 | 60.05 |
| TG 47:2\|TG 12:0_17:1_18:1 | 104.14 | 102.39 | 68.91 | 91.82 | 106.10 | 111.75 | 70.13 | 95.95 | 92.28 | 86.53 | 100.39 | 92.98 | 89.84 | 91.80 | 95.79 | 92.47 |
| TG 47:3\|TG 11:0_18:1_18:2 | 14.77 | 15.31 | 15.16 | 15.08 | 24.81 | 25.52 | 22.07 | 24.13 | 15.44 | 15.60 | 12.70 | 14.57 | 19.61 | 19.64 | 15.85 | 18.37 |
| TG 47:6\|TG 10:0_15:0_22:6 | 0.00 | 0.00 | 0.00 | 0.00 | 2.79 | 3.44 | 3.27 | 3.16 | 13.08 | 13.92 | 15.61 | 14.19 | 19.78 | 20.17 | 21.86 | 20.60 |
| TG 48:0\|TG 14:0_16:0_18:0 | 299.92 | 288.83 | 344.85 | 311.21 | 107.82 | 122.29 | 113.24 | 114.33 | 127.99 | 131.19 | 117.52 | 125.46 | 99.35 | 94.92 | 69.91 | 88.06 |
| TG 48:1\|TG 14:0_16:0_18:1 | 911.26 | 954.55 | 1131.99 | 999.23 | 540.22 | 570.51 | 328.49 | 479.49 | 665.35 | 653.77 | 612.31 | 643.28 | 476.53 | 469.14 | 265.83 | 403.84 |
| TG 48:2\|TG 14:0_16:0_18:2 | 886.83 | 911.41 | 1003.51 | 933.89 | 738.25 | 766.39 | 657.78 | 720.57 | 675.16 | 660.73 | 411.83 | 582.22 | 579.63 | 607.76 | 341.13 | 509.48 |
| TG 48:3\|TG 12:0_18:1_18:2 | 390.77 | 401.63 | 426.52 | 406.30 | 460.11 | 479.55 | 573.62 | 504.27 | 317.62 | 312.80 | 368.50 | 332.65 | 361.02 | 369.92 | 219.49 | 316.80 |
| TG 48:4\|TG 12:0_18:2_18:2 | 153.31 | 155.37 | 142.08 | 150.25 | 211.74 | 204.97 | 247.55 | 221.47 | 187.34 | 192.09 | 218.28 | 199.04 | 241.65 | 256.18 | 273.49 | 257.09 |
| TG 48:5\|TG 10:0_16:0_22:5 | 32.16 | 29.94 | 18.51 | 26.87 | 78.28 | 78.20 | 88.00 | 81.49 | 152.67 | 147.55 | 165.52 | 155.10 | 199.74 | 194.86 | 123.79 | 172.80 |
| TG 48:6\|TG 10:0_16:0_22:6 | 38.90 | 39.91 | 44.87 | 41.23 | 69.90 | 74.45 | 47.36 | 63.86 | 331.71 | 329.47 | 379.43 | 346.53 | 416.52 | 414.29 | 470.24 | 433.69 |
| TG 49:0\|TG 15:0_16:0_18:0 | 51.90 | 53.33 | 37.36 | 47.53 | 22.24 | 23.38 | 18.54 | 21.38 | 34.47 | 31.79 | 37.13 | 34.43 | 24.57 | 25.76 | 28.01 | 26.11 |
| TG 49:1\|TG 15:0_16:0_18:1 | 151.41 | 153.02 | 86.17 | 130.20 | 90.86 | 95.53 | 115.04 | 100.44 | 137.76 | 136.25 | 100.88 | 124.87 | 94.80 | 98.23 | 87.69 | 93.57 |
| TG 49:2\|TG 15:0_16:0_18:2 | 129.06 | 135.05 | 86.95 | 117.01 | 112.05 | 112.40 | 142.33 | 122.26 | 113.41 | 110.15 | 81.66 | 101.67 | 96.13 | 102.63 | 106.48 | 101.74 |
| TG 49:3\|TG 13:0_18:1_18:2 | 27.19 | 27.15 | 17.30 | 23.88 | 40.07 | 41.04 | 37.19 | 39.43 | 28.05 | 26.92 | 21.89 | 25.60 | 32.24 | 32.59 | 26.23 | 30.35 |
| TG 49:5\|TG 10:0_17:0_22:5 | 0.00 | 0.00 | 0.00 | 0.00 | 4.02 | 3.98 | 2.25 | 3.42 | 10.82 | 11.07 | 11.40 | 11.09 | 16.52 | 16.86 | 17.82 | 17.06 |
| TG 49:6\|TG 10:0_17:0_22:6 | 0.00 | 0.00 | 0.00 | 0.00 | 3.21 | 3.36 | 3.17 | 3.25 | 12.09 | 12.42 | 8.48 | 10.99 | 13.60 | 15.57 | 16.41 | 15.19 |
| TG 50:0\|TG 16:0_16:0_18:0 | 284.95 | 286.94 | 341.79 | 304.56 | 111.23 | 115.75 | 130.86 | 119.24 | 177.03 | 174.60 | 129.47 | 160.25 | 114.33 | 114.94 | 137.79 | 122.35 |
| TG 50:1\|TG 16:0_16:0_18:1 | 841.66 | 865.79 | 791.40 | 832.93 | 546.87 | 570.76 | 449.50 | 522.18 | 777.51 | 760.87 | 949.51 | 828.47 | 644.55 | 658.75 | 564.81 | 622.69 |
| TG 50:2\|TG 16:0_16:1_18:1 | 800.25 | 840.66 | 714.93 | 785.24 | 661.47 | 653.52 | 444.11 | 586.43 | 702.16 | 685.63 | 594.18 | 660.14 | 617.29 | 639.94 | 770.29 | 675.82 |
| TG 50:3\|TG 14:0_18:1_18:2 | 583.43 | 593.07 | 431.61 | 536.03 | 567.66 | 581.93 | 307.23 | 485.49 | 423.35 | 418.64 | 438.90 | 426.58 | 414.79 | 448.41 | 424.35 | 429.15 |
| TG 50:4\|TG 14:0_18:2_18:2 | 213.32 | 214.92 | 224.10 | 217.44 | 285.10 | 289.93 | 224.15 | 266.35 | 192.75 | 188.40 | 172.33 | 184.35 | 263.10 | 274.95 | 201.21 | 246.41 |
| TG 50:6\|TG 12:0_16:0_22:6 | 17.46 | 17.68 | 11.84 | 15.66 | 34.95 | 35.83 | 28.97 | 33.24 | 111.47 | 110.63 | 93.41 | 105.09 | 156.19 | 156.93 | 172.86 | 161.99 |
| TG 51:0\|TG 16:0_17:0_18:0 | 64.23 | 62.71 | 58.25 | 61.73 | 29.85 | 33.17 | 16.31 | 26.42 | 54.80 | 53.33 | 60.58 | 56.19 | 34.60 | 34.71 | 38.24 | 35.85 |
| TG 51:1\|TG 16:0_17:0_18:1 | 168.55 | 172.47 | 85.35 | 142.12 | 112.24 | 122.02 | 73.64 | 102.55 | 176.04 | 170.03 | 94.94 | 146.93 | 143.42 | 140.64 | 95.35 | 126.47 |
| TG 51:2\|TG 15:0_18:1_18:1 | 164.11 | 167.28 | 172.69 | 168.02 | 140.92 | 146.46 | 137.64 | 141.63 | 160.43 | 167.50 | 164.14 | 163.87 | 143.69 | 142.05 | 111.58 | 132.44 |
| TG 51:3\|TG 15:0_18:1_18:2 | 72.88 | 78.33 | 76.68 | 75.96 | 106.78 | 108.88 | 104.07 | 106.56 | 77.11 | 75.80 | 75.22 | 75.98 | 79.06 | 82.30 | 44.06 | 68.47 |
| TG 51:4\|TG 15:0_18:2_18:2 | 16.50 | 16.89 | 20.68 | 18.02 | 39.04 | 38.68 | 32.30 | 36.67 | 24.99 | 24.98 | 26.17 | 25.36 | 32.47 | 35.66 | 29.25 | 32.46 |
| TG 52:0\|TG 16:0_18:0_18:0 | 189.80 | 184.48 | 234.67 | 202.99 | 64.12 | 66.21 | 81.36 | 70.54 | 109.42 | 104.18 | 89.12 | 100.83 | 54.34 | 55.77 | 50.76 | 53.62 |
| TG 52:1\|TG 16:0_18:0_18:1 | 722.22 | 756.98 | 495.71 | 658.27 | 403.56 | 442.53 | 413.29 | 419.47 | 541.66 | 513.90 | 603.26 | 552.43 | 356.07 | 359.66 | 199.44 | 305.05 |
| TG 52:2\|TG 16:0_18:1_18:1 | 1052.33 | 1069.04 | 871.70 | 997.67 | 1018.69 | 1010.42 | 829.84 | 953.05 | 1037.48 | 1004.98 | 566.26 | 869.10 | 917.94 | 951.75 | 1035.10 | 968.23 |
| TG 52:3\|TG 16:0_18:1_18:2 | 797.77 | 817.18 | 608.73 | 741.21 | 904.52 | 924.46 | 947.61 | 925.36 | 784.63 | 763.11 | 538.93 | 695.10 | 848.20 | 870.76 | 611.01 | 776.64 |
| TG 52:4\|TG 16:1_18:1_18:2 | 376.27 | 398.90 | 390.02 | 388.37 | 547.17 | 578.24 | 496.49 | 540.38 | 380.08 | 375.40 | 419.91 | 391.43 | 484.05 | 525.23 | 336.79 | 448.65 |
| TG 52:5\|TG 16:0_18:2_18:3 | 71.68 | 74.05 | 45.41 | 63.71 | 160.50 | 169.47 | 161.20 | 163.65 | 69.32 | 62.66 | 60.42 | 64.09 | 126.08 | 129.22 | 70.47 | 108.59 |
| TG 52:6\|TG 14:0_16:0_22:6 | 21.44 | 22.13 | 23.86 | 22.48 | 36.38 | 37.94 | 44.14 | 39.47 | 110.01 | 113.70 | 128.69 | 117.35 | 131.73 | 132.84 | 88.62 | 117.73 |
| TG 53:0\|TG 17:0_18:0_18:0 | 38.38 | 39.20 | 25.30 | 34.29 | 16.97 | 18.29 | 15.54 | 16.92 | 31.83 | 29.89 | 27.08 | 29.58 | 17.14 | 17.95 | 17.27 | 17.45 |
| TG 53:1\|TG 17:0_18:0_18:1 | 117.52 | 123.52 | 99.16 | 113.40 | 74.28 | 80.23 | 43.86 | 66.08 | 105.72 | 100.82 | 56.01 | 87.47 | 70.15 | 71.80 | 36.42 | 59.46 |
| TG 53:2\|TG 17:0_18:1_18:1 | 154.03 | 157.94 | 150.87 | 154.28 | 152.79 | 161.10 | 97.95 | 137.21 | 163.55 | 166.26 | 116.61 | 148.70 | 163.67 | 160.09 | 141.98 | 155.25 |
| TG 53:3\|TG 17:0_18:1_18:2 | 72.68 | 76.02 | 52.28 | 66.99 | 93.54 | 98.51 | 56.09 | 82.67 | 84.72 | 83.98 | 84.83 | 84.44 | 97.66 | 96.36 | 92.03 | 95.35 |
| TG 53:4\|TG 17:1_18:1_18:2 | 19.40 | 21.05 | 22.31 | 20.92 | 36.87 | 37.80 | 27.74 | 34.13 | 27.93 | 26.14 | 26.09 | 26.70 | 35.15 | 35.63 | 31.60 | 34.13 |
| TG 53:5\|TG 17:1_17:2_19:2 | 6.17 | 5.87 | 5.46 | 5.83 | 11.76 | 12.85 | 14.68 | 13.09 | 18.00 | 19.02 | 21.60 | 19.52 | 20.62 | 21.13 | 24.54 | 22.10 |
| TG 53:6\|TG 15:0_16:0_22:6 | 2.51 | 2.47 | 2.52 | 2.50 | 5.49 | 5.77 | 2.86 | 4.70 | 16.22 | 16.21 | 10.72 | 14.37 | 18.57 | 19.01 | 10.65 | 16.08 |
| TG 54:0\|TG 16:0_18:0_20:0 | 72.08 | 70.00 | 70.51 | 70.87 | 23.12 | 24.09 | 13.45 | 20.22 | 40.57 | 40.29 | 39.01 | 39.92 | 20.51 | 21.78 | 16.85 | 19.71 |
| TG 54:1\|TG 18:0_18:0_18:1 | 316.63 | 343.73 | 332.46 | 330.92 | 172.02 | 178.49 | 175.83 | 175.40 | 196.67 | 198.94 | 160.64 | 185.27 | 129.35 | 140.88 | 99.75 | 123.32 |
| TG 54:2\|TG 18:0_18:1_18:1 | 523.58 | 562.67 | 480.06 | 522.07 | 436.56 | 456.84 | 290.77 | 394.56 | 392.81 | 369.31 | 358.76 | 373.33 | 300.35 | 315.13 | 371.51 | 328.98 |
| TG 54:3\|TG 18:0_18:1_18:2 | 535.49 | 556.46 | 438.00 | 509.96 | 734.06 | 730.05 | 590.71 | 684.97 | 505.15 | 494.21 | 370.57 | 456.32 | 462.48 | 476.86 | 566.04 | 501.78 |
| TG 54:4\|TG 18:1_18:1_18:2 | 423.30 | 430.76 | 465.18 | 439.74 | 690.50 | 696.00 | 811.86 | 732.74 | 423.79 | 411.71 | 336.72 | 390.45 | 488.27 | 500.04 | 493.08 | 493.79 |
| TG 54:5\|TG 18:1_18:2_18:2 | 216.26 | 220.28 | 129.33 | 188.62 | 490.86 | 497.00 | 482.13 | 489.95 | 286.81 | 293.28 | 243.89 | 274.44 | 399.52 | 433.75 | 443.69 | 425.62 |
| TG 54:6\|TG 18:1_18:2_18:3 | 28.89 | 29.56 | 17.82 | 25.42 | 179.83 | 183.20 | 100.39 | 154.45 | 45.13 | 34.29 | 46.01 | 41.78 | 99.10 | 107.12 | 53.43 | 86.54 |
| TG 55:0\|TG 16:0_18:0_21:0 | 20.49 | 21.38 | 10.86 | 17.58 | 7.67 | 7.98 | 4.44 | 6.69 | 15.51 | 15.62 | 17.16 | 16.08 | 8.12 | 8.69 | 4.66 | 7.16 |
| TG 55:1\|TG 16:0_21:0_18:1 | 57.76 | 61.59 | 59.85 | 59.73 | 39.56 | 41.42 | 29.23 | 36.72 | 52.66 | 52.33 | 57.93 | 54.25 | 38.17 | 40.27 | 32.21 | 36.88 |
| TG 55:2\|TG 18:0_18:1_19:1 | 43.38 | 45.30 | 53.87 | 47.52 | 39.45 | 41.85 | 25.54 | 35.59 | 45.81 | 41.97 | 30.83 | 39.51 | 42.58 | 42.48 | 27.24 | 37.43 |
| TG 55:3\|TG 18:1_18:1_19:1 | 22.01 | 22.26 | 17.15 | 20.47 | 25.95 | 26.10 | 30.79 | 27.61 | 24.27 | 22.88 | 26.43 | 24.50 | 24.02 | 24.24 | 24.28 | 24.18 |
| TG 55:4\|TG 18:1_19:1_18:2 | 6.71 | 6.24 | 6.07 | 6.34 | 8.49 | 8.81 | 6.48 | 7.92 | 8.60 | 7.91 | 4.41 | 6.97 | 10.04 | 10.20 | 6.72 | 8.99 |
| TG 55:6\|TG 17:0_18:1_20:5 | 2.82 | 3.16 | 3.47 | 3.15 | 5.64 | 6.11 | 4.10 | 5.28 | 19.51 | 18.40 | 17.02 | 18.30 | 20.77 | 21.78 | 16.67 | 19.74 |
| TG 55:7\|TG 15:0_18:1_22:6 | 1.12 | 1.20 | 1.35 | 1.22 | 3.76 | 3.65 | 2.62 | 3.34 | 12.78 | 12.54 | 15.85 | 13.71 | 16.33 | 17.23 | 9.72 | 14.43 |
| TG 56:0\|TG 16:0_18:0_22:0 | 26.70 | 25.86 | 19.57 | 24.04 | 9.55 | 10.03 | 9.34 | 9.64 | 22.09 | 21.35 | 22.94 | 22.10 | 11.20 | 12.66 | 11.78 | 11.88 |
| TG 56:1\|TG 16:0_22:0_18:1 | 107.91 | 111.85 | 111.41 | 110.39 | 59.02 | 60.73 | 41.47 | 53.73 | 73.89 | 73.90 | 68.89 | 72.17 | 49.63 | 50.61 | 38.40 | 46.21 |
| TG 56:2\|TG 16:0_18:1_22:1 | 139.51 | 148.82 | 84.91 | 124.41 | 85.87 | 88.91 | 86.67 | 87.12 | 79.93 | 76.92 | 93.28 | 83.29 | 68.71 | 71.05 | 54.69 | 64.82 |
| TG 56:3\|TG 16:0_18:1_22:2 | 84.13 | 92.94 | 88.30 | 88.45 | 74.80 | 80.10 | 67.56 | 74.11 | 60.53 | 55.15 | 36.08 | 50.56 | 54.47 | 57.71 | 62.76 | 58.31 |
| TG 56:4\|TG 16:0_20:2_20:2 | 45.14 | 48.01 | 41.10 | 44.75 | 58.87 | 60.27 | 63.58 | 60.90 | 51.87 | 49.83 | 59.06 | 53.53 | 43.93 | 47.12 | 32.02 | 41.02 |
| TG 56:5\|TG 16:0_18:1_22:4 | 35.96 | 36.99 | 40.20 | 37.71 | 69.43 | 72.18 | 40.93 | 60.83 | 88.35 | 85.84 | 90.35 | 88.10 | 96.47 | 100.22 | 86.82 | 94.50 |
| TG 56:7\|TG 16:0_18:1_22:6 | 34.82 | 35.18 | 32.24 | 34.08 | 83.55 | 86.81 | 100.62 | 90.30 | 325.48 | 337.61 | 267.39 | 309.91 | 388.25 | 406.30 | 237.59 | 344.03 |
| TG 56:8\|TG 16:0_18:2_22:6 | 9.76 | 9.97 | 8.08 | 9.27 | 31.62 | 33.73 | 37.75 | 34.35 | 113.43 | 115.55 | 124.54 | 117.73 | 217.98 | 218.97 | 118.80 | 185.25 |
| TG 57:0\|TG 16:0_18:0_23:0 | 8.38 | 9.07 | 9.85 | 9.10 | 3.03 | 3.21 | 1.62 | 2.62 | 7.16 | 7.50 | 6.94 | 7.19 | 3.68 | 4.36 | 3.84 | 3.96 |
| TG 57:1\|TG 16:0_23:0_18:1 | 28.78 | 30.34 | 23.31 | 27.47 | 13.65 | 14.84 | 12.74 | 13.74 | 21.33 | 21.30 | 20.31 | 20.96 | 14.56 | 15.58 | 8.25 | 12.80 |
| TG 57:2\|TG 21:0_18:1_18:1 | 27.42 | 28.50 | 28.33 | 28.08 | 20.01 | 20.57 | 13.17 | 17.91 | 21.20 | 21.09 | 19.36 | 20.54 | 19.42 | 20.06 | 11.31 | 16.93 |
| TG 57:3\|TG 21:0_18:1_18:2 | 10.76 | 11.58 | 12.74 | 11.69 | 12.21 | 13.19 | 10.86 | 12.08 | 8.88 | 8.50 | 10.77 | 9.37 | 10.05 | 10.27 | 8.73 | 9.68 |
| TG 57:4\|TG 17:0_18:1_22:3 | 3.62 | 3.20 | 2.73 | 3.19 | 6.22 | 6.41 | 4.55 | 5.73 | 5.82 | 5.24 | 4.43 | 5.16 | 5.76 | 6.64 | 5.25 | 5.88 |
| TG 57:6\|TG 17:0_18:1_22:5 | 1.23 | 1.37 | 1.36 | 1.32 | 2.73 | 2.90 | 1.56 | 2.40 | 10.96 | 11.50 | 9.25 | 10.57 | 13.73 | 13.10 | 13.39 | 13.41 |
| TG 57:7\|TG 17:0_18:1_22:6 | 0.87 | 1.01 | 0.92 | 0.93 | 3.12 | 3.50 | 2.07 | 2.89 | 12.37 | 12.38 | 7.07 | 10.60 | 15.66 | 16.21 | 18.16 | 16.67 |
| TG 57:8\|TG 17:0_18:2_22:6 | 0.49 | 0.39 | 0.59 | 0.49 | 1.39 | 1.39 | 0.98 | 1.25 | 6.30 | 6.30 | 7.92 | 6.83 | 8.81 | 9.35 | 10.23 | 9.46 |
| TG 58:0\|TG 16:0_18:0_24:0 | 6.97 | 7.40 | 8.56 | 7.64 | 2.59 | 2.80 | 1.64 | 2.34 | 5.93 | 6.29 | 5.06 | 5.75 | 3.31 | 4.00 | 2.98 | 3.43 |
| TG 58:1\|TG 16:0_24:0_18:1 | 31.70 | 32.82 | 25.71 | 30.08 | 16.00 | 16.91 | 13.23 | 15.37 | 27.68 | 28.27 | 15.17 | 23.69 | 16.92 | 18.33 | 9.73 | 14.99 |
| TG 58:10\|TG 18:2_18:2_22:6 | 1.22 | 1.19 | 0.69 | 1.03 | 4.69 | 5.05 | 5.31 | 5.01 | 20.96 | 21.01 | 10.86 | 17.60 | 39.65 | 41.65 | 24.28 | 35.19 |
| TG 58:11\|TG 16:0_20:5_22:6 | 0.36 | 0.38 | 0.21 | 0.32 | 0.87 | 0.90 | 0.71 | 0.83 | 4.91 | 5.02 | 6.02 | 5.31 | 8.46 | 9.46 | 6.38 | 8.10 |
| TG 58:2\|TG 18:0_18:1_22:1 | 45.97 | 50.12 | 39.76 | 45.28 | 27.34 | 28.59 | 34.87 | 30.26 | 31.71 | 30.96 | 34.09 | 32.23 | 23.88 | 25.07 | 15.98 | 21.64 |
| TG 58:3\|TG 18:1_18:1_22:1 | 40.89 | 43.30 | 27.16 | 37.12 | 22.70 | 22.92 | 19.91 | 21.84 | 17.34 | 16.36 | 18.56 | 17.41 | 15.87 | 16.21 | 10.55 | 14.21 |
| TG 58:4\|TG 18:1_18:1_22:2 | 26.72 | 28.83 | 13.55 | 23.03 | 23.52 | 23.85 | 29.83 | 25.73 | 15.19 | 14.78 | 19.02 | 16.31 | 11.98 | 12.66 | 12.26 | 12.30 |
| TG 58:8\|TG 18:1_18:1_22:6 | 7.81 | 7.88 | 6.66 | 7.45 | 33.26 | 33.81 | 25.09 | 30.72 | 84.92 | 88.63 | 67.74 | 80.37 | 118.72 | 123.50 | 117.80 | 120.00 |
| TG 58:9\|TG 18:1_18:2_22:6 | 3.64 | 3.84 | 3.65 | 3.71 | 18.82 | 20.34 | 9.67 | 16.26 | 51.22 | 50.16 | 62.14 | 54.45 | 89.27 | 97.21 | 101.81 | 96.09 |
| TG 59:0\|TG 16:0_18:0_25:0 | 2.08 | 2.08 | 1.77 | 1.98 | 0.72 | 0.73 | 0.71 | 0.72 | 1.61 | 1.64 | 1.66 | 1.64 | 0.79 | 0.84 | 0.83 | 0.82 |
| TG 59:1\|TG 16:0_25:0_18:1 | 8.37 | 9.02 | 6.30 | 7.89 | 4.28 | 4.36 | 4.74 | 4.46 | 7.73 | 7.98 | 4.20 | 6.63 | 4.86 | 5.35 | 4.91 | 5.04 |
| TG 59:11\|TG 15:0_22:5_22:6 | 0.00 | 0.00 | 0.00 | 0.00 | 0.08 | 0.09 | 0.08 | 0.09 | 0.63 | 0.64 | 0.56 | 0.61 | 0.83 | 0.81 | 0.62 | 0.75 |
| TG 59:2\|TG 23:0_18:1_18:1 | 8.95 | 9.93 | 9.03 | 9.30 | 6.09 | 6.60 | 6.87 | 6.52 | 7.99 | 7.80 | 8.34 | 8.03 | 6.50 | 6.90 | 7.13 | 6.85 |
| TG 59:3\|TG 23:0_18:1_18:2 | 4.04 | 4.46 | 2.59 | 3.70 | 4.16 | 4.13 | 4.01 | 4.10 | 3.82 | 3.83 | 3.72 | 3.79 | 4.06 | 4.30 | 4.42 | 4.26 |
| TG 60:0\|TG 16:0_18:0_26:0 | 1.44 | 1.61 | 0.83 | 1.29 | 0.58 | 0.61 | 0.66 | 0.62 | 1.28 | 1.22 | 1.54 | 1.35 | 0.64 | 0.70 | 0.45 | 0.60 |
| TG 60:12\|TG 16:0_22:6_22:6 | 0.15 | 0.14 | 0.15 | 0.15 | 0.65 | 0.66 | 0.62 | 0.64 | 6.45 | 6.06 | 4.30 | 5.60 | 10.68 | 11.63 | 11.06 | 11.12 |
| TG 60:3\|TG 24:0_18:1_18:2 | 7.55 | 7.96 | 4.05 | 6.52 | 6.43 | 6.31 | 6.84 | 6.53 | 7.23 | 6.95 | 4.21 | 6.13 | 6.32 | 6.56 | 3.57 | 5.49 |
| TG 60:8\|TG 18:1_20:1_22:6 | 0.66 | 0.65 | 0.48 | 0.60 | 1.52 | 1.59 | 0.86 | 1.32 | 4.49 | 4.53 | 3.30 | 4.10 | 4.95 | 5.36 | 3.68 | 4.66 |
| TG 61:12\|TG 17:0_22:6_22:6 | 0.02 | 0.02 | 0.02 | 0.02 | 0.05 | 0.05 | 0.04 | 0.05 | 0.57 | 0.56 | 0.63 | 0.59 | 0.68 | 0.67 | 0.75 | 0.70 |
| TG 61:3\|TG 25:0_18:1_18:2 | 1.43 | 1.53 | 1.20 | 1.39 | 1.48 | 1.52 | 1.74 | 1.58 | 1.50 | 1.52 | 1.49 | 1.50 | 1.52 | 1.63 | 1.50 | 1.55 |
| TG 62:12\|TG 18:1_22:5_22:6 | 0.04 | 0.05 | 0.02 | 0.03 | 0.58 | 0.61 | 0.73 | 0.64 | 3.14 | 3.16 | 3.49 | 3.26 | 4.95 | 5.32 | 3.72 | 4.66 |
| TG 62:13\|TG 18:1_22:6_22:6 | 0.05 | 0.05 | 0.04 | 0.05 | 0.32 | 0.36 | 0.37 | 0.35 | 2.31 | 2.39 | 1.30 | 2.00 | 3.98 | 4.13 | 2.47 | 3.53 |
| TG 62:14\|TG 18:2_22:6_22:6 | 0.01 | 0.01 | 0.01 | 0.01 | 0.09 | 0.09 | 0.07 | 0.08 | 0.49 | 0.51 | 0.31 | 0.44 | 1.01 | 1.03 | 0.58 | 0.87 |
| TG 62:7\|TG 16:0_24:1_22:6 | 0.42 | 0.44 | 0.25 | 0.37 | 0.65 | 0.73 | 0.48 | 0.62 | 1.59 | 1.44 | 1.54 | 1.52 | 1.71 | 1.83 | 1.68 | 1.74 |
| TG 63:6\|TG 16:0_25:0_22:6 | 0.07 | 0.06 | 0.04 | 0.06 | 0.13 | 0.14 | 0.11 | 0.13 | 0.78 | 0.75 | 0.72 | 0.75 | 0.89 | 0.96 | 1.01 | 0.95 |
| TG 64:0\|TG 16:0_18:0_30:0 | 0.12 | 0.12 | 0.12 | 0.12 | 0.11 | 0.12 | 0.13 | 0.12 | 0.10 | 0.11 | 0.06 | 0.09 | 0.07 | 0.07 | 0.05 | 0.06 |
| TG 64:3\|TG 28:0_18:1_18:2 | 0.45 | 0.49 | 0.33 | 0.42 | 0.37 | 0.40 | 0.22 | 0.33 | 0.40 | 0.44 | 0.40 | 0.42 | 0.48 | 0.45 | 0.26 | 0.40 |
| TG 64:7\|TG 16:0_26:1_22:6 | 0.06 | 0.05 | 0.03 | 0.05 | 0.70 | 0.73 | 0.46 | 0.63 | 0.87 | 0.90 | 0.99 | 0.92 | 0.16 | 0.16 | 0.18 | 0.16 |
| TG 65:0\|TG 16:0_23:0_26:0 | 0.04 | 0.05 | 0.03 | 0.04 | 0.05 | 0.06 | 0.04 | 0.05 | 0.07 | 0.08 | 0.08 | 0.07 | 0.03 | 0.03 | 0.02 | 0.03 |
| TG 65:1\|TG 16:0_31:0_18:1 | 0.10 | 0.11 | 0.05 | 0.09 | 0.09 | 0.10 | 0.10 | 0.10 | 0.06 | 0.07 | 0.05 | 0.06 | 0.06 | 0.06 | 0.06 | 0.06 |
| TG 65:2\|TG 29:0_18:1_18:1 | 0.11 | 0.11 | 0.10 | 0.11 | 0.09 | 0.10 | 0.07 | 0.08 | 0.07 | 0.08 | 0.08 | 0.08 | 0.08 | 0.08 | 0.09 | 0.08 |
| TG 65:3\|TG 29:0_18:1_18:2 | 0.08 | 0.08 | 0.07 | 0.07 | 0.06 | 0.07 | 0.05 | 0.06 | 0.06 | 0.06 | 0.07 | 0.06 | 0.07 | 0.06 | 0.04 | 0.06 |
| TG 66:7\|TG 26:0_18:1_22:6 | 0.00 | 0.00 | 0.00 | 0.00 | 0.17 | 0.18 | 0.10 | 0.15 | 0.24 | 0.25 | 0.25 | 0.25 | 0.04 | 0.04 | 0.03 | 0.04 |
